# Gray and white matter alterations in Obsessive-Compulsive Personality Disorder: a data fusion machine learning approach

**DOI:** 10.3389/fnhum.2025.1559760

**Published:** 2025-05-14

**Authors:** Lorenzo Arena, Wenceslao Peñate, Francisco Rivero, Rosario J. Marrero, Teresa Olivares, Alessandro Scarano, Ascensión Fumero, Alessandro Grecucci

**Affiliations:** ^1^Department of Psychology and Cognitive Science, University of Trento, Trento, Italy; ^2^Departamento de Psicología Clínica, Psicobiología y Metodología, Facultad de Psicología, Universidad de La Laguna, La Laguna, Spain; ^3^Instituto Universitario De Neurociencia (IUNE), Universidad de La Laguna, La Laguna, Spain; ^4^Facultad de Ciencias de la Salud, Universidad Europea de Canarias, La Orotava, Spain

**Keywords:** Obsessive-Compulsive Personality Disorder, machine learning, data fusion, gray matter, white matter

## Abstract

**Introduction:**

Obsessive-Compulsive Personality Disorder (OCPD) is a complex mental condition marked by excessive perfectionism, orderliness, and rigidity, often starting in adolescence or early adulthood; it affects 1.9% to 7.8% of the population. The disorder differs from Obsessive-Compulsive Disorder (OCD) in an apparent compromise of personality, distorted self-representation, and altered perception of others. Although the two disorders present evident differences, unlike OCD, the neural bases of OCPD are understudied. The few studies conducted so far have identified gray matter alterations in brain regions such as the striatum and prefrontal cortex. However, a comprehensive model of its neurobiology and the eventual contribution of white matter abnormalities are still unclear. One intriguing hypothesis is that regions ascribed to the Default Mode Network are involved in OCPD, similar to what has been shown for OCD and other anxiety disorders.

**Methods:**

To test this hypothesis, the gray and white matter images of 30 individuals diagnosed with OCPD (73% female, mean age=29.300), and 34 non-OCPD controls (82% female, mean age = 25.599) were analyzed for the first time with a data fusion unsupervised machine learning method known as Parallel Independent Component Analysis (pICA) to detect the joint contribution of these modalities to the OCPD diagnosis.

**Results:**

Results indicated that two gray matter networks (GM-05 and GM-23) and one white matter network (WM-25) differed between the OCPD and the control group. GM-05 included brain regions belonging to the Default Mode Network and the Salience Network and was significantly correlated with anxiety; GM-23 included portions of the cerebellum, the precuneus, and the fusiform gyrus; WM-25 included white matter portions adjacent to Default Mode Network regions.

**Discussion:**

These findings shed new light on the gray and white matter contributions to OCPD and may pave the way to developing objective markers of this disorder.

## Introduction

Obsessive-Compulsive Personality Disorder (OCPD) is a complex mental disorder characterized by a pervasive pattern of perfectionism, excessive orderliness, and rigid conformity to rules and procedures at the expense of psychological flexibility and efficiency, typically emerging in adolescence or early adulthood (American Psychiatric Association, 2013; Marincowitz et al., 2022). Individuals with OCPD exhibit preoccupations with details that lead to missing the major point of the activity, extreme perfectionism that is often the cause of delay, with tasks not being completed, and excessive rigidity that can interfere with leisure and interpersonal relationships (American Psychiatric Association, 2022; Pinto et al., 2022). These characteristics contribute to significant distress and a reduced quality of life. This disorder affects approximately 1.9% to 7.8% of the general population (Grant et al., 2004; Nestadt et al., 1991), with individuals frequently seeking for treatment in mental health and primary care services (Bender et al., 2001; Sansone et al., 2003). Despite its prevalence, OCPD remains understudied (Diedrich and Voderholzer, 2015; Pinto et al., 2022), especially when compared to Obsessive-Compulsive disorder (OCD), which has been extensively investigated (Maia et al., 2008; Mataix-Cols and van den Heuvel, 2006; Nakao et al., 2014; Robbins et al., 2024; Stein et al., 2019). Neuroimaging studies on OCD have consistently highlighted the involvement of the orbitofrontal cortex, the cingulate cortex—a hub of the Default Mode Network (DMN)—and the head of the caudate nucleus, whose hyperactivation appears to contribute to compulsive behaviors typical of OCD (Del Casale et al., 2015; Maia et al., 2008; Rasgon et al., 2017; Stein et al., 2019). Additionally, OCD is often associated with altered functional activation and reduced morphometric properties in regions implicated in cognitive control, such as the dorsomedial prefrontal cortex, a key node in the Central Executive Network (De Wit et al., 2014; Pujol et al., 2004; Radua and Mataix-Cols, 2009; Rasgon et al., 2017). Structural abnormalities have also been observed in the insulo-opercular region, anterior cerebellum, and bilateral ventral putamen, with volumetric reductions in OCD patients compared to healthy controls (Pujol et al., 2004). Furthermore, altered brain connectivity patterns, such as hyperconnectivity between the lateral parietal lobe and clusters extending into the precuneus and superior lateral occipital cortex, have been reported in OCD (Geffen et al., 2022). It is plausible to hypothesize that OCPD might exhibit neural alterations similar to those observed in OCD. Indeed, some studies have highlighted commonalities, such as alterations in the dorsolateral prefrontal cortex (DLPFC) in OCPD (Atmaca et al., 2019b). However, distinct neural patterns have also been reported: while OCD is predominantly associated with orbitofrontal cortex dysfunction (Pauls et al., 2014), OCPD may involve hyperactivity in the anterior cingulate cortex (Fineberg et al., 2018). Importantly, unlike OCD, OCPD is not necessarily characterized by overt compulsions but rather by personality-level traits, such as rigid self-representation and altered perceptions of others. Neuroimaging evidence suggests both shared and distinct mechanisms underlying these disorders (Marincowitz et al., 2022).

For instance, resting state functional magnetic resonance (fMRI) has revealed increased functional connectivity in the precuneus, the posterior hub of the DMN, of individuals with OCPD when compared to healthy controls (Coutinho et al., 2016). Another study by Lei et al. (2020) found increased amplitudes of low-frequency fluctuation (ALFF) inside the caudate, the precuneus, the insula, and the medial superior frontal gyrus in individuals with OCPD, while decreased ALFF was detected inside the fusiform and lingual gyri. Structural alterations have also been reported: individuals with OCPD exhibit reduced hippocampal and amygdala volumes compared to healthy controls (Atmaca et al., 2019a; Gurok et al., 2019). While changes in the insula, caudate, and amygdala have been observed in both OCPD and OCD, precuneus and hippocampus alterations may be more specific to OCPD (Marincowitz et al., 2022). Alterations of the caudate tail, ventral striatum, and prefrontal cortex have been reported to be probably associated with the disorder, but the main focus of the study that supports this hypothesis is on Cluster C personality disorders in general (Payer et al., 2015). More specificity is needed to clearly define the exact contribution of these areas to OCPD. The primary aim of this study is to detect GM abnormalities in OCPD compared to controls to specifically test the hypothesis that the regions ascribed to the DMN are affected.

In sum, the evidence reviewed so far indicates that both common and distinct mechanisms are present in OCPD and OCD, but a reliable and comprehensive model of the neurobiology of OCPD is still lacking in literature (Marincowitz et al., 2022). Research on white matter contributions to OCPD remains sparse. The only study explicitly investigating this found no significant white matter alterations in OCPD patients (Atmaca et al., 2019a). However, a study conducted by Grazioplene et al. (2022) found abnormalities in white matter to be associated with specific obsessive-compulsive symptoms, such as bad thoughts, repetition/checking, and behavioral symmetry, particularly in the posterior corpus callosum, dorsal parietal lobe, and posterior parietal and occipital lobes. Additionally, Laricchiuta et al. (2014) found that cerebellar white matter increment was associated with novelty seeking, while a reduction was associated with harm avoidance, but again, no evidence specific to OCPD was reported. In sum, the evidence relative to white matter contributions to OCPD is limited. Thus, the second aim of the study is to detect white matter abnormalities in OCPD compared to non-OCPD controls.

It is worth noting that the previous studies on OCPD have some methodological limitations. One for all, the use of massive univariate methods such as Voxel-based methodology or *a priori* selected regions of interest (ROIs) (Colic et al., 2018; Gurok et al., 2019; Lei et al., 2020; Payer et al., 2015), which fail to capture the complex, multivariate nature of neural alterations across the whole brain and neglect inter-voxel relationships (Aguilar-Ortiz et al., 2018; Dadomo et al., 2022). Furthermore, they do not account for generalization to new data, limiting their predictive value. In other words, they do not have an estimation of how those results can be used to predict new unobserved cases. To address these issues, recent studies have employed multivariate approaches, such as Multivoxel Pattern Analysis (MVPA) and machine learning (ML) techniques, which offer increased sensitivity in detecting spatially distributed neural patterns (Scarano et al., 2024; Norman et al., 2006). Data fusion methods, in particular, integrate multiple neuroimaging modalities, allowing for the simultaneous analysis of gray and white matter features to identify joint neural circuits implicated in psychiatric conditions (Grecucci et al., 2016; Lapomarda et al., 2021a, 2021b; Saviola et al., 2020; Sorella et al., 2019). These techniques have been applied to other personality disorders, such as borderline (Caria and Grecucci, 2023; Dadomo et al., 2022; Grecucci et al., 2022, 2023), narcissistic (Jornkokgoud et al., 2023, 2024), and antisocial personality disorders (Sorella et al., 2022). The joint contribution of GM-WM alterations may reveal a more complete picture of the neural alterations in OCPD.

To the best of our knowledge, no previous study has applied a data fusion (GM-WM) machine learning approach to investigate OCPD. Therefore, our study aims to offer novel and compelling insights into the neural bases of OCPD through data fusion and unsupervised machine learning techniques. The machine learning method employed in this study is a specialized form of Independent Component Analysis (ICA) (Lee, 1998) known as Parallel ICA (pICA) (Liu et al., 2008; Liu et al., 2009). This approach enables simultaneous analysis of two modalities (e.g., gray matter and white matter), assessing their interrelationships while decomposing brain data into naturally grouped networks with reduced dimensionality (Yang et al., 2019). This method optimizes the extraction of relevant information for analysis. Data fusion, in particular, allows for the integrated analysis of gray and white matter without losing the connections between the two modalities. This is especially important given the likelihood that these modalities are influenced by shared genetic factors and that both may play a role in the development of OCPD.

We predict alterations in regions ascribed to the Default Mode Network (DMN), such as the precuneus and medial frontal gyrus. These regions may be linked to the abnormal self and other representations observed in OCPD, as well as anxious rumination. The DMN is a network of brain areas that includes the retrosplenial cortex, inferior parietal cortex, dorsolateral frontal cortex, inferior frontal cortex, left inferior temporal gyrus, medial frontal regions, and amygdala (Alves et al., 2019). It is known to support internally oriented cognitive functions (Yeshurun et al., 2021), self-referential processing, and pain processing. These functions are often disrupted in personality disorders (Kluetsch et al., 2012). We also predict alterations in regions belonging to the Salience Network, such as the cingulate gyrus. These changes may underlie the heightened processing of anxiety-laden stimuli (Grahn et al., 2008; Uddin et al., 2017) that is characteristic of OCPD. The hypothesis of DMN involvement in OCD has already been investigated by Gonçalves and colleagues, who found no patterns of DMN deactivation in subjects with OCD during the presentation of unpleasant stimuli, whereas a minor deactivation was observed during the presentation of pleasant stimuli (Gonçalves et al., 2017). In that study, patients with OCD, relative to non-OCPD controls, exhibited difficulties in DMN deactivation. Given this evidence and the functional similarities between OCD and OCPD, predicting DMN involvement in OCPD seems reasonable and represents a hypothesis worth investigating.

Research on white matter contributions to OCPD remains sparse. However, we expect white matter alterations in regions associated with the Default Mode Network (DMN), such as the precuneus and medial frontal gyrus, which may underlie abnormal self and other representations in OCPD, as well as anxious rumination. Additionally, alterations are anticipated in regions belonging to the Salience Network, such as the cingulate gyrus, potentially contributing to the heightened processing of anxiety-laden stimuli characteristic of OCPD. These predictions align with evidence of DMN involvement in OCD, where difficulties in DMN deactivation have been observed. Given the functional similarities between OCD and OCPD, investigating DMN alterations in OCPD is a compelling hypothesis worth exploring.

Clarifying these neural alterations could advance our understanding of the distinct neurobiological mechanisms underpinning OCPD and inform targeted therapeutic interventions.

## Materials and methods

### Participants

Participants included in the sample were 64 right-handed adults (22% male and 78% female, aged 18 to 56 years, average = 28.37 Sd = 10.716) residents in Tenerife (Canary Island, Spain). Neither sex nor age were normally distributed, according to the Shapiro–Wilk test for normality (Sex: w = 0.790, *p*-value < 0.001 Age: w = 0.510, *p*-value < 0.001). The participants were originally recruited for studying small animal phobias and their relations with Cluster C personalities (see also Grecucci et al., 2023). In the present study, as our focus was on OCPD, we divided participants into two groups according to the diagnosis of OCPD. This was done according to a cut-off criteria of the International Personality Disorder Examination (IPDE) (Loranger, 1994). The resulting sample was of 34 controls without OCPD (CTRL, *M_age_* = 27.559, *SD_age_* = 10.810) and 30 participants with OCPD (OCPD, *M_age_* = 29.300, *SD_age_* = 10.710), matched for sex (*χ^2^* = −0.759, *p* = 0.384) and age (W = −580.500, *p* = 0.342) (*Mann–Whitney test*). Of note, the presence of phobias was similarly distributed within OCPD and non-OCPD controls (*χ^2^* = 2.259, *p* = 0.133). Refer to Table 1 for a more detailed understanding of the participants’ demographics. The exclusion criteria considered the following characteristics for both groups: impediments to undergoing magnetic resonance imaging, such as metallic implants, braces, non-removable piercings, tattoos, pregnancy, claustrophobia, tinnitus, or any surgical operation in the past 3 months. This study adhered to the ethical standards of the Declaration of Helsinki and was approved by the Ethics Committee for Research and Animal Welfare of the University of La Laguna, Spain (CEIBA2013-0086).

**Table 1:** For participants’ demographics, the *p*-value is calculated using the chi-squared test for the sex and phobia variables, and the Mann–Whitney test for all other variables.

Measurement	OCPD	CTRL	*p*-value
*N*.	30	34	
Sex	8 M, 22 F	6 M, 28 F	0.384
Age	29.3 (±10.7)	25.5 (±10.8)	0.342
Phobia group	18 phobics, 12 non-phobics	14 phobics, 20 non-phobics	0.133
Average IPDE score	3.967	1.176	<0.001
Average S-R score	25.667	19.941	0.290
Average HAD anxiety	7.233	4.588	0.009
Average HAD depression	4.522	2.912	0.167
Average BAI	13.633	8.647	0.045
Average HARS	12.900	7.824	0.027

The table also includes data related to psychological scales that are better explained in the questionnaire section.

### Questionnaires

The participants completed questionnaires to assess various psychological and physiological characteristics. The Hamilton Anxiety Rating Scale (HARS) (Hamilton, 1959; Bruss et al., 1994) is a 14-item clinician-administered tool used to assess the severity of anxiety symptoms. Each item is rated on a 5-point Likert scale ranging from 0 (not present) to 4 (very severe). The scale demonstrates good inter-rater reliability, with intraclass correlation coefficients ranging from 0.74 to 0.96 (Bruss et al., 1994; Thompson, 2015). The S–R (Situation–Response) Inventory of Anxiousness (Endler et al., 1962) was administered to participants in both groups (with and without phobia). This 14-item inventory uses a 5-point Likert scale to evaluate the most common physiological, cognitive, and behavioral symptoms associated with responses to anxiogenic stimuli, such as cockroaches, spiders, lizards, or mice. It has demonstrated high internal consistency (Cronbach’s alpha = 0.95) and adequate convergent validity (Kameoka and Tanaka-Matsumi, 1981). The Edinburgh Handedness Inventory (Oldfield, 1971) was used to confirm that all participants were right-handed. This 10-item inventory employs a forced-choice format, with scores above 0 indicating a right-hand preference and scores below 0 indicating a left-hand preference. The Beck Anxiety Inventory (BAI) (Beck et al., 1988) is a 21-item self-report questionnaire used to discriminate anxiety disorders from depressive disorders in psychiatric patients. The items are 4-point scales that consist of the perceived rate of how much the patient has been bothered by each symptom over the past week. The BAI showed high internal consistency (alpha = 0.92) and test–retest reliability over 1 week, r(81) = 0.75. The Hospital Anxiety and depression scale (HADS) (Zigmond and Snaith, 1983) is a scale used to evaluate the presence of anxiety and depression caseness in the subjects. This 14-item inventory consists of 4-point items ranging from 1 to 4. The scale has good internal consistency (mean Cronbach alpha = 0.83 for Depression and 0.82 for Anxiety, Bjelland et al., 2002). Finally, the International Personality Disorder Examination (IPDE, Loranger, 1997) is an inventory designed to assess the nine personality disorders using a true/false response format. In this study, 8 of the 20 items related to cluster C (avoidant, dependent, and obsessive-compulsive personality disorders) were used to assess obsessive-compulsive personality disorder (OCPD). A cut-off of ≥3 was used to identify participants with OCPD, while controls scored below this threshold. Anxiety scores, measured by HARS, BAI, and HAD, were statistically different between the two groups (see Table 1).

### Data acquisition

High-resolution, three-dimensional, T1-weighted, whole-brain resting state structural MRI images were acquired on a 3.0 T MR scanner with a 12-channel head coil (GE 3.0 T Sigma Excite HD). The subject was instructed to keep their eyes closed, relax, and lie as still as possible. Repetition time (TR)/echo time (TE) = 8,852 ms/1,756 ms, flip angle = 10°, 172 sagittal slices, slice thickness = 1 mm, field of view (FOV) = 256 × 256 mm^2^, data matrix = 256 × 256 × 172, the voxel size was 1 × 1 × 1 mm and TI = 650 ms.

### Preprocessing

After a quality check conducted by an experienced neuroradiologist to rule out visible movement artifacts and gross structural abnormalities, all data were pre-processed using the segmentation routines provided by the Computational Anatomy Toolbox (CAT12),^1^ a toolbox available for the Statistical Parametric Mapping software (SPM12),^2^ for the MATLAB environment. The segmentation was registered using Diffeomorphic Anatomical Registration through Exponential Lie algebra tools (DARTEL) (Ashburner, 2007), a potential alternative to SPM’s traditional registration approaches that operates using a whole-brain approach (Grecucci et al., 2016; Pappaianni et al., 2018; Yassa and Stark, 2009). Also, surface and Thickness were estimated. Finally, Dartel files were normalized to MNI space using a Spatial Gaussian Smoothing of 8 mm.

### Data fusion unsupervised machine learning

Independent Component Analysis is a blind source separation method that allows the finding of latent and independent components in a data set. ICA, in its forms of joint ICA and parallel ICA, is also used to provide a multivariate data fusion approach, capable of maintaining the latent association within data. Parallel ICA is particularly useful when data are assumed to be mixed in similar patterns (Mohammed et al., 2014), making the method particularly suitable for correlation among similar but independent components and across different modalities. For example, it is recognized that gray and white matter, even if different modalities, are probably correlated and share some genetic and biological common origins (Spalletta et al., 2018). We opted to respect this correlation between two different modalities and use a parallel ICA approach for the dimensional decomposition of brain data. The parallel ICA was applied to GM and WM data using the Fusion ICA Toolbox (FIT)^3^ (Calhoun et al., 2006) in MATLAB 2018a environment.^4^ ICA works by converting every structural image into a one-dimensional vector, then using these vectors to build a data matrix. This matrix is then decomposed into a mixing matrix, which indicates the relationship between subjects and the degree to which each component contributes to a given subject, as well as a source matrix. Then, the scores are used to correlate each network with the psychological variable of interest. For more information, see the work of Xu et al. (2009). Parallel ICA conducts an individual ICA for each modality, maximizing independence within each modality and optimizing the correlation between the same modalities. The decomposition of the brain in different components has been conducted in steps: In the first step, an estimation of the number of components for both modalities was conducted with information theoretical criteria (Wax and Kailath, 1985). Then, the ICA was performed using the ICASSO algorithm (Himberg and Hyvarinen, 2003; Himberg et al., 2004) to assess the consistency of each modality. To ensure the reliability of our findings and mitigate the risk of false discoveries due to overfitting, we employed a leave-one-out assessment (Liu et al., 2009). With these settings, the ICASSO algorithm was conducted 100 times with identical parameter settings, excluding one subject in each run, allowing us to assess consistency by examining the results from the 100 repetitions. The software was then used to convert the components into Talairach coordinates, a process necessary to define the brain areas included in each component. The data were then considered in their positive values and plotted in Surf Ice for visualization (Rorden).^5^

### Statistical analysis

A Backward stepwise regression model was run for white and gray matter separately to determine the components with the highest association with OCPD. The decision to run one analysis for each feature was determined to avoid redundancy in the model, given the assumption of correlation between GM and WM (an assumption we could make given the peculiarity of the data fusion approach). We used the loading coefficients from each independent network derived through p-ICA as covariates, keeping the OCPD assessment as the dependent variable.

## Results

### Network decomposition

**Figure 1:**
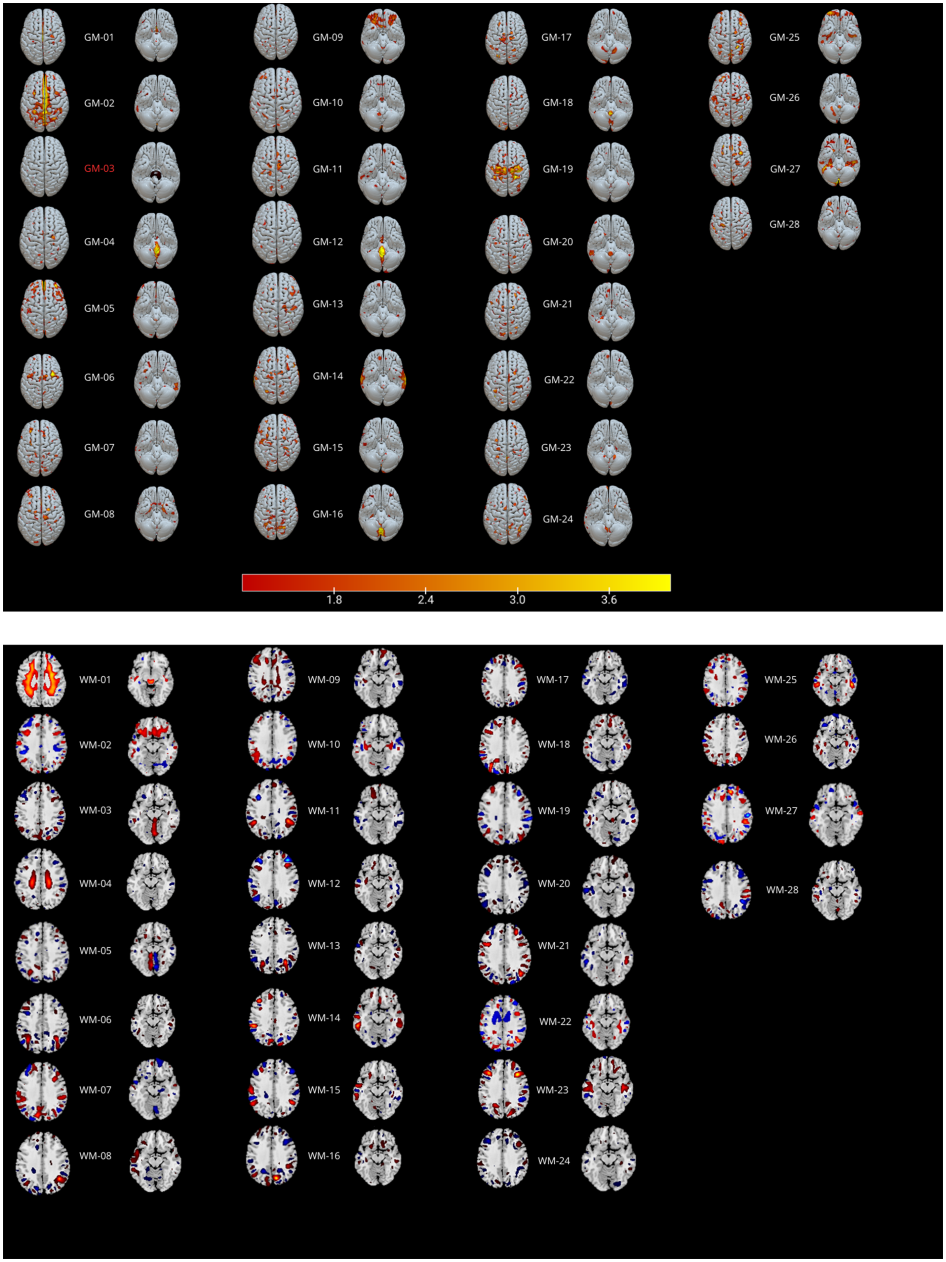
Parallel ICA results. GM ICs are reported in the top panel, WM ICs in the bottom panel. Positive values (in hot colors) indicate increased GM or WM density, while negative values (blue areas) indicate decreased density. See Table 2 for the correlations between modalities.

The information-theoretic criteria estimated 28 independent within-subjects covarying gray (GM) and 28 white (WM) matter networks (see Figure 1 and Table 2), but after a visual inspection of the components, the GM03 component was rejected because it appeared to be an artifact or at least too noisy.

**Table 2:** Correlations between the linked components.

GM	WM	Correlation	T-value	*p*-value
22	6	−0.95726	−26.061	4.159e − 35
4	5	−0.91634	−18.02	2.4892e − 26
1	4	0.88822	15.223	1.2916e − 22
3	1	−0.75384	−9.0339	6.5017e − 13
10	13	−0.7109	−7.9591	4.6591e − 11
12	3	−0.67979	−7.2984	6.5372e − 10
8	26	0.6512	6.7566	5.6632e − 09
11	14	−0.62404	−6.2884	3.6025e − 08
13	11	0.59347	5.806	2.3627e − 07
27	19	0.58998	5.7535	2.8936e − 07
9	25	0.57793	5.5761	5.7171e − 07
26	18	−0.53573	−4.9957	5.073e − 06
17	16	−0.5079	−4.6426	1.8352e − 05
21	7	−0.49643	−4.5029	3.0198e − 05
5	17	0.48894	4.4134	4.1416e − 05
7	24	0.46849	4.1754	9.4553e − 05
18	23	−0.44604	−3.9241	0.00022078
20	12	−0.44297	−3.8905	0.00024685
25	18	0.44065	3.8652	0.00026834
6	9	−0.43831	−3.8397	0.00029179
16	21	0.43322	3.7847	0.00034933
14	20	−0.40744	−3.513	0.00083311
23	1	−0.39909	−3.4272	0.0010881
15	1	0.37687	3.2037	0.0021439
2	17	0.34769	2.9198	0.0048767
24	23	−0.33679	−2.8164	0.0065042
28	12	0.32745	2.7288	0.0082604
19	8	−0.31226	−2.5881	0.012005

### Stepwise regression on loading coefficients

**Figure 2:**
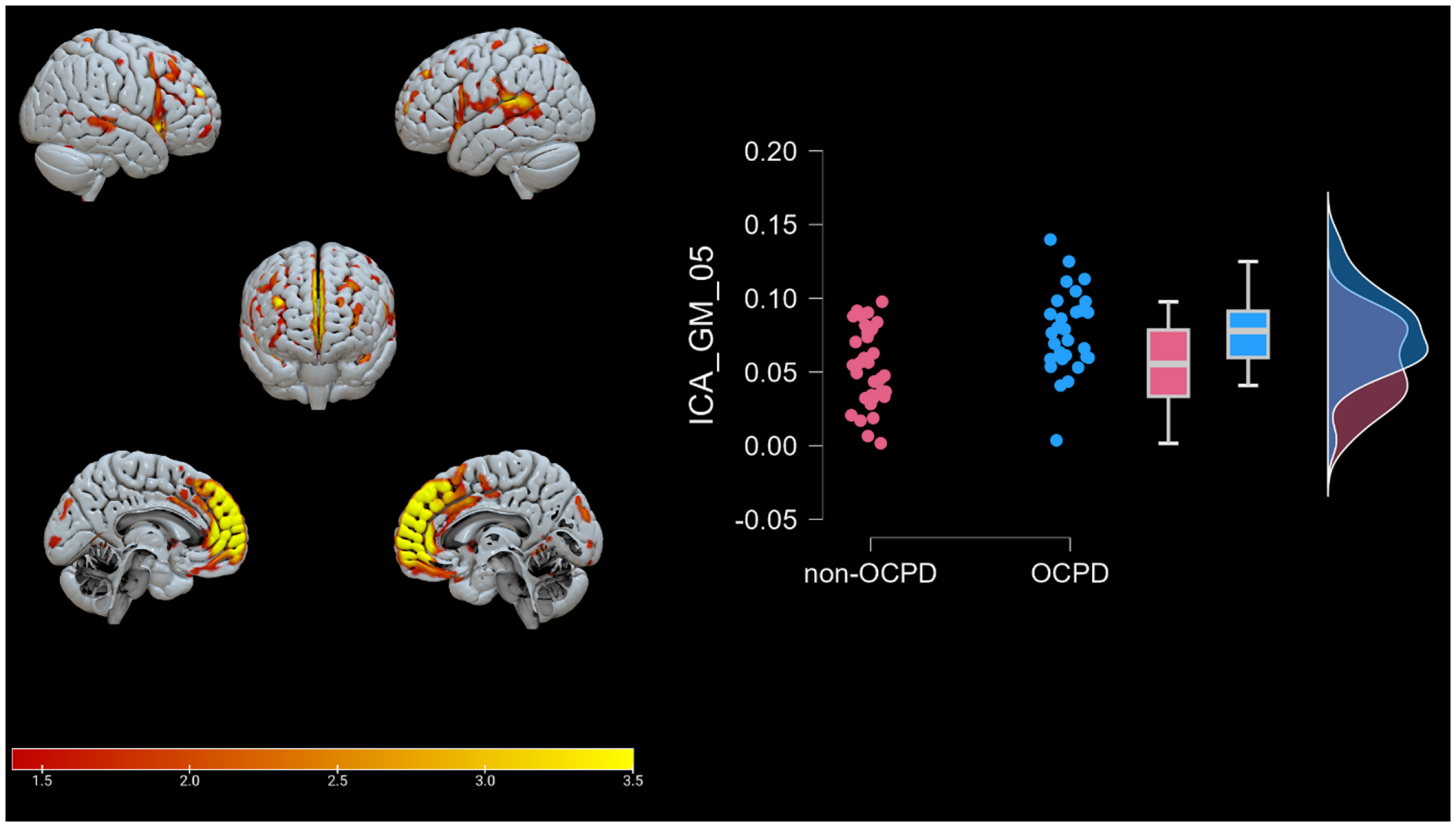
Brain plots of GM-05. Graphical representation of the gray matter areas that differ between individuals with OCPD and non-OCPD controls. Individuals with OCPD show higher gray matter density in this network compared to non-OCPD controls. A raincloud plot of the loading coefficients is also represented.

**Figure 3:**
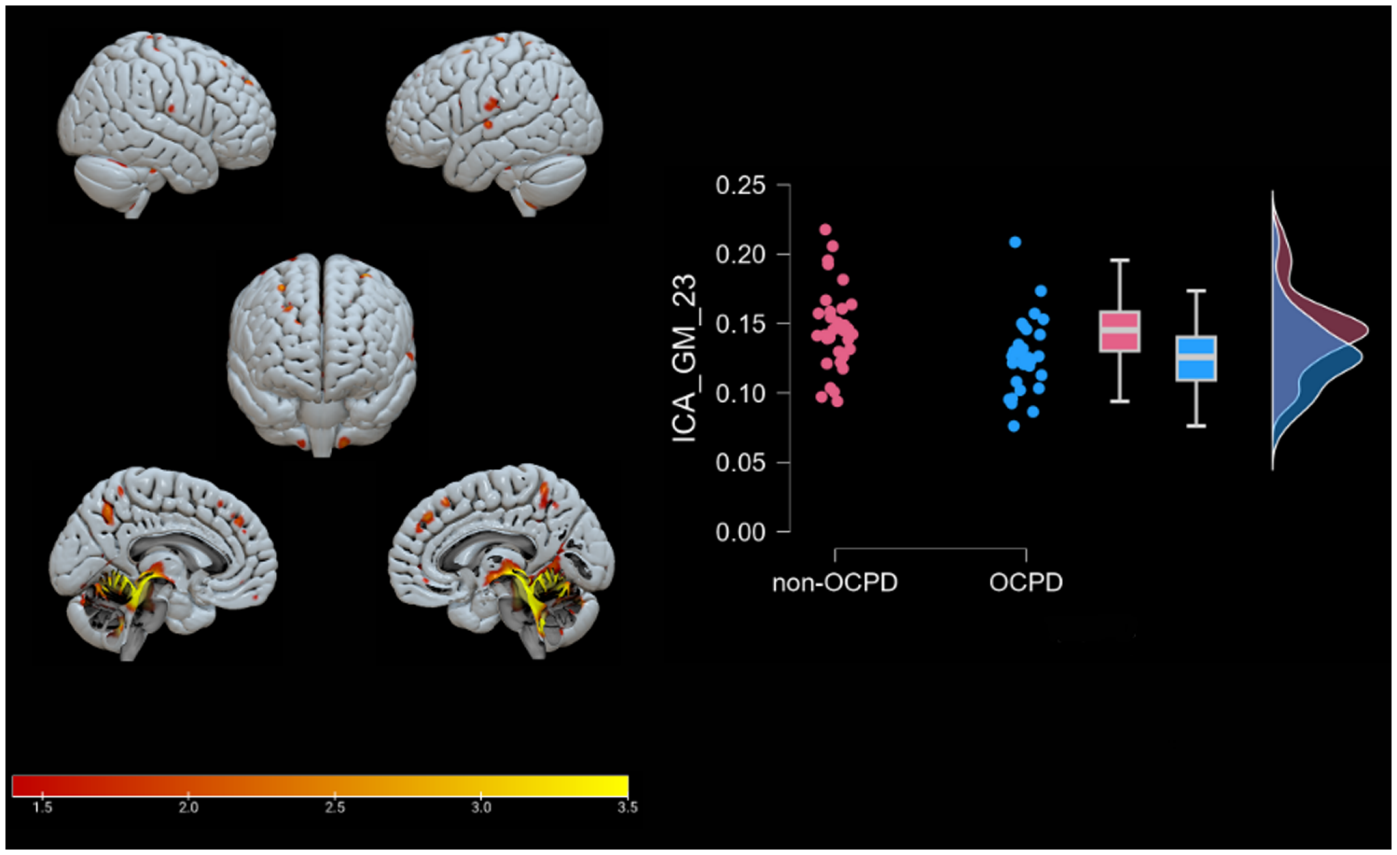
Brain plots of GM-23. Graphical representation of the gray matter areas that differ between OCPD individuals vs. non-OCPD controls. This network shows that OCPD individuals display lower GM density in this network compared to non-OCPD controls. A raincloud plot of the loading coefficients is also represented.

**Figure 4:**
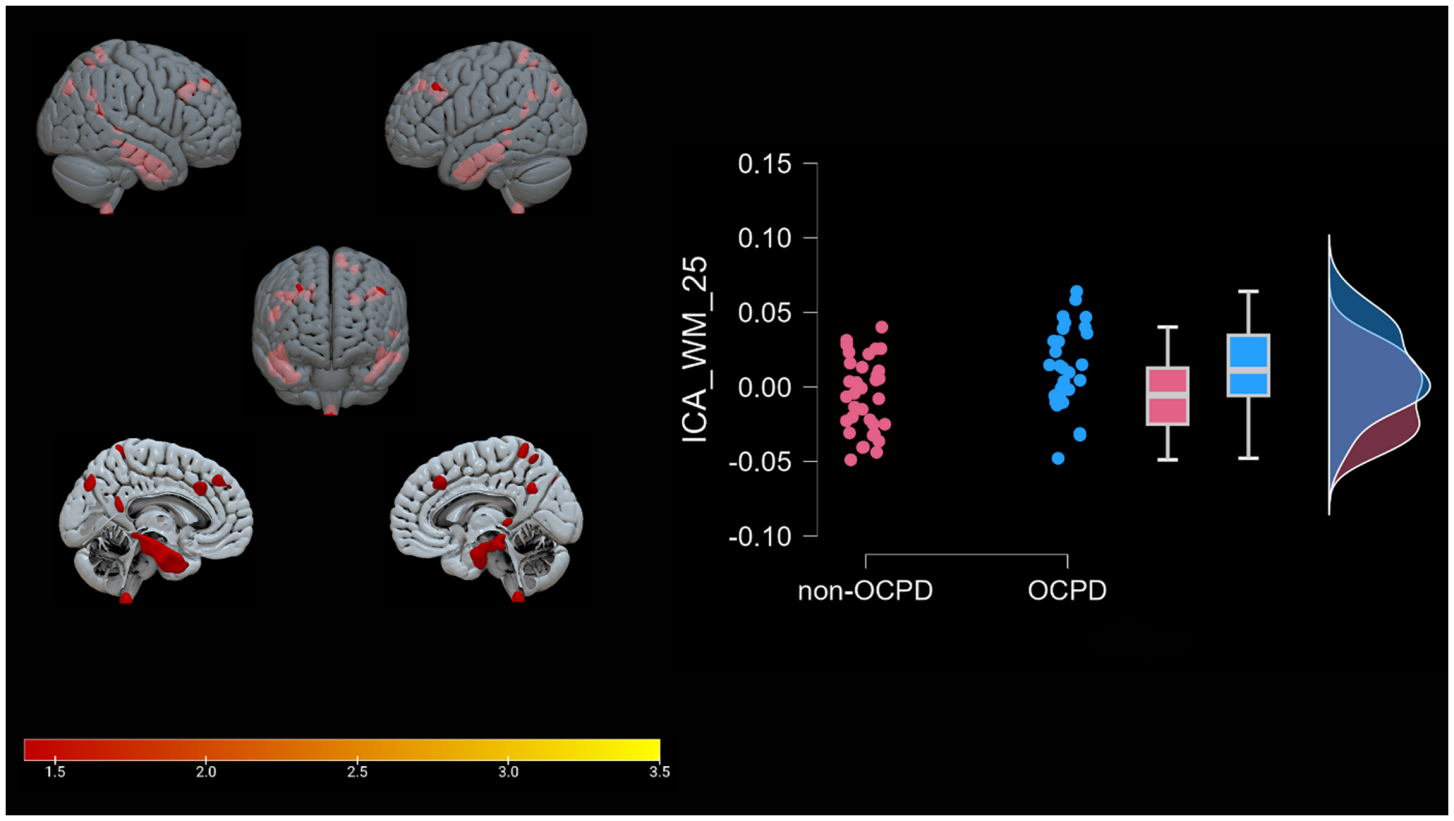
WM-25. Graphical representation of the white matter portions (mesh reconstruction) that differ between OCPD individuals vs. non-OCPD controls. OCPD individuals display higher WM density in this network compared to non-OCPD controls. A raincloud plot of the loading coefficients is also represented.

A Spearman Rank correlation analysis was conducted to examine the impact age and gender have on the construct of OCPD. The findings revealed that neither age (rho = 0.121, *p* = 0.342) nor gender (rho = −0.109, *p* = 0.392) exhibited any significant correlation with OCPD. In order to detect the components associated with OCPD, a stepwise backward regression was conducted on the loading coefficients found by the ICA. A significant winning model was found for each of the two modalities (GM: R = 0.517, R^2^ = 0.267, Adjusted R^2^ = 0.243, RMSE = 0.437; WM: R = 0.386, R^2^ = 0.149, Adjusted R^2^ = 0.121, RMSE = 0.472; for more in-depth information about the models, check the Supplementary material). The components significantly associated with OCPD were two gray matter components (GM-05: t = 3.584, *p* < 0.001 and GM-23: t = −3.091, p = 0,003) and one white matter component (WM-25: t = 2.816, *p* = 0.007). From further analysis, WM-25 did not correlate with any of the gray matter components. Interestingly, GM05 correlated significantly with both the anxiety scales used in the study. In particular, it correlated with BAI (t = 3.079; *p* = 0.003) and with the Anxiety subscale of HAD (t = 3.369, *p* = 0.001), but not the depression one. The two scales do not correlate significantly with any of the other components. GM05 component mainly included the medial frontal gyrus, superior frontal gyrus and anterior cingulate, lingual gyrus, supramarginal gyrus, insula, and precuneus (see Table 3; Figure 2). GM-23 component consisted, among the others, of portions of precuneus, fusiform gyrus, medial frontal gyrus, cingulate areas, and temporal gyrus (see Supplementary Table 3; Figure 3). Finally, WM-25 partially covered the cingulate, post central, and precentral gyrus, inferior parietal lobule, fusiform gyrus, cuneus and precuneus, and middle frontal gyrus (see Table 3; Figure 4). In the figures, warm colors represent an increase in volume compared to an average brain, with yellow indicating the greatest increase and red representing a smaller increase, similar to Figure 1.

**Table 3:** Talairach tables of the components significantly correlated with OCPD.

Area	Broadmann area	Left/right volume (cc)	Random effects max value (x, y, z)
GM-05			
Medial frontal gyrus	6, 8, 9, 10	1.5/1.8	6.4 (−3, 62, 16)/6.1 (3, 57, 5)
Sub-Gyral	6	0.8/1.2	4.1 (−30, −35, 38)/6.3 (37, 20, 20)
Superior frontal gyrus	8, 9, 10	0.9/1.0	4.9 (−3, 54, 25)/5.3 (3, 48, 31)
Superior temporal gyrus	22, 38, 42	0.9/0.6	4.6 (−67, −40, 16)/5.3 (49, 17, −7)
Middle frontal gyrus	8, 9, 10	0.5/0.6	4.3 (−21, 34, 38)/5.0 (36, 53, 21)
Anterior cingulate	10, 32	0.4/0.4	4.6 (−3, 47, 3)/4.5 (3, 47, 6)
Inferior frontal gyrus	45, 47	0.2/0.9	3.4 (−42, 15, −11)/4.5 (43, 15, −13)
Postcentral gyrus	40	0.1/0.0	3.2 (−65, −22, 18)/−999.0 (0, 0, 0)
Middle temporal gyrus	21, 39	0.1/0.1	3.1 (−65, −31, −4)/3.1 (36, −61, 27)
Supramarginal gyrus	*	0.0/0.1	−999.0 (0, 0, 0)/3.0 (50, −36, 34)
GM-23			
Culmen	*	4.5/6.7	5.6 (−1, −45, −4)/8.3 (10, −36, −15)
Fourth ventricle	*	0.2/0.2	4.0 (−1, −40, −22)/6.4 (1, −40, −19)
Cerebellar Lingual	*	0.3/0.6	5.2 (0, −44, −8)/6.1 (6, −44, −8)
Sub-Gyral	*	1.0/1.9	4.7 (−27, −46, 33)/5.7 (31, −36, 35)
Declive	*	0.3/2.0	3.5 (−9, −57, −11)/4.9 (9, −56, −11)
Culmen of vermis	*	0.2/0.0	4.6 (0, −64, −7)/−999.0 (0, 0, 0)
Precentral gyrus	*	0.1/0.1	4.1 (−34, 16, 35)/3.7 (37, 1, 28)
Fastigium	*	0.0/0.2	−999.0 (0, 0, 0)/4.0 (10, −49, −19)
Precuneus	7	0.3/0.0	4.0 (−33, −64, 36)/−999.0 (0, 0, 0)
Inferior occipital gyrus	*	0.1/0.2	3.3 (−30, −82, −5)/4.0 (36, −76, −5)
Supramarginal gyrus	*	0.0/0.1	−999.0 (0, 0, 0)/3.8 (50, −43, 34)
Medial frontal gyrus	*	0.1/0.0	3.8 (−19, 39, 19)/−999.0 (0, 0, 0)
Fusiform gyrus	37	0.1/0.3	3.7 (−36, −52, −11)/3.5 (40, −43, −13)
Cerebellar tonsil	*	0.1/0.1	3.7 (0, −51, −35)/3.4 (6, −51, −37)
Nodule	*	0.0/0.1	−999.0 (0, 0, 0)/3.5 (3, −48, −29)
Superior frontal gyrus	8	0.2/0.1	3.4 (−22, 42, 19)/3.4 (30, 30, 51)
Inferior parietal lobule	*	0.1/0.1	3.3 (−34, −52, 39)/3.4 (49, −29, 22)
Middle occipital gyrus	*	0.1/0.0	3.4 (−37, −77, 9)/−999.0 (0, 0, 0)
Cingulate gyrus	*	0.1/0.1	3.0 (−12, −44, 28)/3.3 (15, −39, 31)
Lingual gyrus	*	0.0/0.1	−999.0 (0, 0, 0)/3.3 (10, −85, −2)
Middle frontal gyrus	6	0.4/0.0	3.3 (−31, 48, 2)/−999.0 (0, 0, 0)
Uvula	*	0.0/0.1	−999.0 (0, 0, 0)/3.1 (25, −80, −23)
Insula	*	0.0/0.1	−999.0 (0, 0, 0)/3.1 (46, −31, 20)
Inferior frontal gyrus	*	0.0/0.1	−999.0 (0, 0, 0)/3.1 (37, 3, 32)
Inferior parietal lobule	40	0.2/0.0	4.4 (−45, −39, 39)/−999.0 (0, 0, 0)
Angular gyrus	*	0.1/0.0	4.3 (−40, −66, 30)/−999.0 (0, 0, 0)
Precentral gyrus	*	0.0/0.1	−999.0 (0, 0, 0)/4.2 (40, 19, 35)
Postcentral gyrus	5	0.0/0.2	−999.0 (0, 0, 0)/4.0 (39, −42, 60)
Inferior frontal gyrus	*	0.0/0.1	−999.0 (0, 0, 0)/4.0 (43, 37, 4)
Declive	*	0.2/0.1	3.7 (−45, −68, −21)/3.9 (45, −67, −22)
Pyramis	*	0.1/0.0	3.7 (−40, −66, −33)/−999.0 (0, 0, 0)
Inferior temporal gyrus	*	0.1/0.0	3.6 (−59, −12, −16)/−999.0 (0, 0, 0)
Anterior cingulate	32	0.1/0.0	3.5 (−9, 32, −7)/−999.0 (0, 0, 0)
Superior parietal lobule	7	0.0/0.1	−999.0 (0, 0, 0)/3.5 (30, −64, 54)
WM-25			
Middle temporal gyrus	19, 20, 21, 37, 39	1.7/3.3	8.2 (−58, −34, −12)/10.4 (56, −33, −14)
Inferior temporal gyrus	20, 21	0.9/1.2	6.7 (−58, −31, −15)/7.4 (56, −30, −16)
Precuneus	7	0.2/1.0	4.9 (−22, −57, 50)/6.2 (25, −54, 52)
Sub-Gyral	*	0.2/1.2	3.8 (−21, −57, 54)/6.1 (52, −33, −10)
Superior parietal lobule	7	0.4/1.0	4.2 (−24, −60, 53)/5.9 (28, −55, 44)
Middle frontal gyrus	8, 9, 10	0.7/1.1	5.6 (−42, 50, 3)/5.5 (25, 41, 37)
Medial frontal gyrus	6, 10	0.3/0.6	4.9 (−7, −12, 60)/4.7 (13, 57, −5)
Inferior parietal lobule	40	0.3/0.4	3.7 (−58, −38, 36)/4.9 (39, −48, 52)
Superior frontal gyrus	6, 8, 9	0.3/1.3	3.7 (−10, 54, 26)/4.8 (10, 24, 51)
Superior temporal gyrus	39	0.5/0.6	4.2 (−50, −32, 8)/4.8 (46, −57, 29)
Inferior frontal gyrus	9, 46	0.3/0.2	4.2 (−45, 50, 0)/3.5 (52, 12, 30)
Fusiform gyrus	*	0.0/0.1	−999.0 (0, 0, 0)/4.2 (56, −33, −19)
Middle occipital gyrus	18	0.2/0.0	4.1 (−13, −88, 15)/−999.0 (0, 0, 0)
Postcentral gyrus	3	0.1/0.1	3.9 (−59, −20, 37)/3.3 (52, −29, 40)
Parahippocampal gyrus	*	0.0/0.1	−999.0 (0, 0, 0)/3.9 (36, −16, −23)
Precentral gyrus	*	0.0/0.3	−999.0 (0, 0, 0)/3.7 (53, −11, 36)
Cuneus	18, 19	0.3/0.1	3.7 (−10, −88, 18)/3.4 (22, −81, 33)
Supramarginal gyrus	40	0.3/0.0	3.5 (−56, −42, 30)/−999.0 (0, 0, 0)
Paracentral lobule	5	0.1/0.0	3.4 (−9, −35, 54)/−999.0 (0, 0, 0)
Cingulate gyrus	32	0.1/0.0	3.3 (−12, 25, 32)/−999.0 (0, 0, 0)
Angular gyrus	*	0.0/0.1	−999.0 (0, 0, 0)/3.2 (46, −60, 32)
Inferior semi-lunar lobule	*	0.1/0.0	3.1 (−15, −69, −37)/−999.0 (0, 0, 0)

Please note that WM nomenclature is identified in terms of adjacency to GM regions. *The symbol appears for areas that are not reported to be Broadmann areas.

## Discussion

The primary goal of our study was to detect joint GM−WM differences between individuals diagnosed with OCPD and matched non-OCPD controls. To achieve these objectives, we employed, for the first time, a data fusion unsupervised ML algorithm to analyze the structural MRI images of 64 individuals (30 OCPD). This approach enabled the decomposition of the brain into independent networks characterized by the covariation of GM and WM networks. The ICA-based approach was specifically used to identify brain networks known to approximate resting-state macro-networks. This was necessary to explore the specific role of the DMN but preserving the normal individual differences in how this network may be expressed in each individual, without over imposing a predetermined mask (Scarano et al., 2024; Sorella et al., 2025). Following this, we used stepwise regression to identify the GM-WM network associated with OCPD diagnosis. The data fusion revealed 28 covarying GM-WM brain networks, but only three components (GM-05, GM-23, and WM-25) were significantly associated with OCPD. GM-05 and WM-25 were more expressed (increased matter concentration) in OCPD subjects compared to controls, while GM-23 showed the opposite trend.

**Figure 5:**
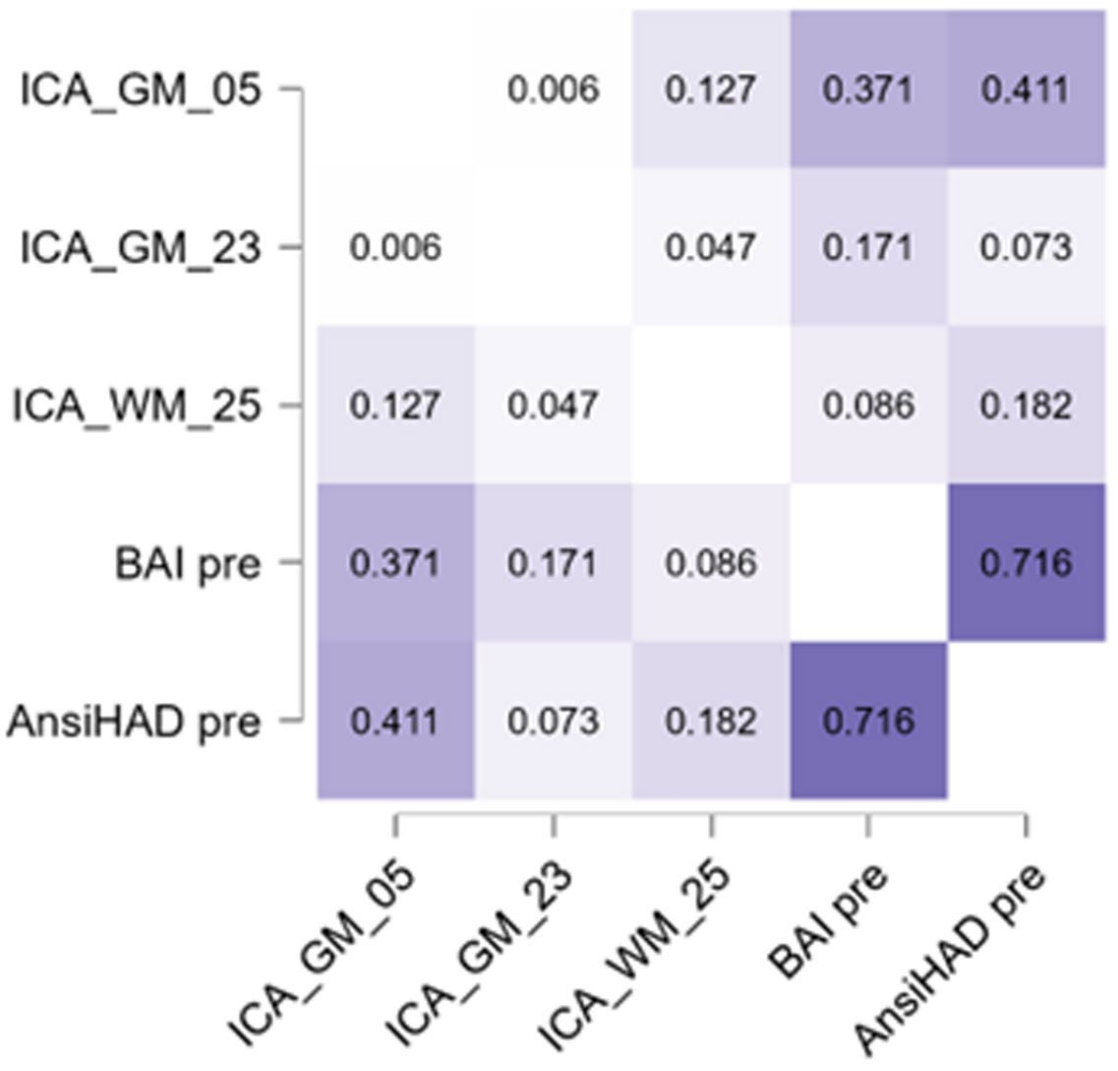
Heatmap representing the Pearson correlation values among the anxiety scales and components significant for OCPD. More information about the relation with these subscales is reported in Table 4.

GM-05 encompasses portions of the medial frontal gyrus, sub-gyral, superior frontal gyrus, superior temporal gyrus, middle frontal gyrus, anterior cingulate and inferior frontal gyrus, post central gyrus, middle temporal gyrus, and supramarginal gyrus. All these areas exhibited higher density in the OCPD group relative to controls. Previous studies have identified various alterations in some of the regions comprising GM05 in OCPD: both the superior and medial frontal gyri have shown increased activity fluctuation in individuals with OCPD (Lei et al., 2020), while other studies underline the role of cingulate cortex alterations in explaining some OCPD’s typical traits, such as harm avoidance and anxiety-related traits (Tuominen et al., 2012; Colic et al., 2018). Additionally, our analysis revealed a correlation between GM05 and two of the three anxiety subscales (BAI, r = 0.371, *p* = 0.003; HAD, r = 0.411, *p* < 0.001). This correlation aligns with the differences observed between OCPD and control groups in anxiety scores and can be interpreted as evidence of the influence that alterations in GM05 regions may have on the anxious traits characteristic of OCPD. Notably, this hypothesis is supported by the absence of any significant correlation between GM05 and the diagnosis of the other two Cluster C personality disorders assessed in this study. For further details, see Figure 5.

GM-23 includes cerebellar regions (such as the Culmen, cerebellar Tonsil, Nodule, uvula, declive, and pyramis), as well as the sub-gyral area, precentral gyrus, precuneus, occipital gyrus, supramarginal gyrus, fusiform gyrus, superior frontal gyrus, inferior parietal lobule, middle occipital gyrus, cingulate gyrus, lingual gyrus, middle frontal gyrus, insula, inferior frontal gyrus, angular gyrus, precentral and postcentral gyrus, inferior temporal gyrus, anterior cingulate cortex, and superior parietal lobule. Of particular interest is the presence of the precuneus in this component, as this area is associated with significant traits of Obsessive-Compulsive Personality Disorder (OCPD), such as perfectionism and rumination (Coutinho et al., 2016). Alterations in gray matter density in regions such as the culmen, cerebellar lingual, cerebellar tonsil, and declive suggest an involvement of the cerebellum in individuals with OCPD. This hypothesis is further supported by previous studies (Laricchiuta et al., 2014; Petrosini et al., 2017) that link cerebellar volumes to personality traits relevant to OCPD, such as novelty seeking and harm avoidance. Specifically, novelty seeking is positively correlated with cerebellar volumes, while harm avoidance is negatively correlated with the same measure. The presence of areas associated with these traits in the reported significant components highlights a possible unique relationship between OCPD and harm avoidance. This connection is supported by previous studies (Lee and Wu, 2019; Ettelt et al., 2008; Samuels et al., 2000), which suggest a link between this trait and both OCPD and OCD. In contrast, regarding novelty seeking, no other relevant studies, to our knowledge, have identified a specific relationship with OCPD. Furthermore, this study does not directly investigate novelty seeking, so the reported results are insufficient to support a distinct connection between the two constructs. The component GM-23 also includes portions of insula, an area associated with characteristics altered in OCD and OCPD, such as empathy, cognitive flexibility (Gogolla, 2017), and that has been found to be altered in precedent studies investigating OCPD (Lei et al., 2020). Most importantly, including portions of insula and anterior cingulate cortex, the component appears to be related to the Salience Network (Seeley, 2019). The Salience Network is an important brain network that functions as a dynamic switch between concentration on self (mediated by DMN) and task-related and directed attention (Schimmelpfennig et al., 2023). Its involvement in OCPD may indicate a difficulty in the dynamic switching process among individuals with the condition.

Lastly, the WM-25 positive component consists of white matter regions adjacent to the middle temporal gyrus, inferior temporal gyrus, precuneus, sub-gyral area, superior parietal lobule, middle frontal gyrus, medial frontal gyrus, inferior parietal lobule, fusiform gyrus, middle occipital gyrus, postcentral and precentral gyrus, parahippocampal gyrus, cuneus, supramarginal gyrus, paracentral lobule, cingulate gyrus, angular gyrus, and inferior semi-lunar lobule. Increased white matter density in the precuneus has been previously observed in a resting-state functional MRI study (Coutinho et al., 2016), where the increase was linked to self-reflection, as well as accessing past events for problem-solving and future planning. While our study does not explicitly measure these constructs, our results further support the involvement of precuneus connectivity in OCPD. Interestingly, the WM-25 network also includes increased WM volume in frontal areas, such as the medial and middle frontal gyrus, coherently with what we observed for GM-05. The presence of parahippocampal gyrus suggests a limbic alteration in subjects with OCPD, supporting the results of previous studies that link this system to OCPD (Kyeong et al., 2014; Millon and Davis, 1996). The relationship between these three components and the psychological scales used for the assessment are reported in Table 4.

**Table 4:** Association within the significant components for OCPD and psychological scales.

Variable	GM-05		GM-23		WM-25	
	Pearson’s r	*p*-value	Pearson’s r	*p*-value	Pearson’s r	*p*-value
HARS	−0.043	0.743	0.107	0.399	−0.007	0.959
HAD anxiety	0.411	<0.01	0.073	0.564	0.182	0.151
HAD depression	0.244	0.052	0.044	0.729	0.031	0.809
EAG	−0.227	0.072	0.006	0.954	−0.045	0.725
S-R	0.136	0.285	0.254	0.051	0.005	0.972
BAI	0.371	0.003	0.171	0.177	0.086	0.497
IPDE OCPD	0.381	0.002	−0.286	0.022	0.364	0.003

As expected, the resulting networks overlap with parts of the Default Mode Network (DMN). The Default Mode Network is the name given to a network of distributed and interconnected brain areas that are typically suppressed when an individual is focused on external stimuli and are, instead, mainly activated for internally focused thought processes (Menon, 2023), such as self-referential processing, future planning, cues evaluation and emotional regulation (Raichle, 2015). The network includes the medial prefrontal cortex, posterior cingulate cortex with the adjacent precuneus, the bilateral inferior parietal cortex, and the medial temporal cortex (Buckner et al., 2008; Fox and Raichle, 2007; Raichle, 2015). The link between DMN and personality disorders is well documented in literature (see, for example, Coutinho et al., 2016, for OCPD; Yang et al., 2016, Langerbeck et al., 2023 for borderline personality disorder, and Zhang et al., 2014 for Schizotypal personality disorder). Thus, a result that includes it was expected. Regarding OCPD specifically, the DMN is responsible for several psychological processes relevant to its pathophysiology (Coutinho et al., 2016), including reflective self-awareness, introspection (Buckner and Carroll, 2007), retrospective memory, and prospective thinking (Delamillieure et al., 2010). The propensity for self-referential thoughts, rethinking recent past events, and future planning are all processes dependent on the DMN and are altered in OCPD. The involvement of the DMN in symptoms and the development of anxious states has been observed previously in Obsessive-Compulsive Disorder (Gonçalves et al., 2017). Our study further extends previous evidence supporting the hypothesis of DMN involvement in OCPD. Of the areas showing increased volume in OCPD patients, GM-5 includes the medial frontal gyrus, superior temporal gyrus, anterior cingulate, and inferior frontal gyrus. The medial frontal gyrus and superior temporal gyrus have been linked to the DMN (Menon, 2023; Uddin et al., 2009). The anterior cingulate is not only considered part of the DMN (Buckner and DiNicola, 2019) but is also recognized as a key component of the Salience Network (Seeley et al., 2007), which is involved in DMN suppression. The presence of the insula and anterior cingulate cortex in GM-23 further supports the involvement of the Salience Network. Finally, the inferior frontal gyrus is directly involved in the network (Menon, 2023; Uddin et al., 2009). GM-23 includes the precuneus, an area that is considered a part of the DMN; WM-25 confirm a white matter involvement adjacent to the precuneus, the medial frontal gyrus and inferior temporal gyrus, already discussed for the gray matter component were included, but also includes cingulate gyrus and inferior parietal lobule, two areas that are part of DMN (Buckner and DiNicola, 2019). In conclusion, GM-05 and WM-25 are two components that include portions of DMN associated with OCPD. In contrast, GM-23 includes mainly areas (except for Precuneus) that have never been reported to be related to DMN. This result can be interpreted in the direction of an increase of Default Mode Network volume for patients with OCPD, while GM-23, which is little if not associated with DMN, is more expressed in controls; thus, a volume decrement in areas included in GM-23 is associated with OCPD, while for what concerns GM-05 and WM-25 (the circuits that include areas associated with DMN) is instead an increment in volume to be related to OCPD.

Of note, GM-05 and GM-23 do not correlate with WM-25. This means that although OCPD is characterized by alterations in both modalities (GM and WM), these networks are not joint.

These results could enhance our understanding of OCPD traits, particularly in relation to the similar disorder OCD, briefly discussed in this study. OCPD has been associated with emotional overcontrol and reduced affective expression, potentially linked to altered connectivity in the insula and amygdala (Grant et al., 2012).

Interestingly, the present study did not find evidence of striatal or orbitofrontal cortex involvement in OCPD among the participants. Instead, alterations in the ACC, but not in the dorsal prefrontal network, were observed. This finding partially aligns with existing literature while also suggesting the need for a more in-depth investigation into these regions, focusing on their functional roles in OCPD.

Moreover, a correlation emerged between GM-05, a component associated with OCPD, and anxiety subscales. This could be interpreted as preliminary evidence of a relationship between OCPD and anxiety, possibly hinting at a link between OCPD and OCD. However, this study does not provide sufficient evidence to support such a hypothesis fully, underscoring the need for further research.

Despite its merits, this study is not without limitations. First, while the sample size is comparable to or larger than that of some previous studies, it remains relatively modest. Increasing the sample size would enhance the robustness of these findings and allow for more nuanced subgroup analyses. Second, although control subjects were selected based on their IPDE-OCPD scores, other clinical dimensions were not assessed in the control group. Future studies may want to use multiple assessment tools or conduct a more thorough investigation of comorbidities. Moreover, our study exclusively focused on structural MRI data, specifically examining gray and white matter concentrations. Although this approach provided valuable insights into the structural abnormalities associated with OCPD, it does not address the functional aspects of brain activity. Incorporating functional neuroimaging data, such as resting-state or task-based fMRI, could offer a more comprehensive understanding of OCPD neural mechanisms by functional connectivity patterns and dynamic brain activity related to its typical behaviors. While we identified specific brain regions and networks associated with OCPD, it remains unclear whether these findings are specific to this personality disorder or extend to other personalities, especially cluster C. Comparative studies involving different types of personalities could help clarify the specificity and shared neural mechanisms underlying these disorders. A similar reflection could be drawn regarding OCD; as it has been explained before, OCD and OCPD share some common characteristics. We suggest that further studies directly compare the two disorders to further and directly underline the similarities and the differences between the two disorders. In our study, we did not find significant gender differences in the OCPD group, despite past epidemiological findings suggesting gender imbalances. However, more recent evidence from a comprehensive meta-analysis and meta-regression of the global prevalence of obsessive-compulsive personality disorder (Clemente et al., 2022) found no significant gender-related effect. This challenges the notion that gender differences are a clear and consistent factor in OCPD, particularly at the neural level. Moreover, the relatively small sample size of our study may have limited our ability to detect subtle neural differences between genders. A larger sample may be needed to capture small but meaningful variations.

Finally, although the approach we proposed in this study is based on Parallel ICA, other data fusion approaches could be implemented (in the form, for example, of Joint ICA or tIVA).

## Conclusion

This study aimed to understand better the GM and WM alterations in individuals diagnosed with OCPD. In particular, we tested the hypothesis that OCPD’s regions ascribed to the DMN were altered. We used Parallel ICA, a data fusion machine learning method, to extract GM and WM networks that approximate macro-resting state networks. Results indicate a contribution of many brain regions overlapping with the DMN. These results may pave the way for future OCPD biomarkers and neurostimulation methods to target dysfunctional brain regions.

Disruptions in the DMN can affect how emotions are processed and regulated, potentially interfering with the therapeutic mechanisms of interventions like Cognitive-Behavior Therapy. For instance, when the DMN is hyperactive or dysregulated, it may impair the cognitive flexibility necessary for effective emotion regulation, which could undermine the success of interventions focused on emotional processing. These findings highlight the importance of the DMN in emotional regulation and have significant implications for the development of biomarkers for disorders such as OCPD. Furthermore, they suggest potential pathways for neurostimulation approaches aimed at modulating dysfunctional brain regions. By targeting these disrupted areas, it may be possible to enhance the effectiveness of existing therapies and open new avenues for treating psychological conditions associated with maladaptive brain network activity.

## Data Availability

The raw data supporting the conclusions of this article will be made available by the authors, without undue reservation.

## Footnotes

^1^ http://www.neuro.uni-jena.de/cat/

^2^ https://www.fil.ion.ucl.ac.uk/spm/software

^3^ http://mialab.mrn.org/software/fit

^4^ https://it.mathworks.com/products/matlab.html

^5^ https://www.nitrc.org/projects/surfice/
