# Supplementary material for: Gray and white matter alterations in Obsessive-Compulsive Personality Disorder: a data fusion machine learning approach
Source: Front Hum Neurosci. 2025 May 14;19:1559760. doi: 10.3389/fnhum.2025.1559760 (PMC12116498; doi:10.3389/fnhum.2025.1559760)
Supplement: Supplementary file 1 [file Supplementary_file_1.docx]

**Grey and White Matter alterations in Obsessive-Compulsive Personality disorder: a Data Fusion Machine Learning approach**

**Supplementary material**

Lorenzo Arena^1^, Wenceslao Peñate^2,3^, Francisco Rivero^3^, Rosario J. Marrero^2,3^, Teresa Olivares^2^, Alessandro Scarano^1^, Ascensión Fumero^2,3^ , Alessandro Grecucci^1^

1 Department of Psychology and Cognitive Science, University of Trento, TN, Italy

2 Departamento de Psicología Clínica, Psicobiología y Metodología, Facultad de Psicología, Universidad de La Laguna, 38200 La Laguna, Tenerife, Spain

3 Instituto Universitario de Neurociencia (IUNE), Universidad de La Laguna, 38200 La Laguna, Tenerife, Spain.

4 Facultad de Ciencias de la Salud, Universidad Europea de Canarias

**Linear Regression model details**

**GM**

| Model Summary - OCPD dia | | | | | | | | | |
| --- | --- | --- | --- | --- | --- | --- | --- | --- | --- |
| Model | | R | | R² | | Adjusted R² | | RMSE | |
| 1 |  | 0.639 |  | 0.408 |  | -0.065 |  | 0.519 |  |
| 2 |  | 0.639 |  | 0.408 |  | -0.036 |  | 0.512 |  |
| 3 |  | 0.639 |  | 0.408 |  | -0.008 |  | 0.505 |  |
| 4 |  | 0.639 |  | 0.408 |  | 0.019 |  | 0.498 |  |
| 5 |  | 0.639 |  | 0.408 |  | 0.043 |  | 0.492 |  |
| 6 |  | 0.638 |  | 0.407 |  | 0.067 |  | 0.486 |  |
| 7 |  | 0.637 |  | 0.406 |  | 0.087 |  | 0.481 |  |
| 8 |  | 0.635 |  | 0.404 |  | 0.105 |  | 0.476 |  |
| 9 |  | 0.634 |  | 0.402 |  | 0.123 |  | 0.471 |  |
| 10 |  | 0.632 |  | 0.399 |  | 0.139 |  | 0.467 |  |
| 11 |  | 0.630 |  | 0.397 |  | 0.156 |  | 0.462 |  |
| 12 |  | 0.628 |  | 0.395 |  | 0.171 |  | 0.458 |  |
| 13 |  | 0.626 |  | 0.392 |  | 0.185 |  | 0.454 |  |
| 14 |  | 0.623 |  | 0.388 |  | 0.196 |  | 0.451 |  |
| 15 |  | 0.620 |  | 0.384 |  | 0.208 |  | 0.448 |  |
| 16 |  | 0.618 |  | 0.382 |  | 0.221 |  | 0.444 |  |
| 17 |  | 0.615 |  | 0.379 |  | 0.232 |  | 0.441 |  |
| 18 |  | 0.612 |  | 0.375 |  | 0.242 |  | 0.438 |  |
| 19 |  | 0.608 |  | 0.369 |  | 0.250 |  | 0.436 |  |
| 20 |  | 0.602 |  | 0.363 |  | 0.257 |  | 0.434 |  |
| 21 |  | 0.596 |  | 0.355 |  | 0.261 |  | 0.432 |  |
| 22 |  | 0.588 |  | 0.346 |  | 0.264 |  | 0.431 |  |
| 23 |  | 0.579 |  | 0.335 |  | 0.265 |  | 0.431 |  |
| 24 |  | 0.567 |  | 0.322 |  | 0.263 |  | 0.432 |  |
| 25 |  | 0.555 |  | 0.308 |  | 0.261 |  | 0.432 |  |
| 26 |  | 0.541 |  | 0.293 |  | 0.258 |  | 0.433 |  |
| 27 |  | 0.517 |  | 0.267 |  | 0.243 |  | 0.437 |  |
|  | | | | | | | | | |

| ANOVA | | | | | | | | | | | | | |
| --- | --- | --- | --- | --- | --- | --- | --- | --- | --- | --- | --- | --- | --- |
| Model | |  | | Sum of Squares | | df | | Mean Square | | F | | p | |
| 1 |  | Regression |  | 6.507 |  | 28 |  | 0.232 |  | 0.863 |  | 0.653 |  |
|  |  | Residual |  | 9.430 |  | 35 |  | 0.269 |  |  |  |  |  |
|  |  | Total |  | 15.938 |  | 63 |  |  |  |  |  |  |  |
| 2 |  | Regression |  | 6.507 |  | 27 |  | 0.241 |  | 0.920 |  | 0.584 |  |
|  |  | Residual |  | 9.431 |  | 36 |  | 0.262 |  |  |  |  |  |
|  |  | Total |  | 15.938 |  | 63 |  |  |  |  |  |  |  |
| 3 |  | Regression |  | 6.506 |  | 26 |  | 0.250 |  | 0.982 |  | 0.512 |  |
|  |  | Residual |  | 9.432 |  | 37 |  | 0.255 |  |  |  |  |  |
|  |  | Total |  | 15.938 |  | 63 |  |  |  |  |  |  |  |
| 4 |  | Regression |  | 6.502 |  | 25 |  | 0.260 |  | 1.048 |  | 0.440 |  |
|  |  | Residual |  | 9.435 |  | 38 |  | 0.248 |  |  |  |  |  |
|  |  | Total |  | 15.938 |  | 63 |  |  |  |  |  |  |  |
| 5 |  | Regression |  | 6.498 |  | 24 |  | 0.271 |  | 1.119 |  | 0.369 |  |
|  |  | Residual |  | 9.440 |  | 39 |  | 0.242 |  |  |  |  |  |
|  |  | Total |  | 15.938 |  | 63 |  |  |  |  |  |  |  |
| 6 |  | Regression |  | 6.491 |  | 23 |  | 0.282 |  | 1.195 |  | 0.303 |  |
|  |  | Residual |  | 9.446 |  | 40 |  | 0.236 |  |  |  |  |  |
|  |  | Total |  | 15.937 |  | 63 |  |  |  |  |  |  |  |
| 7 |  | Regression |  | 6.464 |  | 22 |  | 0.294 |  | 1.271 |  | 0.248 |  |
|  |  | Residual |  | 9.474 |  | 41 |  | 0.231 |  |  |  |  |  |
|  |  | Total |  | 15.938 |  | 63 |  |  |  |  |  |  |  |
| 8 |  | Regression |  | 6.433 |  | 21 |  | 0.306 |  | 1.354 |  | 0.198 |  |
|  |  | Residual |  | 9.505 |  | 42 |  | 0.226 |  |  |  |  |  |
|  |  | Total |  | 15.938 |  | 63 |  |  |  |  |  |  |  |
| 9 |  | Regression |  | 6.402 |  | 20 |  | 0.320 |  | 1.444 |  | 0.155 |  |
|  |  | Residual |  | 9.535 |  | 43 |  | 0.222 |  |  |  |  |  |
|  |  | Total |  | 15.938 |  | 63 |  |  |  |  |  |  |  |
| 10 |  | Regression |  | 6.359 |  | 19 |  | 0.335 |  | 1.537 |  | 0.119 |  |
|  |  | Residual |  | 9.579 |  | 44 |  | 0.218 |  |  |  |  |  |
|  |  | Total |  | 15.937 |  | 63 |  |  |  |  |  |  |  |
| 11 |  | Regression |  | 6.334 |  | 18 |  | 0.352 |  | 1.649 |  | 0.088 |  |
|  |  | Residual |  | 9.603 |  | 45 |  | 0.213 |  |  |  |  |  |
|  |  | Total |  | 15.938 |  | 63 |  |  |  |  |  |  |  |
| 12 |  | Regression |  | 6.289 |  | 17 |  | 0.370 |  | 1.764 |  | 0.064 |  |
|  |  | Residual |  | 9.648 |  | 46 |  | 0.210 |  |  |  |  |  |
|  |  | Total |  | 15.938 |  | 63 |  |  |  |  |  |  |  |
| 13 |  | Regression |  | 6.243 |  | 16 |  | 0.390 |  | 1.892 |  | 0.046 |  |
|  |  | Residual |  | 9.694 |  | 47 |  | 0.206 |  |  |  |  |  |
|  |  | Total |  | 15.938 |  | 63 |  |  |  |  |  |  |  |
| 14 |  | Regression |  | 6.177 |  | 15 |  | 0.412 |  | 2.025 |  | 0.033 |  |
|  |  | Residual |  | 9.760 |  | 48 |  | 0.203 |  |  |  |  |  |
|  |  | Total |  | 15.938 |  | 63 |  |  |  |  |  |  |  |
| 15 |  | Regression |  | 6.124 |  | 14 |  | 0.437 |  | 2.184 |  | 0.022 |  |
|  |  | Residual |  | 9.814 |  | 49 |  | 0.200 |  |  |  |  |  |
|  |  | Total |  | 15.937 |  | 63 |  |  |  |  |  |  |  |
| 16 |  | Regression |  | 6.086 |  | 13 |  | 0.468 |  | 2.376 |  | 0.014 |  |
|  |  | Residual |  | 9.852 |  | 50 |  | 0.197 |  |  |  |  |  |
|  |  | Total |  | 15.937 |  | 63 |  |  |  |  |  |  |  |
| 17 |  | Regression |  | 6.034 |  | 12 |  | 0.503 |  | 2.589 |  | 0.009 |  |
|  |  | Residual |  | 9.904 |  | 51 |  | 0.194 |  |  |  |  |  |
|  |  | Total |  | 15.938 |  | 63 |  |  |  |  |  |  |  |
| 18 |  | Regression |  | 5.973 |  | 11 |  | 0.543 |  | 2.833 |  | 0.006 |  |
|  |  | Residual |  | 9.965 |  | 52 |  | 0.192 |  |  |  |  |  |
|  |  | Total |  | 15.938 |  | 63 |  |  |  |  |  |  |  |
| 19 |  | Regression |  | 5.883 |  | 10 |  | 0.588 |  | 3.101 |  | 0.004 |  |
|  |  | Residual |  | 10.055 |  | 53 |  | 0.190 |  |  |  |  |  |
|  |  | Total |  | 15.937 |  | 63 |  |  |  |  |  |  |  |
| 20 |  | Regression |  | 5.782 |  | 9 |  | 0.642 |  | 3.416 |  | 0.002 |  |
|  |  | Residual |  | 10.155 |  | 54 |  | 0.188 |  |  |  |  |  |
|  |  | Total |  | 15.937 |  | 63 |  |  |  |  |  |  |  |
| 21 |  | Regression |  | 5.658 |  | 8 |  | 0.707 |  | 3.784 |  | 0.001 |  |
|  |  | Residual |  | 10.280 |  | 55 |  | 0.187 |  |  |  |  |  |
|  |  | Total |  | 15.938 |  | 63 |  |  |  |  |  |  |  |
| 22 |  | Regression |  | 5.514 |  | 7 |  | 0.788 |  | 4.232 |  | < .001 |  |
|  |  | Residual |  | 10.423 |  | 56 |  | 0.186 |  |  |  |  |  |
|  |  | Total |  | 15.938 |  | 63 |  |  |  |  |  |  |  |
| 23 |  | Regression |  | 5.337 |  | 6 |  | 0.890 |  | 4.783 |  | < .001 |  |
|  |  | Residual |  | 10.600 |  | 57 |  | 0.186 |  |  |  |  |  |
|  |  | Total |  | 15.937 |  | 63 |  |  |  |  |  |  |  |
| 24 |  | Regression |  | 5.125 |  | 5 |  | 1.025 |  | 5.499 |  | < .001 |  |
|  |  | Residual |  | 10.812 |  | 58 |  | 0.186 |  |  |  |  |  |
|  |  | Total |  | 15.937 |  | 63 |  |  |  |  |  |  |  |
| 25 |  | Regression |  | 4.906 |  | 4 |  | 1.226 |  | 6.559 |  | < .001 |  |
|  |  | Residual |  | 11.032 |  | 59 |  | 0.187 |  |  |  |  |  |
|  |  | Total |  | 15.937 |  | 63 |  |  |  |  |  |  |  |
| 26 |  | Regression |  | 4.672 |  | 3 |  | 1.557 |  | 8.294 |  | < .001 |  |
|  |  | Residual |  | 11.266 |  | 60 |  | 0.188 |  |  |  |  |  |
|  |  | Total |  | 15.937 |  | 63 |  |  |  |  |  |  |  |
| 27 |  | Regression |  | 4.262 |  | 2 |  | 2.131 |  | 11.133 |  | < .001 |  |
|  |  | Residual |  | 11.676 |  | 61 |  | 0.191 |  |  |  |  |  |
|  |  | Total |  | 15.937 |  | 63 |  |  |  |  |  |  |  |
|  | | | | | | | | | | | | | |

| Coefficients | | | | | | | | | | | | | |
| --- | --- | --- | --- | --- | --- | --- | --- | --- | --- | --- | --- | --- | --- |
| Model | |  | | Unstandardized | | Standard Error | | Standardized | | t | | p | |
| 1 |  | (Intercept) |  | 26.957 |  | 49.905 |  |  |  | 0.540 |  | 0.593 |  |
|  |  | ICA_GM_01 |  | 2.024 |  | 2.493 |  | 0.141 |  | 0.812 |  | 0.422 |  |
|  |  | ICA_GM_02 |  | -3.092 |  | 6.408 |  | -0.228 |  | -0.483 |  | 0.632 |  |
|  |  | ICA_GM_03 |  | 25.165 |  | 47.905 |  | 0.546 |  | 0.525 |  | 0.603 |  |
|  |  | ICA_GM_04 |  | -2.577 |  | 3.022 |  | -0.167 |  | -0.853 |  | 0.400 |  |
|  |  | ICA_GM_05 |  | 4.030 |  | 4.855 |  | 0.234 |  | 0.830 |  | 0.412 |  |
|  |  | ICA_GM_06 |  | 0.304 |  | 2.934 |  | 0.017 |  | 0.104 |  | 0.918 |  |
|  |  | ICA_GM_07 |  | -0.530 |  | 2.644 |  | -0.029 |  | -0.201 |  | 0.842 |  |
|  |  | ICA_GM_08 |  | 1.557 |  | 2.526 |  | 0.087 |  | 0.616 |  | 0.542 |  |
|  |  | ICA_GM_09 |  | 1.033 |  | 2.611 |  | 0.054 |  | 0.396 |  | 0.695 |  |
|  |  | ICA_GM_10 |  | -2.623 |  | 3.187 |  | -0.151 |  | -0.823 |  | 0.416 |  |
|  |  | ICA_GM_11 |  | -0.166 |  | 2.651 |  | -0.009 |  | -0.063 |  | 0.950 |  |
|  |  | ICA_GM_12 |  | -0.073 |  | 1.948 |  | -0.005 |  | -0.038 |  | 0.970 |  |
|  |  | ICA_GM_13 |  | -0.800 |  | 5.104 |  | -0.044 |  | -0.157 |  | 0.876 |  |
|  |  | ICA_GM_14 |  | 2.295 |  | 2.545 |  | 0.121 |  | 0.902 |  | 0.373 |  |
|  |  | ICA_GM_15 |  | 3.308 |  | 8.358 |  | 0.187 |  | 0.396 |  | 0.695 |  |
|  |  | ICA_GM_16 |  | -2.700 |  | 3.892 |  | -0.151 |  | -0.694 |  | 0.492 |  |
|  |  | ICA_GM_17 |  | -2.082 |  | 4.524 |  | -0.105 |  | -0.460 |  | 0.648 |  |
|  |  | ICA_GM_18 |  | 0.879 |  | 3.176 |  | 0.050 |  | 0.277 |  | 0.784 |  |
|  |  | ICA_GM_19 |  | -4.332 |  | 4.255 |  | -0.236 |  | -1.018 |  | 0.316 |  |
|  |  | ICA_GM_20 |  | -1.848 |  | 3.241 |  | -0.098 |  | -0.570 |  | 0.572 |  |
|  |  | ICA_GM_21 |  | 2.586 |  | 4.084 |  | 0.138 |  | 0.633 |  | 0.531 |  |
|  |  | ICA_GM_22 |  | -1.214 |  | 3.401 |  | -0.074 |  | -0.357 |  | 0.723 |  |
|  |  | ICA_GM_23 |  | -9.364 |  | 6.942 |  | -0.553 |  | -1.349 |  | 0.186 |  |
|  |  | ICA_GM_24 |  | 0.779 |  | 3.393 |  | 0.040 |  | 0.230 |  | 0.820 |  |
|  |  | ICA_GM_25 |  | -3.040 |  | 4.347 |  | -0.155 |  | -0.699 |  | 0.489 |  |
|  |  | ICA_GM_26 |  | -0.804 |  | 2.555 |  | -0.043 |  | -0.315 |  | 0.755 |  |
|  |  | ICA_GM_27 |  | 0.940 |  | 2.248 |  | 0.059 |  | 0.418 |  | 0.679 |  |
|  |  | ICA_GM_28 |  | -2.111 |  | 2.574 |  | -0.113 |  | -0.820 |  | 0.418 |  |
| 2 |  | (Intercept) |  | 26.759 |  | 48.935 |  |  |  | 0.547 |  | 0.588 |  |
|  |  | ICA_GM_01 |  | 2.015 |  | 2.446 |  | 0.140 |  | 0.824 |  | 0.415 |  |
|  |  | ICA_GM_02 |  | -3.066 |  | 6.280 |  | -0.226 |  | -0.488 |  | 0.628 |  |
|  |  | ICA_GM_03 |  | 24.975 |  | 46.973 |  | 0.542 |  | 0.532 |  | 0.598 |  |
|  |  | ICA_GM_04 |  | -2.560 |  | 2.950 |  | -0.166 |  | -0.868 |  | 0.391 |  |
|  |  | ICA_GM_05 |  | 4.052 |  | 4.751 |  | 0.236 |  | 0.853 |  | 0.399 |  |
|  |  | ICA_GM_06 |  | 0.301 |  | 2.892 |  | 0.017 |  | 0.104 |  | 0.918 |  |
|  |  | ICA_GM_07 |  | -0.526 |  | 2.604 |  | -0.029 |  | -0.202 |  | 0.841 |  |
|  |  | ICA_GM_08 |  | 1.550 |  | 2.485 |  | 0.086 |  | 0.624 |  | 0.537 |  |
|  |  | ICA_GM_09 |  | 1.036 |  | 2.573 |  | 0.055 |  | 0.403 |  | 0.690 |  |
|  |  | ICA_GM_10 |  | -2.611 |  | 3.127 |  | -0.150 |  | -0.835 |  | 0.409 |  |
|  |  | ICA_GM_11 |  | -0.158 |  | 2.604 |  | -0.008 |  | -0.061 |  | 0.952 |  |
|  |  | ICA_GM_13 |  | -0.782 |  | 5.011 |  | -0.043 |  | -0.156 |  | 0.877 |  |
|  |  | ICA_GM_14 |  | 2.298 |  | 2.509 |  | 0.121 |  | 0.916 |  | 0.366 |  |
|  |  | ICA_GM_15 |  | 3.274 |  | 8.193 |  | 0.185 |  | 0.400 |  | 0.692 |  |
|  |  | ICA_GM_16 |  | -2.689 |  | 3.826 |  | -0.150 |  | -0.703 |  | 0.487 |  |
|  |  | ICA_GM_17 |  | -2.070 |  | 4.450 |  | -0.104 |  | -0.465 |  | 0.645 |  |
|  |  | ICA_GM_18 |  | 0.882 |  | 3.130 |  | 0.050 |  | 0.282 |  | 0.780 |  |
|  |  | ICA_GM_19 |  | -4.317 |  | 4.178 |  | -0.235 |  | -1.033 |  | 0.308 |  |
|  |  | ICA_GM_20 |  | -1.854 |  | 3.191 |  | -0.098 |  | -0.581 |  | 0.565 |  |
|  |  | ICA_GM_21 |  | 2.580 |  | 4.025 |  | 0.138 |  | 0.641 |  | 0.526 |  |
|  |  | ICA_GM_22 |  | -1.200 |  | 3.333 |  | -0.073 |  | -0.360 |  | 0.721 |  |
|  |  | ICA_GM_23 |  | -9.341 |  | 6.818 |  | -0.552 |  | -1.370 |  | 0.179 |  |
|  |  | ICA_GM_24 |  | 0.781 |  | 3.346 |  | 0.040 |  | 0.233 |  | 0.817 |  |
|  |  | ICA_GM_25 |  | -3.022 |  | 4.262 |  | -0.155 |  | -0.709 |  | 0.483 |  |
|  |  | ICA_GM_26 |  | -0.802 |  | 2.519 |  | -0.043 |  | -0.319 |  | 0.752 |  |
|  |  | ICA_GM_27 |  | 0.945 |  | 2.213 |  | 0.059 |  | 0.427 |  | 0.672 |  |
|  |  | ICA_GM_28 |  | -2.111 |  | 2.538 |  | -0.113 |  | -0.832 |  | 0.411 |  |
| 3 |  | (Intercept) |  | 27.514 |  | 46.675 |  |  |  | 0.589 |  | 0.559 |  |
|  |  | ICA_GM_01 |  | 2.044 |  | 2.365 |  | 0.142 |  | 0.864 |  | 0.393 |  |
|  |  | ICA_GM_02 |  | -3.149 |  | 6.045 |  | -0.232 |  | -0.521 |  | 0.606 |  |
|  |  | ICA_GM_03 |  | 25.705 |  | 44.779 |  | 0.558 |  | 0.574 |  | 0.569 |  |
|  |  | ICA_GM_04 |  | -2.592 |  | 2.864 |  | -0.168 |  | -0.905 |  | 0.371 |  |
|  |  | ICA_GM_05 |  | 4.000 |  | 4.607 |  | 0.233 |  | 0.868 |  | 0.391 |  |
|  |  | ICA_GM_06 |  | 0.324 |  | 2.828 |  | 0.018 |  | 0.115 |  | 0.909 |  |
|  |  | ICA_GM_07 |  | -0.540 |  | 2.558 |  | -0.029 |  | -0.211 |  | 0.834 |  |
|  |  | ICA_GM_08 |  | 1.559 |  | 2.447 |  | 0.087 |  | 0.637 |  | 0.528 |  |
|  |  | ICA_GM_09 |  | 1.036 |  | 2.538 |  | 0.055 |  | 0.408 |  | 0.685 |  |
|  |  | ICA_GM_10 |  | -2.640 |  | 3.049 |  | -0.152 |  | -0.866 |  | 0.392 |  |
|  |  | ICA_GM_13 |  | -0.844 |  | 4.839 |  | -0.046 |  | -0.174 |  | 0.862 |  |
|  |  | ICA_GM_14 |  | 2.305 |  | 2.472 |  | 0.121 |  | 0.932 |  | 0.357 |  |
|  |  | ICA_GM_15 |  | 3.400 |  | 7.815 |  | 0.192 |  | 0.435 |  | 0.666 |  |
|  |  | ICA_GM_16 |  | -2.744 |  | 3.667 |  | -0.153 |  | -0.748 |  | 0.459 |  |
|  |  | ICA_GM_17 |  | -2.129 |  | 4.285 |  | -0.107 |  | -0.497 |  | 0.622 |  |
|  |  | ICA_GM_18 |  | 0.851 |  | 3.047 |  | 0.048 |  | 0.279 |  | 0.782 |  |
|  |  | ICA_GM_19 |  | -4.365 |  | 4.046 |  | -0.238 |  | -1.079 |  | 0.288 |  |
|  |  | ICA_GM_20 |  | -1.827 |  | 3.115 |  | -0.097 |  | -0.586 |  | 0.561 |  |
|  |  | ICA_GM_21 |  | 2.631 |  | 3.883 |  | 0.140 |  | 0.677 |  | 0.502 |  |
|  |  | ICA_GM_22 |  | -1.249 |  | 3.189 |  | -0.076 |  | -0.392 |  | 0.698 |  |
|  |  | ICA_GM_23 |  | -9.433 |  | 6.557 |  | -0.557 |  | -1.439 |  | 0.159 |  |
|  |  | ICA_GM_24 |  | 0.760 |  | 3.283 |  | 0.039 |  | 0.232 |  | 0.818 |  |
|  |  | ICA_GM_25 |  | -3.062 |  | 4.154 |  | -0.157 |  | -0.737 |  | 0.466 |  |
|  |  | ICA_GM_26 |  | -0.794 |  | 2.481 |  | -0.042 |  | -0.320 |  | 0.751 |  |
|  |  | ICA_GM_27 |  | 0.931 |  | 2.171 |  | 0.058 |  | 0.429 |  | 0.671 |  |
|  |  | ICA_GM_28 |  | -2.123 |  | 2.496 |  | -0.113 |  | -0.851 |  | 0.400 |  |
| 4 |  | (Intercept) |  | 24.437 |  | 37.697 |  |  |  | 0.648 |  | 0.521 |  |
|  |  | ICA_GM_01 |  | 1.963 |  | 2.229 |  | 0.137 |  | 0.881 |  | 0.384 |  |
|  |  | ICA_GM_02 |  | -2.778 |  | 5.041 |  | -0.205 |  | -0.551 |  | 0.585 |  |
|  |  | ICA_GM_03 |  | 22.762 |  | 36.221 |  | 0.494 |  | 0.628 |  | 0.533 |  |
|  |  | ICA_GM_04 |  | -2.456 |  | 2.573 |  | -0.159 |  | -0.954 |  | 0.346 |  |
|  |  | ICA_GM_05 |  | 4.268 |  | 3.917 |  | 0.248 |  | 1.089 |  | 0.283 |  |
|  |  | ICA_GM_07 |  | -0.471 |  | 2.453 |  | -0.026 |  | -0.192 |  | 0.849 |  |
|  |  | ICA_GM_08 |  | 1.496 |  | 2.353 |  | 0.083 |  | 0.636 |  | 0.529 |  |
|  |  | ICA_GM_09 |  | 1.029 |  | 2.504 |  | 0.054 |  | 0.411 |  | 0.683 |  |
|  |  | ICA_GM_10 |  | -2.510 |  | 2.793 |  | -0.144 |  | -0.899 |  | 0.375 |  |
|  |  | ICA_GM_13 |  | -0.560 |  | 4.103 |  | -0.031 |  | -0.137 |  | 0.892 |  |
|  |  | ICA_GM_14 |  | 2.285 |  | 2.434 |  | 0.120 |  | 0.939 |  | 0.354 |  |
|  |  | ICA_GM_15 |  | 2.907 |  | 6.443 |  | 0.164 |  | 0.451 |  | 0.654 |  |
|  |  | ICA_GM_16 |  | -2.557 |  | 3.241 |  | -0.143 |  | -0.789 |  | 0.435 |  |
|  |  | ICA_GM_17 |  | -1.911 |  | 3.793 |  | -0.096 |  | -0.504 |  | 0.617 |  |
|  |  | ICA_GM_18 |  | 0.996 |  | 2.739 |  | 0.056 |  | 0.363 |  | 0.718 |  |
|  |  | ICA_GM_19 |  | -4.145 |  | 3.515 |  | -0.226 |  | -1.179 |  | 0.246 |  |
|  |  | ICA_GM_20 |  | -1.950 |  | 2.885 |  | -0.103 |  | -0.676 |  | 0.503 |  |
|  |  | ICA_GM_21 |  | 2.439 |  | 3.460 |  | 0.130 |  | 0.705 |  | 0.485 |  |
|  |  | ICA_GM_22 |  | -1.073 |  | 2.761 |  | -0.066 |  | -0.389 |  | 0.700 |  |
|  |  | ICA_GM_23 |  | -9.036 |  | 5.495 |  | -0.534 |  | -1.644 |  | 0.108 |  |
|  |  | ICA_GM_24 |  | 0.896 |  | 3.024 |  | 0.046 |  | 0.296 |  | 0.769 |  |
|  |  | ICA_GM_25 |  | -2.860 |  | 3.711 |  | -0.146 |  | -0.770 |  | 0.446 |  |
|  |  | ICA_GM_26 |  | -0.804 |  | 2.446 |  | -0.043 |  | -0.329 |  | 0.744 |  |
|  |  | ICA_GM_27 |  | 0.946 |  | 2.139 |  | 0.059 |  | 0.442 |  | 0.661 |  |
|  |  | ICA_GM_28 |  | -2.092 |  | 2.449 |  | -0.112 |  | -0.854 |  | 0.398 |  |
| 5 |  | (Intercept) |  | 20.197 |  | 21.098 |  |  |  | 0.957 |  | 0.344 |  |
|  |  | ICA_GM_01 |  | 1.834 |  | 1.992 |  | 0.128 |  | 0.921 |  | 0.363 |  |
|  |  | ICA_GM_02 |  | -2.240 |  | 3.104 |  | -0.165 |  | -0.722 |  | 0.475 |  |
|  |  | ICA_GM_03 |  | 18.705 |  | 20.441 |  | 0.406 |  | 0.915 |  | 0.366 |  |
|  |  | ICA_GM_04 |  | -2.289 |  | 2.237 |  | -0.148 |  | -1.023 |  | 0.312 |  |
|  |  | ICA_GM_05 |  | 4.631 |  | 2.839 |  | 0.269 |  | 1.631 |  | 0.111 |  |
|  |  | ICA_GM_07 |  | -0.376 |  | 2.322 |  | -0.020 |  | -0.162 |  | 0.872 |  |
|  |  | ICA_GM_08 |  | 1.430 |  | 2.274 |  | 0.080 |  | 0.629 |  | 0.533 |  |
|  |  | ICA_GM_09 |  | 0.975 |  | 2.441 |  | 0.051 |  | 0.399 |  | 0.692 |  |
|  |  | ICA_GM_10 |  | -2.321 |  | 2.396 |  | -0.133 |  | -0.969 |  | 0.339 |  |
|  |  | ICA_GM_14 |  | 2.290 |  | 2.403 |  | 0.121 |  | 0.953 |  | 0.346 |  |
|  |  | ICA_GM_15 |  | 2.230 |  | 4.063 |  | 0.126 |  | 0.549 |  | 0.586 |  |
|  |  | ICA_GM_16 |  | -2.303 |  | 2.623 |  | -0.128 |  | -0.878 |  | 0.385 |  |
|  |  | ICA_GM_17 |  | -1.604 |  | 3.013 |  | -0.081 |  | -0.532 |  | 0.597 |  |
|  |  | ICA_GM_18 |  | 1.143 |  | 2.488 |  | 0.065 |  | 0.459 |  | 0.649 |  |
|  |  | ICA_GM_19 |  | -3.859 |  | 2.790 |  | -0.210 |  | -1.384 |  | 0.174 |  |
|  |  | ICA_GM_20 |  | -2.128 |  | 2.542 |  | -0.113 |  | -0.837 |  | 0.408 |  |
|  |  | ICA_GM_21 |  | 2.152 |  | 2.714 |  | 0.115 |  | 0.793 |  | 0.433 |  |
|  |  | ICA_GM_22 |  | -0.865 |  | 2.274 |  | -0.053 |  | -0.380 |  | 0.706 |  |
|  |  | ICA_GM_23 |  | -8.475 |  | 3.599 |  | -0.500 |  | -2.354 |  | 0.024 |  |
|  |  | ICA_GM_24 |  | 1.084 |  | 2.657 |  | 0.056 |  | 0.408 |  | 0.686 |  |
|  |  | ICA_GM_25 |  | -2.566 |  | 2.985 |  | -0.131 |  | -0.860 |  | 0.395 |  |
|  |  | ICA_GM_26 |  | -0.804 |  | 2.416 |  | -0.043 |  | -0.333 |  | 0.741 |  |
|  |  | ICA_GM_27 |  | 0.978 |  | 2.099 |  | 0.061 |  | 0.466 |  | 0.644 |  |
|  |  | ICA_GM_28 |  | -2.032 |  | 2.378 |  | -0.109 |  | -0.854 |  | 0.398 |  |
| 6 |  | (Intercept) |  | 19.725 |  | 20.639 |  |  |  | 0.956 |  | 0.345 |  |
|  |  | ICA_GM_01 |  | 1.807 |  | 1.961 |  | 0.126 |  | 0.921 |  | 0.362 |  |
|  |  | ICA_GM_02 |  | -2.181 |  | 3.045 |  | -0.161 |  | -0.716 |  | 0.478 |  |
|  |  | ICA_GM_03 |  | 18.251 |  | 20.000 |  | 0.396 |  | 0.913 |  | 0.367 |  |
|  |  | ICA_GM_04 |  | -2.249 |  | 2.196 |  | -0.146 |  | -1.024 |  | 0.312 |  |
|  |  | ICA_GM_05 |  | 4.676 |  | 2.791 |  | 0.272 |  | 1.675 |  | 0.102 |  |
|  |  | ICA_GM_08 |  | 1.408 |  | 2.242 |  | 0.079 |  | 0.628 |  | 0.534 |  |
|  |  | ICA_GM_09 |  | 0.951 |  | 2.407 |  | 0.050 |  | 0.395 |  | 0.695 |  |
|  |  | ICA_GM_10 |  | -2.287 |  | 2.357 |  | -0.131 |  | -0.970 |  | 0.338 |  |
|  |  | ICA_GM_14 |  | 2.298 |  | 2.373 |  | 0.121 |  | 0.968 |  | 0.339 |  |
|  |  | ICA_GM_15 |  | 2.170 |  | 3.996 |  | 0.123 |  | 0.543 |  | 0.590 |  |
|  |  | ICA_GM_16 |  | -2.269 |  | 2.583 |  | -0.127 |  | -0.879 |  | 0.385 |  |
|  |  | ICA_GM_17 |  | -1.568 |  | 2.968 |  | -0.079 |  | -0.528 |  | 0.600 |  |
|  |  | ICA_GM_18 |  | 1.179 |  | 2.447 |  | 0.067 |  | 0.482 |  | 0.633 |  |
|  |  | ICA_GM_19 |  | -3.833 |  | 2.751 |  | -0.209 |  | -1.393 |  | 0.171 |  |
|  |  | ICA_GM_20 |  | -2.160 |  | 2.503 |  | -0.114 |  | -0.863 |  | 0.393 |  |
|  |  | ICA_GM_21 |  | 2.118 |  | 2.672 |  | 0.113 |  | 0.793 |  | 0.433 |  |
|  |  | ICA_GM_22 |  | -0.834 |  | 2.238 |  | -0.051 |  | -0.373 |  | 0.711 |  |
|  |  | ICA_GM_23 |  | -8.398 |  | 3.525 |  | -0.496 |  | -2.383 |  | 0.022 |  |
|  |  | ICA_GM_24 |  | 1.109 |  | 2.620 |  | 0.057 |  | 0.423 |  | 0.674 |  |
|  |  | ICA_GM_25 |  | -2.512 |  | 2.930 |  | -0.128 |  | -0.857 |  | 0.396 |  |
|  |  | ICA_GM_26 |  | -0.819 |  | 2.384 |  | -0.044 |  | -0.344 |  | 0.733 |  |
|  |  | ICA_GM_27 |  | 0.987 |  | 2.072 |  | 0.062 |  | 0.476 |  | 0.637 |  |
|  |  | ICA_GM_28 |  | -2.041 |  | 2.349 |  | -0.109 |  | -0.869 |  | 0.390 |  |
| 7 |  | (Intercept) |  | 19.642 |  | 20.415 |  |  |  | 0.962 |  | 0.342 |  |
|  |  | ICA_GM_01 |  | 1.734 |  | 1.928 |  | 0.121 |  | 0.899 |  | 0.374 |  |
|  |  | ICA_GM_02 |  | -2.214 |  | 3.010 |  | -0.163 |  | -0.735 |  | 0.466 |  |
|  |  | ICA_GM_03 |  | 18.186 |  | 19.783 |  | 0.394 |  | 0.919 |  | 0.363 |  |
|  |  | ICA_GM_04 |  | -2.200 |  | 2.167 |  | -0.142 |  | -1.015 |  | 0.316 |  |
|  |  | ICA_GM_05 |  | 4.641 |  | 2.759 |  | 0.270 |  | 1.682 |  | 0.100 |  |
|  |  | ICA_GM_08 |  | 1.375 |  | 2.216 |  | 0.077 |  | 0.621 |  | 0.538 |  |
|  |  | ICA_GM_09 |  | 0.938 |  | 2.381 |  | 0.049 |  | 0.394 |  | 0.696 |  |
|  |  | ICA_GM_10 |  | -2.279 |  | 2.332 |  | -0.131 |  | -0.977 |  | 0.334 |  |
|  |  | ICA_GM_14 |  | 2.261 |  | 2.345 |  | 0.119 |  | 0.964 |  | 0.341 |  |
|  |  | ICA_GM_15 |  | 2.097 |  | 3.947 |  | 0.119 |  | 0.531 |  | 0.598 |  |
|  |  | ICA_GM_16 |  | -2.263 |  | 2.555 |  | -0.126 |  | -0.886 |  | 0.381 |  |
|  |  | ICA_GM_17 |  | -1.590 |  | 2.935 |  | -0.080 |  | -0.542 |  | 0.591 |  |
|  |  | ICA_GM_18 |  | 1.106 |  | 2.412 |  | 0.063 |  | 0.458 |  | 0.649 |  |
|  |  | ICA_GM_19 |  | -3.842 |  | 2.721 |  | -0.209 |  | -1.412 |  | 0.165 |  |
|  |  | ICA_GM_20 |  | -2.206 |  | 2.472 |  | -0.117 |  | -0.892 |  | 0.377 |  |
|  |  | ICA_GM_21 |  | 2.101 |  | 2.643 |  | 0.112 |  | 0.795 |  | 0.431 |  |
|  |  | ICA_GM_22 |  | -0.807 |  | 2.213 |  | -0.049 |  | -0.365 |  | 0.717 |  |
|  |  | ICA_GM_23 |  | -8.346 |  | 3.483 |  | -0.493 |  | -2.396 |  | 0.021 |  |
|  |  | ICA_GM_24 |  | 1.101 |  | 2.592 |  | 0.057 |  | 0.425 |  | 0.673 |  |
|  |  | ICA_GM_25 |  | -2.535 |  | 2.898 |  | -0.130 |  | -0.875 |  | 0.387 |  |
|  |  | ICA_GM_27 |  | 0.984 |  | 2.050 |  | 0.061 |  | 0.480 |  | 0.634 |  |
|  |  | ICA_GM_28 |  | -2.060 |  | 2.323 |  | -0.110 |  | -0.887 |  | 0.380 |  |
| 8 |  | (Intercept) |  | 16.754 |  | 18.620 |  |  |  | 0.900 |  | 0.373 |  |
|  |  | ICA_GM_01 |  | 1.610 |  | 1.878 |  | 0.112 |  | 0.857 |  | 0.396 |  |
|  |  | ICA_GM_02 |  | -1.833 |  | 2.794 |  | -0.135 |  | -0.656 |  | 0.515 |  |
|  |  | ICA_GM_03 |  | 15.421 |  | 18.082 |  | 0.334 |  | 0.853 |  | 0.399 |  |
|  |  | ICA_GM_04 |  | -2.039 |  | 2.100 |  | -0.132 |  | -0.971 |  | 0.337 |  |
|  |  | ICA_GM_05 |  | 4.908 |  | 2.633 |  | 0.286 |  | 1.864 |  | 0.069 |  |
|  |  | ICA_GM_08 |  | 1.323 |  | 2.188 |  | 0.074 |  | 0.604 |  | 0.549 |  |
|  |  | ICA_GM_09 |  | 0.863 |  | 2.347 |  | 0.045 |  | 0.368 |  | 0.715 |  |
|  |  | ICA_GM_10 |  | -2.213 |  | 2.301 |  | -0.127 |  | -0.962 |  | 0.342 |  |
|  |  | ICA_GM_14 |  | 2.212 |  | 2.317 |  | 0.117 |  | 0.955 |  | 0.345 |  |
|  |  | ICA_GM_15 |  | 1.684 |  | 3.743 |  | 0.095 |  | 0.450 |  | 0.655 |  |
|  |  | ICA_GM_16 |  | -2.116 |  | 2.497 |  | -0.118 |  | -0.848 |  | 0.401 |  |
|  |  | ICA_GM_17 |  | -1.329 |  | 2.816 |  | -0.067 |  | -0.472 |  | 0.640 |  |
|  |  | ICA_GM_18 |  | 1.190 |  | 2.375 |  | 0.067 |  | 0.501 |  | 0.619 |  |
|  |  | ICA_GM_19 |  | -3.700 |  | 2.665 |  | -0.201 |  | -1.388 |  | 0.172 |  |
|  |  | ICA_GM_20 |  | -2.254 |  | 2.443 |  | -0.119 |  | -0.922 |  | 0.362 |  |
|  |  | ICA_GM_21 |  | 1.836 |  | 2.515 |  | 0.098 |  | 0.730 |  | 0.469 |  |
|  |  | ICA_GM_23 |  | -7.971 |  | 3.293 |  | -0.471 |  | -2.420 |  | 0.020 |  |
|  |  | ICA_GM_24 |  | 1.262 |  | 2.527 |  | 0.065 |  | 0.499 |  | 0.620 |  |
|  |  | ICA_GM_25 |  | -2.245 |  | 2.757 |  | -0.115 |  | -0.814 |  | 0.420 |  |
|  |  | ICA_GM_27 |  | 0.990 |  | 2.028 |  | 0.062 |  | 0.488 |  | 0.628 |  |
|  |  | ICA_GM_28 |  | -1.970 |  | 2.286 |  | -0.105 |  | -0.862 |  | 0.394 |  |
| 9 |  | (Intercept) |  | 16.246 |  | 18.381 |  |  |  | 0.884 |  | 0.382 |  |
|  |  | ICA_GM_01 |  | 1.595 |  | 1.859 |  | 0.111 |  | 0.858 |  | 0.396 |  |
|  |  | ICA_GM_02 |  | -1.871 |  | 2.764 |  | -0.138 |  | -0.677 |  | 0.502 |  |
|  |  | ICA_GM_03 |  | 14.894 |  | 17.843 |  | 0.323 |  | 0.835 |  | 0.408 |  |
|  |  | ICA_GM_04 |  | -2.035 |  | 2.079 |  | -0.132 |  | -0.979 |  | 0.333 |  |
|  |  | ICA_GM_05 |  | 4.900 |  | 2.606 |  | 0.285 |  | 1.880 |  | 0.067 |  |
|  |  | ICA_GM_08 |  | 1.341 |  | 2.166 |  | 0.075 |  | 0.619 |  | 0.539 |  |
|  |  | ICA_GM_10 |  | -2.197 |  | 2.277 |  | -0.126 |  | -0.965 |  | 0.340 |  |
|  |  | ICA_GM_14 |  | 2.215 |  | 2.294 |  | 0.117 |  | 0.966 |  | 0.340 |  |
|  |  | ICA_GM_15 |  | 1.636 |  | 3.702 |  | 0.093 |  | 0.442 |  | 0.661 |  |
|  |  | ICA_GM_16 |  | -2.021 |  | 2.458 |  | -0.113 |  | -0.822 |  | 0.416 |  |
|  |  | ICA_GM_17 |  | -1.344 |  | 2.788 |  | -0.068 |  | -0.482 |  | 0.632 |  |
|  |  | ICA_GM_18 |  | 1.231 |  | 2.349 |  | 0.070 |  | 0.524 |  | 0.603 |  |
|  |  | ICA_GM_19 |  | -3.623 |  | 2.630 |  | -0.197 |  | -1.378 |  | 0.175 |  |
|  |  | ICA_GM_20 |  | -2.302 |  | 2.415 |  | -0.122 |  | -0.953 |  | 0.346 |  |
|  |  | ICA_GM_21 |  | 1.825 |  | 2.490 |  | 0.097 |  | 0.733 |  | 0.468 |  |
|  |  | ICA_GM_23 |  | -7.899 |  | 3.254 |  | -0.466 |  | -2.427 |  | 0.019 |  |
|  |  | ICA_GM_24 |  | 1.317 |  | 2.497 |  | 0.068 |  | 0.527 |  | 0.601 |  |
|  |  | ICA_GM_25 |  | -2.208 |  | 2.727 |  | -0.113 |  | -0.809 |  | 0.423 |  |
|  |  | ICA_GM_27 |  | 0.967 |  | 2.007 |  | 0.060 |  | 0.482 |  | 0.632 |  |
|  |  | ICA_GM_28 |  | -1.913 |  | 2.257 |  | -0.102 |  | -0.847 |  | 0.401 |  |
| 10 |  | (Intercept) |  | 9.680 |  | 10.716 |  |  |  | 0.903 |  | 0.371 |  |
|  |  | ICA_GM_01 |  | 1.521 |  | 1.834 |  | 0.106 |  | 0.829 |  | 0.411 |  |
|  |  | ICA_GM_02 |  | -1.074 |  | 2.075 |  | -0.079 |  | -0.518 |  | 0.607 |  |
|  |  | ICA_GM_03 |  | 8.593 |  | 10.627 |  | 0.186 |  | 0.809 |  | 0.423 |  |
|  |  | ICA_GM_04 |  | -1.741 |  | 1.952 |  | -0.113 |  | -0.892 |  | 0.377 |  |
|  |  | ICA_GM_05 |  | 5.465 |  | 2.250 |  | 0.318 |  | 2.428 |  | 0.019 |  |
|  |  | ICA_GM_08 |  | 1.294 |  | 2.143 |  | 0.072 |  | 0.604 |  | 0.549 |  |
|  |  | ICA_GM_10 |  | -1.938 |  | 2.179 |  | -0.111 |  | -0.889 |  | 0.379 |  |
|  |  | ICA_GM_14 |  | 2.184 |  | 2.271 |  | 0.115 |  | 0.962 |  | 0.342 |  |
|  |  | ICA_GM_16 |  | -1.628 |  | 2.271 |  | -0.091 |  | -0.717 |  | 0.477 |  |
|  |  | ICA_GM_17 |  | -0.857 |  | 2.537 |  | -0.043 |  | -0.338 |  | 0.737 |  |
|  |  | ICA_GM_18 |  | 1.629 |  | 2.150 |  | 0.092 |  | 0.758 |  | 0.453 |  |
|  |  | ICA_GM_19 |  | -3.140 |  | 2.370 |  | -0.171 |  | -1.325 |  | 0.192 |  |
|  |  | ICA_GM_20 |  | -2.605 |  | 2.295 |  | -0.138 |  | -1.135 |  | 0.262 |  |
|  |  | ICA_GM_21 |  | 1.445 |  | 2.315 |  | 0.077 |  | 0.624 |  | 0.536 |  |
|  |  | ICA_GM_23 |  | -7.032 |  | 2.574 |  | -0.415 |  | -2.733 |  | 0.009 |  |
|  |  | ICA_GM_24 |  | 1.676 |  | 2.340 |  | 0.086 |  | 0.716 |  | 0.478 |  |
|  |  | ICA_GM_25 |  | -1.726 |  | 2.477 |  | -0.088 |  | -0.697 |  | 0.490 |  |
|  |  | ICA_GM_27 |  | 0.952 |  | 1.988 |  | 0.059 |  | 0.479 |  | 0.635 |  |
|  |  | ICA_GM_28 |  | -1.910 |  | 2.236 |  | -0.102 |  | -0.854 |  | 0.398 |  |
| 11 |  | (Intercept) |  | 8.262 |  | 9.762 |  |  |  | 0.846 |  | 0.402 |  |
|  |  | ICA_GM_01 |  | 1.451 |  | 1.805 |  | 0.101 |  | 0.804 |  | 0.426 |  |
|  |  | ICA_GM_02 |  | -0.915 |  | 2.001 |  | -0.067 |  | -0.457 |  | 0.650 |  |
|  |  | ICA_GM_03 |  | 7.230 |  | 9.732 |  | 0.157 |  | 0.743 |  | 0.461 |  |
|  |  | ICA_GM_04 |  | -1.640 |  | 1.909 |  | -0.106 |  | -0.859 |  | 0.395 |  |
|  |  | ICA_GM_05 |  | 5.589 |  | 2.198 |  | 0.325 |  | 2.543 |  | 0.014 |  |
|  |  | ICA_GM_08 |  | 1.266 |  | 2.120 |  | 0.071 |  | 0.597 |  | 0.553 |  |
|  |  | ICA_GM_10 |  | -1.825 |  | 2.132 |  | -0.105 |  | -0.856 |  | 0.397 |  |
|  |  | ICA_GM_14 |  | 2.185 |  | 2.249 |  | 0.115 |  | 0.972 |  | 0.336 |  |
|  |  | ICA_GM_16 |  | -1.551 |  | 2.237 |  | -0.086 |  | -0.693 |  | 0.492 |  |
|  |  | ICA_GM_18 |  | 1.639 |  | 2.128 |  | 0.093 |  | 0.770 |  | 0.445 |  |
|  |  | ICA_GM_19 |  | -3.053 |  | 2.333 |  | -0.166 |  | -1.309 |  | 0.197 |  |
|  |  | ICA_GM_20 |  | -2.672 |  | 2.263 |  | -0.141 |  | -1.181 |  | 0.244 |  |
|  |  | ICA_GM_21 |  | 1.417 |  | 2.291 |  | 0.076 |  | 0.619 |  | 0.539 |  |
|  |  | ICA_GM_23 |  | -6.844 |  | 2.488 |  | -0.404 |  | -2.751 |  | 0.009 |  |
|  |  | ICA_GM_24 |  | 1.715 |  | 2.314 |  | 0.088 |  | 0.741 |  | 0.462 |  |
|  |  | ICA_GM_25 |  | -1.657 |  | 2.444 |  | -0.085 |  | -0.678 |  | 0.501 |  |
|  |  | ICA_GM_27 |  | 0.988 |  | 1.966 |  | 0.062 |  | 0.503 |  | 0.618 |  |
|  |  | ICA_GM_28 |  | -1.893 |  | 2.214 |  | -0.101 |  | -0.855 |  | 0.397 |  |
| 12 |  | (Intercept) |  | 5.641 |  | 7.836 |  |  |  | 0.720 |  | 0.475 |  |
|  |  | ICA_GM_01 |  | 1.235 |  | 1.726 |  | 0.086 |  | 0.715 |  | 0.478 |  |
|  |  | ICA_GM_03 |  | 4.689 |  | 7.923 |  | 0.102 |  | 0.592 |  | 0.557 |  |
|  |  | ICA_GM_04 |  | -1.536 |  | 1.879 |  | -0.099 |  | -0.817 |  | 0.418 |  |
|  |  | ICA_GM_05 |  | 5.696 |  | 2.167 |  | 0.331 |  | 2.629 |  | 0.012 |  |
|  |  | ICA_GM_08 |  | 1.253 |  | 2.102 |  | 0.070 |  | 0.596 |  | 0.554 |  |
|  |  | ICA_GM_10 |  | -1.647 |  | 2.078 |  | -0.095 |  | -0.792 |  | 0.432 |  |
|  |  | ICA_GM_14 |  | 2.087 |  | 2.219 |  | 0.110 |  | 0.940 |  | 0.352 |  |
|  |  | ICA_GM_16 |  | -1.371 |  | 2.183 |  | -0.076 |  | -0.628 |  | 0.533 |  |
|  |  | ICA_GM_18 |  | 1.704 |  | 2.105 |  | 0.096 |  | 0.809 |  | 0.422 |  |
|  |  | ICA_GM_19 |  | -2.925 |  | 2.296 |  | -0.159 |  | -1.274 |  | 0.209 |  |
|  |  | ICA_GM_20 |  | -2.720 |  | 2.241 |  | -0.144 |  | -1.214 |  | 0.231 |  |
|  |  | ICA_GM_21 |  | 1.274 |  | 2.250 |  | 0.068 |  | 0.566 |  | 0.574 |  |
|  |  | ICA_GM_23 |  | -6.451 |  | 2.314 |  | -0.381 |  | -2.788 |  | 0.008 |  |
|  |  | ICA_GM_24 |  | 1.759 |  | 2.292 |  | 0.091 |  | 0.767 |  | 0.447 |  |
|  |  | ICA_GM_25 |  | -1.519 |  | 2.405 |  | -0.078 |  | -0.632 |  | 0.531 |  |
|  |  | ICA_GM_27 |  | 0.909 |  | 1.941 |  | 0.057 |  | 0.468 |  | 0.642 |  |
|  |  | ICA_GM_28 |  | -1.830 |  | 2.190 |  | -0.098 |  | -0.835 |  | 0.408 |  |
| 13 |  | (Intercept) |  | 5.970 |  | 7.739 |  |  |  | 0.771 |  | 0.444 |  |
|  |  | ICA_GM_01 |  | 1.249 |  | 1.712 |  | 0.087 |  | 0.730 |  | 0.469 |  |
|  |  | ICA_GM_03 |  | 5.002 |  | 7.829 |  | 0.108 |  | 0.639 |  | 0.526 |  |
|  |  | ICA_GM_04 |  | -1.611 |  | 1.857 |  | -0.104 |  | -0.868 |  | 0.390 |  |
|  |  | ICA_GM_05 |  | 5.737 |  | 2.147 |  | 0.334 |  | 2.673 |  | 0.010 |  |
|  |  | ICA_GM_08 |  | 1.324 |  | 2.079 |  | 0.074 |  | 0.637 |  | 0.527 |  |
|  |  | ICA_GM_10 |  | -1.783 |  | 2.041 |  | -0.102 |  | -0.874 |  | 0.387 |  |
|  |  | ICA_GM_14 |  | 2.190 |  | 2.190 |  | 0.115 |  | 1.000 |  | 0.322 |  |
|  |  | ICA_GM_16 |  | -1.383 |  | 2.165 |  | -0.077 |  | -0.639 |  | 0.526 |  |
|  |  | ICA_GM_18 |  | 1.604 |  | 2.077 |  | 0.091 |  | 0.772 |  | 0.444 |  |
|  |  | ICA_GM_19 |  | -3.071 |  | 2.256 |  | -0.167 |  | -1.361 |  | 0.180 |  |
|  |  | ICA_GM_20 |  | -2.780 |  | 2.219 |  | -0.147 |  | -1.253 |  | 0.217 |  |
|  |  | ICA_GM_21 |  | 1.265 |  | 2.231 |  | 0.068 |  | 0.567 |  | 0.573 |  |
|  |  | ICA_GM_23 |  | -6.475 |  | 2.294 |  | -0.382 |  | -2.823 |  | 0.007 |  |
|  |  | ICA_GM_24 |  | 1.668 |  | 2.264 |  | 0.086 |  | 0.737 |  | 0.465 |  |
|  |  | ICA_GM_25 |  | -1.431 |  | 2.378 |  | -0.073 |  | -0.602 |  | 0.550 |  |
|  |  | ICA_GM_28 |  | -1.875 |  | 2.170 |  | -0.100 |  | -0.864 |  | 0.392 |  |
| 14 |  | (Intercept) |  | 4.743 |  | 7.378 |  |  |  | 0.643 |  | 0.523 |  |
|  |  | ICA_GM_01 |  | 1.262 |  | 1.699 |  | 0.088 |  | 0.743 |  | 0.461 |  |
|  |  | ICA_GM_03 |  | 3.839 |  | 7.502 |  | 0.083 |  | 0.512 |  | 0.611 |  |
|  |  | ICA_GM_04 |  | -1.566 |  | 1.842 |  | -0.101 |  | -0.850 |  | 0.399 |  |
|  |  | ICA_GM_05 |  | 5.820 |  | 2.127 |  | 0.339 |  | 2.737 |  | 0.009 |  |
|  |  | ICA_GM_08 |  | 1.248 |  | 2.060 |  | 0.070 |  | 0.606 |  | 0.547 |  |
|  |  | ICA_GM_10 |  | -1.703 |  | 2.021 |  | -0.098 |  | -0.843 |  | 0.404 |  |
|  |  | ICA_GM_14 |  | 2.238 |  | 2.173 |  | 0.118 |  | 1.030 |  | 0.308 |  |
|  |  | ICA_GM_16 |  | -1.317 |  | 2.146 |  | -0.073 |  | -0.613 |  | 0.542 |  |
|  |  | ICA_GM_18 |  | 1.691 |  | 2.056 |  | 0.096 |  | 0.822 |  | 0.415 |  |
|  |  | ICA_GM_19 |  | -2.979 |  | 2.234 |  | -0.162 |  | -1.334 |  | 0.189 |  |
|  |  | ICA_GM_20 |  | -2.766 |  | 2.203 |  | -0.146 |  | -1.255 |  | 0.215 |  |
|  |  | ICA_GM_23 |  | -6.341 |  | 2.266 |  | -0.374 |  | -2.799 |  | 0.007 |  |
|  |  | ICA_GM_24 |  | 1.713 |  | 2.247 |  | 0.088 |  | 0.762 |  | 0.450 |  |
|  |  | ICA_GM_25 |  | -1.349 |  | 2.356 |  | -0.069 |  | -0.573 |  | 0.570 |  |
|  |  | ICA_GM_28 |  | -1.840 |  | 2.154 |  | -0.098 |  | -0.854 |  | 0.397 |  |
| 15 |  | (Intercept) |  | 0.974 |  | 0.441 |  |  |  | 2.209 |  | 0.032 |  |
|  |  | ICA_GM_01 |  | 1.118 |  | 1.663 |  | 0.078 |  | 0.672 |  | 0.505 |  |
|  |  | ICA_GM_04 |  | -1.285 |  | 1.745 |  | -0.083 |  | -0.736 |  | 0.465 |  |
|  |  | ICA_GM_05 |  | 6.198 |  | 1.979 |  | 0.361 |  | 3.133 |  | 0.003 |  |
|  |  | ICA_GM_08 |  | 1.183 |  | 2.041 |  | 0.066 |  | 0.580 |  | 0.565 |  |
|  |  | ICA_GM_10 |  | -1.524 |  | 1.976 |  | -0.087 |  | -0.771 |  | 0.444 |  |
|  |  | ICA_GM_14 |  | 2.207 |  | 2.155 |  | 0.116 |  | 1.024 |  | 0.311 |  |
|  |  | ICA_GM_16 |  | -1.019 |  | 2.051 |  | -0.057 |  | -0.497 |  | 0.621 |  |
|  |  | ICA_GM_18 |  | 1.750 |  | 2.038 |  | 0.099 |  | 0.859 |  | 0.395 |  |
|  |  | ICA_GM_19 |  | -2.659 |  | 2.128 |  | -0.145 |  | -1.249 |  | 0.217 |  |
|  |  | ICA_GM_20 |  | -2.910 |  | 2.169 |  | -0.154 |  | -1.342 |  | 0.186 |  |
|  |  | ICA_GM_23 |  | -5.759 |  | 1.944 |  | -0.340 |  | -2.962 |  | 0.005 |  |
|  |  | ICA_GM_24 |  | 1.866 |  | 2.210 |  | 0.096 |  | 0.844 |  | 0.403 |  |
|  |  | ICA_GM_25 |  | -0.971 |  | 2.220 |  | -0.050 |  | -0.437 |  | 0.664 |  |
|  |  | ICA_GM_28 |  | -1.825 |  | 2.137 |  | -0.098 |  | -0.854 |  | 0.397 |  |
| 16 |  | (Intercept) |  | 0.916 |  | 0.417 |  |  |  | 2.196 |  | 0.033 |  |
|  |  | ICA_GM_01 |  | 1.161 |  | 1.647 |  | 0.081 |  | 0.705 |  | 0.484 |  |
|  |  | ICA_GM_04 |  | -1.313 |  | 1.729 |  | -0.085 |  | -0.759 |  | 0.451 |  |
|  |  | ICA_GM_05 |  | 6.144 |  | 1.959 |  | 0.358 |  | 3.137 |  | 0.003 |  |
|  |  | ICA_GM_08 |  | 1.144 |  | 2.022 |  | 0.064 |  | 0.566 |  | 0.574 |  |
|  |  | ICA_GM_10 |  | -1.501 |  | 1.959 |  | -0.086 |  | -0.766 |  | 0.447 |  |
|  |  | ICA_GM_14 |  | 2.184 |  | 2.137 |  | 0.115 |  | 1.022 |  | 0.312 |  |
|  |  | ICA_GM_16 |  | -1.041 |  | 2.033 |  | -0.058 |  | -0.512 |  | 0.611 |  |
|  |  | ICA_GM_18 |  | 1.769 |  | 2.021 |  | 0.100 |  | 0.876 |  | 0.385 |  |
|  |  | ICA_GM_19 |  | -2.622 |  | 2.109 |  | -0.143 |  | -1.243 |  | 0.220 |  |
|  |  | ICA_GM_20 |  | -2.853 |  | 2.147 |  | -0.151 |  | -1.329 |  | 0.190 |  |
|  |  | ICA_GM_23 |  | -5.743 |  | 1.928 |  | -0.339 |  | -2.978 |  | 0.004 |  |
|  |  | ICA_GM_24 |  | 1.842 |  | 2.191 |  | 0.095 |  | 0.840 |  | 0.405 |  |
|  |  | ICA_GM_28 |  | -1.774 |  | 2.117 |  | -0.095 |  | -0.838 |  | 0.406 |  |
| 17 |  | (Intercept) |  | 0.871 |  | 0.405 |  |  |  | 2.151 |  | 0.036 |  |
|  |  | ICA_GM_01 |  | 1.108 |  | 1.631 |  | 0.077 |  | 0.679 |  | 0.500 |  |
|  |  | ICA_GM_04 |  | -1.289 |  | 1.716 |  | -0.083 |  | -0.751 |  | 0.456 |  |
|  |  | ICA_GM_05 |  | 6.245 |  | 1.935 |  | 0.363 |  | 3.228 |  | 0.002 |  |
|  |  | ICA_GM_08 |  | 1.128 |  | 2.007 |  | 0.063 |  | 0.562 |  | 0.577 |  |
|  |  | ICA_GM_10 |  | -1.428 |  | 1.940 |  | -0.082 |  | -0.736 |  | 0.465 |  |
|  |  | ICA_GM_14 |  | 2.259 |  | 2.117 |  | 0.119 |  | 1.067 |  | 0.291 |  |
|  |  | ICA_GM_18 |  | 1.759 |  | 2.006 |  | 0.099 |  | 0.877 |  | 0.385 |  |
|  |  | ICA_GM_19 |  | -2.682 |  | 2.091 |  | -0.146 |  | -1.283 |  | 0.205 |  |
|  |  | ICA_GM_20 |  | -2.817 |  | 2.130 |  | -0.149 |  | -1.322 |  | 0.192 |  |
|  |  | ICA_GM_23 |  | -5.815 |  | 1.909 |  | -0.343 |  | -3.046 |  | 0.004 |  |
|  |  | ICA_GM_24 |  | 1.804 |  | 2.174 |  | 0.093 |  | 0.830 |  | 0.411 |  |
|  |  | ICA_GM_28 |  | -1.751 |  | 2.101 |  | -0.094 |  | -0.833 |  | 0.408 |  |
| 18 |  | (Intercept) |  | 0.819 |  | 0.392 |  |  |  | 2.092 |  | 0.041 |  |
|  |  | ICA_GM_01 |  | 1.108 |  | 1.621 |  | 0.077 |  | 0.684 |  | 0.497 |  |
|  |  | ICA_GM_04 |  | -1.235 |  | 1.702 |  | -0.080 |  | -0.726 |  | 0.471 |  |
|  |  | ICA_GM_05 |  | 6.281 |  | 1.921 |  | 0.365 |  | 3.270 |  | 0.002 |  |
|  |  | ICA_GM_10 |  | -1.443 |  | 1.927 |  | -0.083 |  | -0.749 |  | 0.457 |  |
|  |  | ICA_GM_14 |  | 2.277 |  | 2.103 |  | 0.120 |  | 1.083 |  | 0.284 |  |
|  |  | ICA_GM_18 |  | 1.838 |  | 1.988 |  | 0.104 |  | 0.924 |  | 0.360 |  |
|  |  | ICA_GM_19 |  | -2.707 |  | 2.077 |  | -0.147 |  | -1.304 |  | 0.198 |  |
|  |  | ICA_GM_20 |  | -2.810 |  | 2.116 |  | -0.149 |  | -1.328 |  | 0.190 |  |
|  |  | ICA_GM_23 |  | -5.706 |  | 1.886 |  | -0.337 |  | -3.025 |  | 0.004 |  |
|  |  | ICA_GM_24 |  | 1.793 |  | 2.160 |  | 0.092 |  | 0.830 |  | 0.410 |  |
|  |  | ICA_GM_28 |  | -1.846 |  | 2.080 |  | -0.099 |  | -0.888 |  | 0.379 |  |
| 19 |  | (Intercept) |  | 0.765 |  | 0.382 |  |  |  | 2.005 |  | 0.050 |  |
|  |  | ICA_GM_04 |  | -1.234 |  | 1.694 |  | -0.080 |  | -0.729 |  | 0.470 |  |
|  |  | ICA_GM_05 |  | 6.237 |  | 1.910 |  | 0.363 |  | 3.265 |  | 0.002 |  |
|  |  | ICA_GM_10 |  | -1.493 |  | 1.915 |  | -0.086 |  | -0.779 |  | 0.439 |  |
|  |  | ICA_GM_14 |  | 2.304 |  | 2.092 |  | 0.121 |  | 1.101 |  | 0.276 |  |
|  |  | ICA_GM_18 |  | 1.967 |  | 1.969 |  | 0.111 |  | 0.999 |  | 0.322 |  |
|  |  | ICA_GM_19 |  | -2.508 |  | 2.046 |  | -0.137 |  | -1.226 |  | 0.226 |  |
|  |  | ICA_GM_20 |  | -2.886 |  | 2.103 |  | -0.153 |  | -1.372 |  | 0.176 |  |
|  |  | ICA_GM_23 |  | -5.745 |  | 1.876 |  | -0.339 |  | -3.063 |  | 0.003 |  |
|  |  | ICA_GM_24 |  | 1.958 |  | 2.135 |  | 0.101 |  | 0.917 |  | 0.363 |  |
|  |  | ICA_GM_28 |  | -1.897 |  | 2.068 |  | -0.101 |  | -0.917 |  | 0.363 |  |
| 20 |  | (Intercept) |  | 0.733 |  | 0.377 |  |  |  | 1.943 |  | 0.057 |  |
|  |  | ICA_GM_05 |  | 6.208 |  | 1.901 |  | 0.361 |  | 3.265 |  | 0.002 |  |
|  |  | ICA_GM_10 |  | -1.549 |  | 1.906 |  | -0.089 |  | -0.813 |  | 0.420 |  |
|  |  | ICA_GM_14 |  | 2.316 |  | 2.082 |  | 0.122 |  | 1.112 |  | 0.271 |  |
|  |  | ICA_GM_18 |  | 1.975 |  | 1.960 |  | 0.112 |  | 1.008 |  | 0.318 |  |
|  |  | ICA_GM_19 |  | -2.518 |  | 2.037 |  | -0.137 |  | -1.236 |  | 0.222 |  |
|  |  | ICA_GM_20 |  | -2.889 |  | 2.094 |  | -0.153 |  | -1.380 |  | 0.173 |  |
|  |  | ICA_GM_23 |  | -5.848 |  | 1.863 |  | -0.345 |  | -3.140 |  | 0.003 |  |
|  |  | ICA_GM_24 |  | 1.981 |  | 2.126 |  | 0.102 |  | 0.932 |  | 0.356 |  |
|  |  | ICA_GM_28 |  | -1.836 |  | 2.057 |  | -0.098 |  | -0.892 |  | 0.376 |  |
| 21 |  | (Intercept) |  | 0.674 |  | 0.369 |  |  |  | 1.826 |  | 0.073 |  |
|  |  | ICA_GM_05 |  | 6.104 |  | 1.891 |  | 0.355 |  | 3.228 |  | 0.002 |  |
|  |  | ICA_GM_14 |  | 2.322 |  | 2.076 |  | 0.122 |  | 1.119 |  | 0.268 |  |
|  |  | ICA_GM_18 |  | 1.981 |  | 1.954 |  | 0.112 |  | 1.014 |  | 0.315 |  |
|  |  | ICA_GM_19 |  | -2.490 |  | 2.030 |  | -0.136 |  | -1.227 |  | 0.225 |  |
|  |  | ICA_GM_20 |  | -2.771 |  | 2.082 |  | -0.147 |  | -1.331 |  | 0.189 |  |
|  |  | ICA_GM_23 |  | -5.878 |  | 1.856 |  | -0.347 |  | -3.166 |  | 0.003 |  |
|  |  | ICA_GM_24 |  | 2.070 |  | 2.117 |  | 0.107 |  | 0.978 |  | 0.332 |  |
|  |  | ICA_GM_28 |  | -1.798 |  | 2.051 |  | -0.096 |  | -0.877 |  | 0.384 |  |
| 22 |  | (Intercept) |  | 0.654 |  | 0.368 |  |  |  | 1.779 |  | 0.081 |  |
|  |  | ICA_GM_05 |  | 6.176 |  | 1.885 |  | 0.359 |  | 3.276 |  | 0.002 |  |
|  |  | ICA_GM_14 |  | 2.355 |  | 2.071 |  | 0.124 |  | 1.137 |  | 0.260 |  |
|  |  | ICA_GM_18 |  | 2.014 |  | 1.950 |  | 0.114 |  | 1.033 |  | 0.306 |  |
|  |  | ICA_GM_19 |  | -2.648 |  | 2.018 |  | -0.144 |  | -1.312 |  | 0.195 |  |
|  |  | ICA_GM_20 |  | -2.783 |  | 2.078 |  | -0.147 |  | -1.339 |  | 0.186 |  |
|  |  | ICA_GM_23 |  | -5.695 |  | 1.841 |  | -0.336 |  | -3.094 |  | 0.003 |  |
|  |  | ICA_GM_24 |  | 2.059 |  | 2.112 |  | 0.106 |  | 0.975 |  | 0.334 |  |
| 23 |  | (Intercept) |  | 0.758 |  | 0.352 |  |  |  | 2.155 |  | 0.035 |  |
|  |  | ICA_GM_05 |  | 6.304 |  | 1.880 |  | 0.367 |  | 3.353 |  | 0.001 |  |
|  |  | ICA_GM_14 |  | 2.238 |  | 2.067 |  | 0.118 |  | 1.083 |  | 0.284 |  |
|  |  | ICA_GM_18 |  | 2.080 |  | 1.948 |  | 0.118 |  | 1.068 |  | 0.290 |  |
|  |  | ICA_GM_19 |  | -2.558 |  | 2.015 |  | -0.139 |  | -1.269 |  | 0.209 |  |
|  |  | ICA_GM_20 |  | -2.856 |  | 2.076 |  | -0.151 |  | -1.376 |  | 0.174 |  |
|  |  | ICA_GM_23 |  | -5.616 |  | 1.838 |  | -0.332 |  | -3.055 |  | 0.003 |  |
| 24 |  | (Intercept) |  | 0.882 |  | 0.332 |  |  |  | 2.654 |  | 0.010 |  |
|  |  | ICA_GM_05 |  | 6.287 |  | 1.882 |  | 0.366 |  | 3.340 |  | 0.001 |  |
|  |  | ICA_GM_14 |  | 2.245 |  | 2.070 |  | 0.118 |  | 1.085 |  | 0.283 |  |
|  |  | ICA_GM_19 |  | -2.193 |  | 1.988 |  | -0.119 |  | -1.103 |  | 0.275 |  |
|  |  | ICA_GM_20 |  | -2.803 |  | 2.077 |  | -0.148 |  | -1.349 |  | 0.182 |  |
|  |  | ICA_GM_23 |  | -5.790 |  | 1.833 |  | -0.342 |  | -3.159 |  | 0.003 |  |
| 25 |  | (Intercept) |  | 0.925 |  | 0.331 |  |  |  | 2.799 |  | 0.007 |  |
|  |  | ICA_GM_05 |  | 6.404 |  | 1.882 |  | 0.373 |  | 3.403 |  | 0.001 |  |
|  |  | ICA_GM_19 |  | -2.227 |  | 1.991 |  | -0.121 |  | -1.118 |  | 0.268 |  |
|  |  | ICA_GM_20 |  | -3.029 |  | 2.070 |  | -0.160 |  | -1.463 |  | 0.149 |  |
|  |  | ICA_GM_23 |  | -5.834 |  | 1.835 |  | -0.344 |  | -3.179 |  | 0.002 |  |
| 26 |  | (Intercept) |  | 0.743 |  | 0.288 |  |  |  | 2.578 |  | 0.012 |  |
|  |  | ICA_GM_05 |  | 6.347 |  | 1.885 |  | 0.369 |  | 3.367 |  | 0.001 |  |
|  |  | ICA_GM_20 |  | -3.065 |  | 2.074 |  | -0.162 |  | -1.478 |  | 0.145 |  |
|  |  | ICA_GM_23 |  | -5.771 |  | 1.838 |  | -0.341 |  | -3.139 |  | 0.003 |  |
| 27 |  | (Intercept) |  | 0.814 |  | 0.287 |  |  |  | 2.840 |  | 0.006 |  |
|  |  | ICA_GM_05 |  | 6.750 |  | 1.883 |  | 0.393 |  | 3.584 |  | < .001 |  |
|  |  | ICA_GM_23 |  | -5.736 |  | 1.856 |  | -0.339 |  | -3.091 |  | 0.003 |  |
|  | | | | | | | | | | | | | |

**WM**

| Model Summary - OCPD dia | | | | | | | | | |
| --- | --- | --- | --- | --- | --- | --- | --- | --- | --- |
| Model | | R | | R² | | Adjusted R² | | RMSE | |
| 1 |  | 0.586 |  | 0.343 |  | -0.183 |  | 0.547 |  |
| 2 |  | 0.586 |  | 0.343 |  | -0.150 |  | 0.539 |  |
| 3 |  | 0.586 |  | 0.343 |  | -0.119 |  | 0.532 |  |
| 4 |  | 0.586 |  | 0.343 |  | -0.090 |  | 0.525 |  |
| 5 |  | 0.585 |  | 0.343 |  | -0.062 |  | 0.518 |  |
| 6 |  | 0.585 |  | 0.343 |  | -0.036 |  | 0.512 |  |
| 7 |  | 0.585 |  | 0.342 |  | -0.011 |  | 0.506 |  |
| 8 |  | 0.585 |  | 0.342 |  | 0.013 |  | 0.500 |  |
| 9 |  | 0.584 |  | 0.342 |  | 0.035 |  | 0.494 |  |
| 10 |  | 0.584 |  | 0.341 |  | 0.056 |  | 0.489 |  |
| 11 |  | 0.583 |  | 0.340 |  | 0.076 |  | 0.484 |  |
| 12 |  | 0.582 |  | 0.339 |  | 0.094 |  | 0.479 |  |
| 13 |  | 0.581 |  | 0.338 |  | 0.112 |  | 0.474 |  |
| 14 |  | 0.580 |  | 0.337 |  | 0.129 |  | 0.469 |  |
| 15 |  | 0.578 |  | 0.334 |  | 0.144 |  | 0.465 |  |
| 16 |  | 0.575 |  | 0.330 |  | 0.156 |  | 0.462 |  |
| 17 |  | 0.570 |  | 0.325 |  | 0.166 |  | 0.459 |  |
| 18 |  | 0.564 |  | 0.318 |  | 0.174 |  | 0.457 |  |
| 19 |  | 0.553 |  | 0.306 |  | 0.175 |  | 0.457 |  |
| 20 |  | 0.541 |  | 0.293 |  | 0.175 |  | 0.457 |  |
| 21 |  | 0.526 |  | 0.276 |  | 0.171 |  | 0.458 |  |
| 22 |  | 0.512 |  | 0.262 |  | 0.170 |  | 0.458 |  |
| 23 |  | 0.495 |  | 0.245 |  | 0.165 |  | 0.460 |  |
| 24 |  | 0.474 |  | 0.224 |  | 0.157 |  | 0.462 |  |
| 25 |  | 0.450 |  | 0.202 |  | 0.148 |  | 0.464 |  |
| 26 |  | 0.419 |  | 0.175 |  | 0.134 |  | 0.468 |  |
| 27 |  | 0.386 |  | 0.149 |  | 0.121 |  | 0.472 |  |
|  | | | | | | | | | |

| ANOVA | | | | | | | | | | | | | |
| --- | --- | --- | --- | --- | --- | --- | --- | --- | --- | --- | --- | --- | --- |
| Model | |  | | Sum of Squares | | df | | Mean Square | | F | | p | |
| 1 |  | Regression |  | 5.465 |  | 28 |  | 0.195 |  | 0.652 |  | 0.876 |  |
|  |  | Residual |  | 10.473 |  | 35 |  | 0.299 |  |  |  |  |  |
|  |  | Total |  | 15.937 |  | 63 |  |  |  |  |  |  |  |
| 2 |  | Regression |  | 5.465 |  | 27 |  | 0.202 |  | 0.696 |  | 0.834 |  |
|  |  | Residual |  | 10.473 |  | 36 |  | 0.291 |  |  |  |  |  |
|  |  | Total |  | 15.938 |  | 63 |  |  |  |  |  |  |  |
| 3 |  | Regression |  | 5.464 |  | 26 |  | 0.210 |  | 0.742 |  | 0.784 |  |
|  |  | Residual |  | 10.473 |  | 37 |  | 0.283 |  |  |  |  |  |
|  |  | Total |  | 15.938 |  | 63 |  |  |  |  |  |  |  |
| 4 |  | Regression |  | 5.464 |  | 25 |  | 0.219 |  | 0.793 |  | 0.726 |  |
|  |  | Residual |  | 10.474 |  | 38 |  | 0.276 |  |  |  |  |  |
|  |  | Total |  | 15.937 |  | 63 |  |  |  |  |  |  |  |
| 5 |  | Regression |  | 5.463 |  | 24 |  | 0.228 |  | 0.847 |  | 0.661 |  |
|  |  | Residual |  | 10.475 |  | 39 |  | 0.269 |  |  |  |  |  |
|  |  | Total |  | 15.938 |  | 63 |  |  |  |  |  |  |  |
| 6 |  | Regression |  | 5.459 |  | 23 |  | 0.237 |  | 0.906 |  | 0.591 |  |
|  |  | Residual |  | 10.479 |  | 40 |  | 0.262 |  |  |  |  |  |
|  |  | Total |  | 15.938 |  | 63 |  |  |  |  |  |  |  |
| 7 |  | Regression |  | 5.456 |  | 22 |  | 0.248 |  | 0.970 |  | 0.517 |  |
|  |  | Residual |  | 10.481 |  | 41 |  | 0.256 |  |  |  |  |  |
|  |  | Total |  | 15.937 |  | 63 |  |  |  |  |  |  |  |
| 8 |  | Regression |  | 5.452 |  | 21 |  | 0.260 |  | 1.040 |  | 0.442 |  |
|  |  | Residual |  | 10.485 |  | 42 |  | 0.250 |  |  |  |  |  |
|  |  | Total |  | 15.938 |  | 63 |  |  |  |  |  |  |  |
| 9 |  | Regression |  | 5.444 |  | 20 |  | 0.272 |  | 1.115 |  | 0.370 |  |
|  |  | Residual |  | 10.493 |  | 43 |  | 0.244 |  |  |  |  |  |
|  |  | Total |  | 15.938 |  | 63 |  |  |  |  |  |  |  |
| 10 |  | Regression |  | 5.429 |  | 19 |  | 0.286 |  | 1.197 |  | 0.303 |  |
|  |  | Residual |  | 10.508 |  | 44 |  | 0.239 |  |  |  |  |  |
|  |  | Total |  | 15.938 |  | 63 |  |  |  |  |  |  |  |
| 11 |  | Regression |  | 5.416 |  | 18 |  | 0.301 |  | 1.287 |  | 0.242 |  |
|  |  | Residual |  | 10.522 |  | 45 |  | 0.234 |  |  |  |  |  |
|  |  | Total |  | 15.938 |  | 63 |  |  |  |  |  |  |  |
| 12 |  | Regression |  | 5.397 |  | 17 |  | 0.317 |  | 1.385 |  | 0.188 |  |
|  |  | Residual |  | 10.541 |  | 46 |  | 0.229 |  |  |  |  |  |
|  |  | Total |  | 15.937 |  | 63 |  |  |  |  |  |  |  |
| 13 |  | Regression |  | 5.384 |  | 16 |  | 0.336 |  | 1.499 |  | 0.141 |  |
|  |  | Residual |  | 10.554 |  | 47 |  | 0.225 |  |  |  |  |  |
|  |  | Total |  | 15.938 |  | 63 |  |  |  |  |  |  |  |
| 14 |  | Regression |  | 5.366 |  | 15 |  | 0.358 |  | 1.624 |  | 0.102 |  |
|  |  | Residual |  | 10.571 |  | 48 |  | 0.220 |  |  |  |  |  |
|  |  | Total |  | 15.938 |  | 63 |  |  |  |  |  |  |  |
| 15 |  | Regression |  | 5.322 |  | 14 |  | 0.380 |  | 1.754 |  | 0.074 |  |
|  |  | Residual |  | 10.616 |  | 49 |  | 0.217 |  |  |  |  |  |
|  |  | Total |  | 15.937 |  | 63 |  |  |  |  |  |  |  |
| 16 |  | Regression |  | 5.262 |  | 13 |  | 0.405 |  | 1.896 |  | 0.054 |  |
|  |  | Residual |  | 10.676 |  | 50 |  | 0.214 |  |  |  |  |  |
|  |  | Total |  | 15.937 |  | 63 |  |  |  |  |  |  |  |
| 17 |  | Regression |  | 5.173 |  | 12 |  | 0.431 |  | 2.042 |  | 0.039 |  |
|  |  | Residual |  | 10.764 |  | 51 |  | 0.211 |  |  |  |  |  |
|  |  | Total |  | 15.938 |  | 63 |  |  |  |  |  |  |  |
| 18 |  | Regression |  | 5.072 |  | 11 |  | 0.461 |  | 2.207 |  | 0.028 |  |
|  |  | Residual |  | 10.865 |  | 52 |  | 0.209 |  |  |  |  |  |
|  |  | Total |  | 15.938 |  | 63 |  |  |  |  |  |  |  |
| 19 |  | Regression |  | 4.874 |  | 10 |  | 0.487 |  | 2.335 |  | 0.023 |  |
|  |  | Residual |  | 11.063 |  | 53 |  | 0.209 |  |  |  |  |  |
|  |  | Total |  | 15.938 |  | 63 |  |  |  |  |  |  |  |
| 20 |  | Regression |  | 4.665 |  | 9 |  | 0.518 |  | 2.483 |  | 0.019 |  |
|  |  | Residual |  | 11.273 |  | 54 |  | 0.209 |  |  |  |  |  |
|  |  | Total |  | 15.938 |  | 63 |  |  |  |  |  |  |  |
| 21 |  | Regression |  | 4.402 |  | 8 |  | 0.550 |  | 2.623 |  | 0.017 |  |
|  |  | Residual |  | 11.536 |  | 55 |  | 0.210 |  |  |  |  |  |
|  |  | Total |  | 15.938 |  | 63 |  |  |  |  |  |  |  |
| 22 |  | Regression |  | 4.178 |  | 7 |  | 0.597 |  | 2.842 |  | 0.013 |  |
|  |  | Residual |  | 11.760 |  | 56 |  | 0.210 |  |  |  |  |  |
|  |  | Total |  | 15.938 |  | 63 |  |  |  |  |  |  |  |
| 23 |  | Regression |  | 3.899 |  | 6 |  | 0.650 |  | 3.077 |  | 0.011 |  |
|  |  | Residual |  | 12.039 |  | 57 |  | 0.211 |  |  |  |  |  |
|  |  | Total |  | 15.937 |  | 63 |  |  |  |  |  |  |  |
| 24 |  | Regression |  | 3.575 |  | 5 |  | 0.715 |  | 3.354 |  | 0.010 |  |
|  |  | Residual |  | 12.363 |  | 58 |  | 0.213 |  |  |  |  |  |
|  |  | Total |  | 15.937 |  | 63 |  |  |  |  |  |  |  |
| 25 |  | Regression |  | 3.221 |  | 4 |  | 0.805 |  | 3.737 |  | 0.009 |  |
|  |  | Residual |  | 12.716 |  | 59 |  | 0.216 |  |  |  |  |  |
|  |  | Total |  | 15.938 |  | 63 |  |  |  |  |  |  |  |
| 26 |  | Regression |  | 2.795 |  | 3 |  | 0.932 |  | 4.254 |  | 0.009 |  |
|  |  | Residual |  | 13.142 |  | 60 |  | 0.219 |  |  |  |  |  |
|  |  | Total |  | 15.937 |  | 63 |  |  |  |  |  |  |  |
| 27 |  | Regression |  | 2.371 |  | 2 |  | 1.186 |  | 5.331 |  | 0.007 |  |
|  |  | Residual |  | 13.566 |  | 61 |  | 0.222 |  |  |  |  |  |
|  |  | Total |  | 15.937 |  | 63 |  |  |  |  |  |  |  |
|  | | | | | | | | | | | | | |

| Coefficients | | | | | | | | | | | | | |
| --- | --- | --- | --- | --- | --- | --- | --- | --- | --- | --- | --- | --- | --- |
| Model | |  | | Unstandardized | | Standard Error | | Standardized | | t | | p | |
| 1 |  | (Intercept) |  | 11.105 |  | 57.774 |  |  |  | 0.192 |  | 0.849 |  |
|  |  | ICA_WM_1 |  | -9.559 |  | 51.494 |  | -0.276 |  | -0.186 |  | 0.854 |  |
|  |  | ICA_WM_2 |  | -1.444 |  | 5.425 |  | -0.092 |  | -0.266 |  | 0.792 |  |
|  |  | ICA_WM_3 |  | -0.094 |  | 2.190 |  | -0.006 |  | -0.043 |  | 0.966 |  |
|  |  | ICA_WM_4 |  | -1.601 |  | 7.001 |  | -0.140 |  | -0.229 |  | 0.820 |  |
|  |  | ICA_WM_5 |  | 0.297 |  | 2.122 |  | 0.022 |  | 0.140 |  | 0.889 |  |
|  |  | ICA_WM_6 |  | 2.262 |  | 2.454 |  | 0.161 |  | 0.922 |  | 0.363 |  |
|  |  | ICA_WM_7 |  | -3.359 |  | 5.136 |  | -0.199 |  | -0.654 |  | 0.517 |  |
|  |  | ICA_WM_8 |  | 1.735 |  | 4.337 |  | 0.096 |  | 0.400 |  | 0.692 |  |
|  |  | ICA_WM_9 |  | -5.484 |  | 13.874 |  | -0.337 |  | -0.395 |  | 0.695 |  |
|  |  | ICA_WM_10 |  | 2.470 |  | 4.075 |  | 0.149 |  | 0.606 |  | 0.548 |  |
|  |  | ICA_WM_11 |  | 1.363 |  | 4.022 |  | 0.081 |  | 0.339 |  | 0.737 |  |
|  |  | ICA_WM_12 |  | -1.061 |  | 4.234 |  | -0.062 |  | -0.251 |  | 0.804 |  |
|  |  | ICA_WM_13 |  | 0.077 |  | 2.748 |  | 0.005 |  | 0.028 |  | 0.978 |  |
|  |  | ICA_WM_14 |  | -0.471 |  | 6.695 |  | -0.028 |  | -0.070 |  | 0.944 |  |
|  |  | ICA_WM_15 |  | 1.110 |  | 3.470 |  | 0.060 |  | 0.320 |  | 0.751 |  |
|  |  | ICA_WM_16 |  | 2.724 |  | 2.906 |  | 0.171 |  | 0.937 |  | 0.355 |  |
|  |  | ICA_WM_17 |  | -1.191 |  | 8.503 |  | -0.064 |  | -0.140 |  | 0.889 |  |
|  |  | ICA_WM_18 |  | -1.666 |  | 4.359 |  | -0.095 |  | -0.382 |  | 0.705 |  |
|  |  | ICA_WM_19 |  | 2.049 |  | 2.404 |  | 0.122 |  | 0.852 |  | 0.400 |  |
|  |  | ICA_WM_20 |  | 0.065 |  | 2.961 |  | 0.004 |  | 0.022 |  | 0.983 |  |
|  |  | ICA_WM_21 |  | -1.705 |  | 3.526 |  | -0.097 |  | -0.484 |  | 0.632 |  |
|  |  | ICA_WM_22 |  | 0.137 |  | 2.500 |  | 0.008 |  | 0.055 |  | 0.957 |  |
|  |  | ICA_WM_23 |  | -0.431 |  | 4.256 |  | -0.025 |  | -0.101 |  | 0.920 |  |
|  |  | ICA_WM_24 |  | -2.059 |  | 2.722 |  | -0.121 |  | -0.757 |  | 0.454 |  |
|  |  | ICA_WM_25 |  | 6.183 |  | 2.598 |  | 0.338 |  | 2.380 |  | 0.023 |  |
|  |  | ICA_WM_26 |  | -1.784 |  | 10.271 |  | -0.106 |  | -0.174 |  | 0.863 |  |
|  |  | ICA_WM_27 |  | 1.300 |  | 2.777 |  | 0.072 |  | 0.468 |  | 0.643 |  |
|  |  | ICA_WM_28 |  | -0.889 |  | 3.488 |  | -0.050 |  | -0.255 |  | 0.800 |  |
| 2 |  | (Intercept) |  | 11.750 |  | 49.143 |  |  |  | 0.239 |  | 0.812 |  |
|  |  | ICA_WM_1 |  | -10.133 |  | 43.826 |  | -0.292 |  | -0.231 |  | 0.818 |  |
|  |  | ICA_WM_2 |  | -1.390 |  | 4.759 |  | -0.088 |  | -0.292 |  | 0.772 |  |
|  |  | ICA_WM_3 |  | -0.096 |  | 2.158 |  | -0.006 |  | -0.044 |  | 0.965 |  |
|  |  | ICA_WM_4 |  | -1.678 |  | 5.985 |  | -0.147 |  | -0.280 |  | 0.781 |  |
|  |  | ICA_WM_5 |  | 0.307 |  | 2.045 |  | 0.023 |  | 0.150 |  | 0.882 |  |
|  |  | ICA_WM_6 |  | 2.250 |  | 2.359 |  | 0.160 |  | 0.954 |  | 0.346 |  |
|  |  | ICA_WM_7 |  | -3.411 |  | 4.502 |  | -0.202 |  | -0.758 |  | 0.454 |  |
|  |  | ICA_WM_8 |  | 1.694 |  | 3.868 |  | 0.094 |  | 0.438 |  | 0.664 |  |
|  |  | ICA_WM_9 |  | -5.635 |  | 11.889 |  | -0.346 |  | -0.474 |  | 0.638 |  |
|  |  | ICA_WM_10 |  | 2.432 |  | 3.641 |  | 0.146 |  | 0.668 |  | 0.508 |  |
|  |  | ICA_WM_11 |  | 1.326 |  | 3.595 |  | 0.079 |  | 0.369 |  | 0.715 |  |
|  |  | ICA_WM_12 |  | -1.098 |  | 3.818 |  | -0.065 |  | -0.288 |  | 0.775 |  |
|  |  | ICA_WM_13 |  | 0.058 |  | 2.573 |  | 0.004 |  | 0.022 |  | 0.982 |  |
|  |  | ICA_WM_14 |  | -0.541 |  | 5.814 |  | -0.033 |  | -0.093 |  | 0.926 |  |
|  |  | ICA_WM_15 |  | 1.084 |  | 3.229 |  | 0.059 |  | 0.336 |  | 0.739 |  |
|  |  | ICA_WM_16 |  | 2.701 |  | 2.682 |  | 0.170 |  | 1.007 |  | 0.321 |  |
|  |  | ICA_WM_17 |  | -1.279 |  | 7.420 |  | -0.068 |  | -0.172 |  | 0.864 |  |
|  |  | ICA_WM_18 |  | -1.705 |  | 3.935 |  | -0.097 |  | -0.433 |  | 0.667 |  |
|  |  | ICA_WM_19 |  | 2.045 |  | 2.363 |  | 0.122 |  | 0.865 |  | 0.393 |  |
|  |  | ICA_WM_21 |  | -1.735 |  | 3.210 |  | -0.099 |  | -0.540 |  | 0.592 |  |
|  |  | ICA_WM_22 |  | 0.136 |  | 2.465 |  | 0.008 |  | 0.055 |  | 0.956 |  |
|  |  | ICA_WM_23 |  | -0.472 |  | 3.791 |  | -0.027 |  | -0.124 |  | 0.902 |  |
|  |  | ICA_WM_24 |  | -2.042 |  | 2.573 |  | -0.120 |  | -0.794 |  | 0.433 |  |
|  |  | ICA_WM_25 |  | 6.178 |  | 2.549 |  | 0.338 |  | 2.423 |  | 0.021 |  |
|  |  | ICA_WM_26 |  | -1.895 |  | 8.807 |  | -0.113 |  | -0.215 |  | 0.831 |  |
|  |  | ICA_WM_27 |  | 1.287 |  | 2.680 |  | 0.071 |  | 0.480 |  | 0.634 |  |
|  |  | ICA_WM_28 |  | -0.916 |  | 3.223 |  | -0.051 |  | -0.284 |  | 0.778 |  |
| 3 |  | (Intercept) |  | 12.309 |  | 41.771 |  |  |  | 0.295 |  | 0.770 |  |
|  |  | ICA_WM_1 |  | -10.628 |  | 37.322 |  | -0.306 |  | -0.285 |  | 0.777 |  |
|  |  | ICA_WM_2 |  | -1.342 |  | 4.204 |  | -0.085 |  | -0.319 |  | 0.751 |  |
|  |  | ICA_WM_3 |  | -0.097 |  | 2.128 |  | -0.006 |  | -0.046 |  | 0.964 |  |
|  |  | ICA_WM_4 |  | -1.744 |  | 5.131 |  | -0.152 |  | -0.340 |  | 0.736 |  |
|  |  | ICA_WM_5 |  | 0.319 |  | 1.950 |  | 0.024 |  | 0.163 |  | 0.871 |  |
|  |  | ICA_WM_6 |  | 2.240 |  | 2.283 |  | 0.159 |  | 0.981 |  | 0.333 |  |
|  |  | ICA_WM_7 |  | -3.453 |  | 4.022 |  | -0.204 |  | -0.859 |  | 0.396 |  |
|  |  | ICA_WM_8 |  | 1.659 |  | 3.484 |  | 0.092 |  | 0.476 |  | 0.637 |  |
|  |  | ICA_WM_9 |  | -5.770 |  | 10.118 |  | -0.354 |  | -0.570 |  | 0.572 |  |
|  |  | ICA_WM_10 |  | 2.398 |  | 3.267 |  | 0.144 |  | 0.734 |  | 0.468 |  |
|  |  | ICA_WM_11 |  | 1.296 |  | 3.302 |  | 0.077 |  | 0.393 |  | 0.697 |  |
|  |  | ICA_WM_12 |  | -1.134 |  | 3.422 |  | -0.067 |  | -0.331 |  | 0.742 |  |
|  |  | ICA_WM_14 |  | -0.602 |  | 5.062 |  | -0.036 |  | -0.119 |  | 0.906 |  |
|  |  | ICA_WM_15 |  | 1.059 |  | 2.984 |  | 0.057 |  | 0.355 |  | 0.725 |  |
|  |  | ICA_WM_16 |  | 2.681 |  | 2.498 |  | 0.169 |  | 1.073 |  | 0.290 |  |
|  |  | ICA_WM_17 |  | -1.353 |  | 6.546 |  | -0.072 |  | -0.207 |  | 0.837 |  |
|  |  | ICA_WM_18 |  | -1.738 |  | 3.597 |  | -0.099 |  | -0.483 |  | 0.632 |  |
|  |  | ICA_WM_19 |  | 2.044 |  | 2.330 |  | 0.122 |  | 0.877 |  | 0.386 |  |
|  |  | ICA_WM_21 |  | -1.759 |  | 2.988 |  | -0.100 |  | -0.589 |  | 0.560 |  |
|  |  | ICA_WM_22 |  | 0.135 |  | 2.430 |  | 0.008 |  | 0.055 |  | 0.956 |  |
|  |  | ICA_WM_23 |  | -0.506 |  | 3.423 |  | -0.029 |  | -0.148 |  | 0.883 |  |
|  |  | ICA_WM_24 |  | -2.029 |  | 2.470 |  | -0.120 |  | -0.821 |  | 0.417 |  |
|  |  | ICA_WM_25 |  | 6.179 |  | 2.514 |  | 0.338 |  | 2.458 |  | 0.019 |  |
|  |  | ICA_WM_26 |  | -1.991 |  | 7.611 |  | -0.118 |  | -0.262 |  | 0.795 |  |
|  |  | ICA_WM_27 |  | 1.273 |  | 2.566 |  | 0.070 |  | 0.496 |  | 0.623 |  |
|  |  | ICA_WM_28 |  | -0.942 |  | 2.958 |  | -0.053 |  | -0.318 |  | 0.752 |  |
| 4 |  | (Intercept) |  | 12.308 |  | 41.218 |  |  |  | 0.299 |  | 0.767 |  |
|  |  | ICA_WM_1 |  | -10.628 |  | 36.828 |  | -0.307 |  | -0.289 |  | 0.774 |  |
|  |  | ICA_WM_2 |  | -1.341 |  | 4.148 |  | -0.085 |  | -0.323 |  | 0.748 |  |
|  |  | ICA_WM_4 |  | -1.738 |  | 5.062 |  | -0.152 |  | -0.343 |  | 0.733 |  |
|  |  | ICA_WM_5 |  | 0.312 |  | 1.917 |  | 0.023 |  | 0.162 |  | 0.872 |  |
|  |  | ICA_WM_6 |  | 2.249 |  | 2.246 |  | 0.160 |  | 1.001 |  | 0.323 |  |
|  |  | ICA_WM_7 |  | -3.459 |  | 3.967 |  | -0.205 |  | -0.872 |  | 0.389 |  |
|  |  | ICA_WM_8 |  | 1.659 |  | 3.438 |  | 0.092 |  | 0.483 |  | 0.632 |  |
|  |  | ICA_WM_9 |  | -5.769 |  | 9.985 |  | -0.354 |  | -0.578 |  | 0.567 |  |
|  |  | ICA_WM_10 |  | 2.396 |  | 3.223 |  | 0.144 |  | 0.743 |  | 0.462 |  |
|  |  | ICA_WM_11 |  | 1.295 |  | 3.258 |  | 0.077 |  | 0.397 |  | 0.693 |  |
|  |  | ICA_WM_12 |  | -1.134 |  | 3.376 |  | -0.067 |  | -0.336 |  | 0.739 |  |
|  |  | ICA_WM_14 |  | -0.599 |  | 4.995 |  | -0.036 |  | -0.120 |  | 0.905 |  |
|  |  | ICA_WM_15 |  | 1.056 |  | 2.944 |  | 0.057 |  | 0.359 |  | 0.722 |  |
|  |  | ICA_WM_16 |  | 2.681 |  | 2.465 |  | 0.169 |  | 1.088 |  | 0.284 |  |
|  |  | ICA_WM_17 |  | -1.359 |  | 6.459 |  | -0.073 |  | -0.210 |  | 0.834 |  |
|  |  | ICA_WM_18 |  | -1.727 |  | 3.541 |  | -0.098 |  | -0.488 |  | 0.629 |  |
|  |  | ICA_WM_19 |  | 2.045 |  | 2.299 |  | 0.122 |  | 0.890 |  | 0.379 |  |
|  |  | ICA_WM_21 |  | -1.767 |  | 2.944 |  | -0.101 |  | -0.600 |  | 0.552 |  |
|  |  | ICA_WM_22 |  | 0.139 |  | 2.396 |  | 0.008 |  | 0.058 |  | 0.954 |  |
|  |  | ICA_WM_23 |  | -0.505 |  | 3.378 |  | -0.029 |  | -0.149 |  | 0.882 |  |
|  |  | ICA_WM_24 |  | -2.034 |  | 2.435 |  | -0.120 |  | -0.835 |  | 0.409 |  |
|  |  | ICA_WM_25 |  | 6.173 |  | 2.477 |  | 0.337 |  | 2.492 |  | 0.017 |  |
|  |  | ICA_WM_26 |  | -1.990 |  | 7.510 |  | -0.118 |  | -0.265 |  | 0.792 |  |
|  |  | ICA_WM_27 |  | 1.267 |  | 2.529 |  | 0.070 |  | 0.501 |  | 0.619 |  |
|  |  | ICA_WM_28 |  | -0.940 |  | 2.919 |  | -0.053 |  | -0.322 |  | 0.749 |  |
| 5 |  | (Intercept) |  | 12.315 |  | 40.688 |  |  |  | 0.303 |  | 0.764 |  |
|  |  | ICA_WM_1 |  | -10.634 |  | 36.355 |  | -0.307 |  | -0.293 |  | 0.771 |  |
|  |  | ICA_WM_2 |  | -1.346 |  | 4.094 |  | -0.085 |  | -0.329 |  | 0.744 |  |
|  |  | ICA_WM_4 |  | -1.747 |  | 4.995 |  | -0.153 |  | -0.350 |  | 0.728 |  |
|  |  | ICA_WM_5 |  | 0.310 |  | 1.893 |  | 0.023 |  | 0.164 |  | 0.871 |  |
|  |  | ICA_WM_6 |  | 2.251 |  | 2.216 |  | 0.160 |  | 1.016 |  | 0.316 |  |
|  |  | ICA_WM_7 |  | -3.457 |  | 3.916 |  | -0.205 |  | -0.883 |  | 0.383 |  |
|  |  | ICA_WM_8 |  | 1.655 |  | 3.393 |  | 0.092 |  | 0.488 |  | 0.629 |  |
|  |  | ICA_WM_9 |  | -5.775 |  | 9.856 |  | -0.355 |  | -0.586 |  | 0.561 |  |
|  |  | ICA_WM_10 |  | 2.404 |  | 3.179 |  | 0.145 |  | 0.756 |  | 0.454 |  |
|  |  | ICA_WM_11 |  | 1.295 |  | 3.216 |  | 0.077 |  | 0.403 |  | 0.690 |  |
|  |  | ICA_WM_12 |  | -1.142 |  | 3.330 |  | -0.067 |  | -0.343 |  | 0.734 |  |
|  |  | ICA_WM_14 |  | -0.603 |  | 4.930 |  | -0.036 |  | -0.122 |  | 0.903 |  |
|  |  | ICA_WM_15 |  | 1.049 |  | 2.904 |  | 0.057 |  | 0.361 |  | 0.720 |  |
|  |  | ICA_WM_16 |  | 2.681 |  | 2.433 |  | 0.169 |  | 1.102 |  | 0.277 |  |
|  |  | ICA_WM_17 |  | -1.365 |  | 6.375 |  | -0.073 |  | -0.214 |  | 0.832 |  |
|  |  | ICA_WM_18 |  | -1.725 |  | 3.496 |  | -0.098 |  | -0.493 |  | 0.624 |  |
|  |  | ICA_WM_19 |  | 2.042 |  | 2.269 |  | 0.122 |  | 0.900 |  | 0.374 |  |
|  |  | ICA_WM_21 |  | -1.763 |  | 2.905 |  | -0.101 |  | -0.607 |  | 0.547 |  |
|  |  | ICA_WM_23 |  | -0.503 |  | 3.334 |  | -0.029 |  | -0.151 |  | 0.881 |  |
|  |  | ICA_WM_24 |  | -2.034 |  | 2.404 |  | -0.120 |  | -0.846 |  | 0.403 |  |
|  |  | ICA_WM_25 |  | 6.181 |  | 2.441 |  | 0.338 |  | 2.532 |  | 0.015 |  |
|  |  | ICA_WM_26 |  | -1.994 |  | 7.413 |  | -0.119 |  | -0.269 |  | 0.789 |  |
|  |  | ICA_WM_27 |  | 1.273 |  | 2.495 |  | 0.070 |  | 0.510 |  | 0.613 |  |
|  |  | ICA_WM_28 |  | -0.931 |  | 2.878 |  | -0.052 |  | -0.324 |  | 0.748 |  |
| 6 |  | (Intercept) |  | 7.865 |  | 18.008 |  |  |  | 0.437 |  | 0.665 |  |
|  |  | ICA_WM_1 |  | -6.668 |  | 16.251 |  | -0.192 |  | -0.410 |  | 0.684 |  |
|  |  | ICA_WM_2 |  | -1.735 |  | 2.542 |  | -0.110 |  | -0.683 |  | 0.499 |  |
|  |  | ICA_WM_4 |  | -1.225 |  | 2.568 |  | -0.107 |  | -0.477 |  | 0.636 |  |
|  |  | ICA_WM_5 |  | 0.243 |  | 1.788 |  | 0.018 |  | 0.136 |  | 0.893 |  |
|  |  | ICA_WM_6 |  | 2.312 |  | 2.132 |  | 0.164 |  | 1.084 |  | 0.285 |  |
|  |  | ICA_WM_7 |  | -3.104 |  | 2.614 |  | -0.184 |  | -1.187 |  | 0.242 |  |
|  |  | ICA_WM_8 |  | 1.908 |  | 2.654 |  | 0.106 |  | 0.719 |  | 0.476 |  |
|  |  | ICA_WM_9 |  | -4.741 |  | 5.001 |  | -0.291 |  | -0.948 |  | 0.349 |  |
|  |  | ICA_WM_10 |  | 2.647 |  | 2.451 |  | 0.159 |  | 1.080 |  | 0.287 |  |
|  |  | ICA_WM_11 |  | 1.551 |  | 2.411 |  | 0.092 |  | 0.643 |  | 0.524 |  |
|  |  | ICA_WM_12 |  | -0.868 |  | 2.435 |  | -0.051 |  | -0.356 |  | 0.723 |  |
|  |  | ICA_WM_15 |  | 1.226 |  | 2.490 |  | 0.066 |  | 0.492 |  | 0.625 |  |
|  |  | ICA_WM_16 |  | 2.800 |  | 2.202 |  | 0.176 |  | 1.272 |  | 0.211 |  |
|  |  | ICA_WM_17 |  | -0.725 |  | 3.602 |  | -0.039 |  | -0.201 |  | 0.841 |  |
|  |  | ICA_WM_18 |  | -1.445 |  | 2.610 |  | -0.082 |  | -0.554 |  | 0.583 |  |
|  |  | ICA_WM_19 |  | 2.075 |  | 2.225 |  | 0.124 |  | 0.933 |  | 0.357 |  |
|  |  | ICA_WM_21 |  | -1.585 |  | 2.483 |  | -0.090 |  | -0.638 |  | 0.527 |  |
|  |  | ICA_WM_23 |  | -0.239 |  | 2.510 |  | -0.014 |  | -0.095 |  | 0.925 |  |
|  |  | ICA_WM_24 |  | -2.130 |  | 2.243 |  | -0.126 |  | -0.949 |  | 0.348 |  |
|  |  | ICA_WM_25 |  | 6.208 |  | 2.401 |  | 0.339 |  | 2.585 |  | 0.013 |  |
|  |  | ICA_WM_26 |  | -1.223 |  | 3.851 |  | -0.073 |  | -0.318 |  | 0.752 |  |
|  |  | ICA_WM_27 |  | 1.328 |  | 2.424 |  | 0.073 |  | 0.548 |  | 0.587 |  |
|  |  | ICA_WM_28 |  | -0.756 |  | 2.464 |  | -0.042 |  | -0.307 |  | 0.761 |  |
| 7 |  | (Intercept) |  | 7.136 |  | 16.102 |  |  |  | 0.443 |  | 0.660 |  |
|  |  | ICA_WM_1 |  | -6.025 |  | 14.598 |  | -0.174 |  | -0.413 |  | 0.682 |  |
|  |  | ICA_WM_2 |  | -1.797 |  | 2.427 |  | -0.114 |  | -0.740 |  | 0.463 |  |
|  |  | ICA_WM_4 |  | -1.152 |  | 2.421 |  | -0.101 |  | -0.476 |  | 0.637 |  |
|  |  | ICA_WM_5 |  | 0.233 |  | 1.763 |  | 0.017 |  | 0.132 |  | 0.896 |  |
|  |  | ICA_WM_6 |  | 2.319 |  | 2.105 |  | 0.165 |  | 1.102 |  | 0.277 |  |
|  |  | ICA_WM_7 |  | -3.057 |  | 2.536 |  | -0.181 |  | -1.206 |  | 0.235 |  |
|  |  | ICA_WM_8 |  | 1.948 |  | 2.590 |  | 0.108 |  | 0.752 |  | 0.456 |  |
|  |  | ICA_WM_9 |  | -4.571 |  | 4.613 |  | -0.281 |  | -0.991 |  | 0.328 |  |
|  |  | ICA_WM_10 |  | 2.701 |  | 2.357 |  | 0.163 |  | 1.146 |  | 0.259 |  |
|  |  | ICA_WM_11 |  | 1.595 |  | 2.338 |  | 0.095 |  | 0.682 |  | 0.499 |  |
|  |  | ICA_WM_12 |  | -0.824 |  | 2.362 |  | -0.049 |  | -0.349 |  | 0.729 |  |
|  |  | ICA_WM_15 |  | 1.249 |  | 2.447 |  | 0.068 |  | 0.510 |  | 0.612 |  |
|  |  | ICA_WM_16 |  | 2.814 |  | 2.170 |  | 0.177 |  | 1.297 |  | 0.202 |  |
|  |  | ICA_WM_17 |  | -0.623 |  | 3.395 |  | -0.033 |  | -0.184 |  | 0.855 |  |
|  |  | ICA_WM_18 |  | -1.396 |  | 2.528 |  | -0.080 |  | -0.552 |  | 0.584 |  |
|  |  | ICA_WM_19 |  | 2.073 |  | 2.198 |  | 0.123 |  | 0.943 |  | 0.351 |  |
|  |  | ICA_WM_21 |  | -1.551 |  | 2.428 |  | -0.089 |  | -0.639 |  | 0.526 |  |
|  |  | ICA_WM_24 |  | -2.146 |  | 2.210 |  | -0.127 |  | -0.971 |  | 0.337 |  |
|  |  | ICA_WM_25 |  | 6.217 |  | 2.370 |  | 0.340 |  | 2.623 |  | 0.012 |  |
|  |  | ICA_WM_26 |  | -1.101 |  | 3.588 |  | -0.066 |  | -0.307 |  | 0.760 |  |
|  |  | ICA_WM_27 |  | 1.337 |  | 2.393 |  | 0.074 |  | 0.559 |  | 0.579 |  |
|  |  | ICA_WM_28 |  | -0.737 |  | 2.426 |  | -0.041 |  | -0.304 |  | 0.763 |  |
| 8 |  | (Intercept) |  | 6.809 |  | 15.723 |  |  |  | 0.433 |  | 0.667 |  |
|  |  | ICA_WM_1 |  | -5.715 |  | 14.239 |  | -0.165 |  | -0.401 |  | 0.690 |  |
|  |  | ICA_WM_2 |  | -1.818 |  | 2.394 |  | -0.115 |  | -0.760 |  | 0.452 |  |
|  |  | ICA_WM_4 |  | -1.115 |  | 2.376 |  | -0.097 |  | -0.469 |  | 0.641 |  |
|  |  | ICA_WM_6 |  | 2.322 |  | 2.080 |  | 0.165 |  | 1.116 |  | 0.271 |  |
|  |  | ICA_WM_7 |  | -3.038 |  | 2.502 |  | -0.180 |  | -1.214 |  | 0.231 |  |
|  |  | ICA_WM_8 |  | 1.953 |  | 2.559 |  | 0.108 |  | 0.763 |  | 0.450 |  |
|  |  | ICA_WM_9 |  | -4.505 |  | 4.532 |  | -0.277 |  | -0.994 |  | 0.326 |  |
|  |  | ICA_WM_10 |  | 2.739 |  | 2.311 |  | 0.165 |  | 1.185 |  | 0.243 |  |
|  |  | ICA_WM_11 |  | 1.618 |  | 2.304 |  | 0.096 |  | 0.702 |  | 0.486 |  |
|  |  | ICA_WM_12 |  | -0.823 |  | 2.334 |  | -0.048 |  | -0.353 |  | 0.726 |  |
|  |  | ICA_WM_15 |  | 1.269 |  | 2.414 |  | 0.069 |  | 0.525 |  | 0.602 |  |
|  |  | ICA_WM_16 |  | 2.829 |  | 2.142 |  | 0.178 |  | 1.321 |  | 0.194 |  |
|  |  | ICA_WM_17 |  | -0.594 |  | 3.348 |  | -0.032 |  | -0.177 |  | 0.860 |  |
|  |  | ICA_WM_18 |  | -1.401 |  | 2.498 |  | -0.080 |  | -0.561 |  | 0.578 |  |
|  |  | ICA_WM_19 |  | 2.087 |  | 2.169 |  | 0.124 |  | 0.962 |  | 0.342 |  |
|  |  | ICA_WM_21 |  | -1.550 |  | 2.399 |  | -0.088 |  | -0.646 |  | 0.522 |  |
|  |  | ICA_WM_24 |  | -2.135 |  | 2.182 |  | -0.126 |  | -0.979 |  | 0.333 |  |
|  |  | ICA_WM_25 |  | 6.213 |  | 2.342 |  | 0.340 |  | 2.652 |  | 0.011 |  |
|  |  | ICA_WM_26 |  | -1.060 |  | 3.532 |  | -0.063 |  | -0.300 |  | 0.766 |  |
|  |  | ICA_WM_27 |  | 1.337 |  | 2.364 |  | 0.074 |  | 0.566 |  | 0.575 |  |
|  |  | ICA_WM_28 |  | -0.701 |  | 2.382 |  | -0.039 |  | -0.294 |  | 0.770 |  |
| 9 |  | (Intercept) |  | 4.805 |  | 10.813 |  |  |  | 0.444 |  | 0.659 |  |
|  |  | ICA_WM_1 |  | -3.936 |  | 9.993 |  | -0.114 |  | -0.394 |  | 0.696 |  |
|  |  | ICA_WM_2 |  | -1.973 |  | 2.203 |  | -0.125 |  | -0.896 |  | 0.375 |  |
|  |  | ICA_WM_4 |  | -0.882 |  | 1.958 |  | -0.077 |  | -0.450 |  | 0.655 |  |
|  |  | ICA_WM_6 |  | 2.379 |  | 2.032 |  | 0.169 |  | 1.171 |  | 0.248 |  |
|  |  | ICA_WM_7 |  | -2.899 |  | 2.349 |  | -0.172 |  | -1.234 |  | 0.224 |  |
|  |  | ICA_WM_8 |  | 2.082 |  | 2.425 |  | 0.116 |  | 0.858 |  | 0.395 |  |
|  |  | ICA_WM_9 |  | -4.016 |  | 3.557 |  | -0.247 |  | -1.129 |  | 0.265 |  |
|  |  | ICA_WM_10 |  | 2.857 |  | 2.190 |  | 0.172 |  | 1.305 |  | 0.199 |  |
|  |  | ICA_WM_11 |  | 1.730 |  | 2.191 |  | 0.103 |  | 0.790 |  | 0.434 |  |
|  |  | ICA_WM_12 |  | -0.711 |  | 2.221 |  | -0.042 |  | -0.320 |  | 0.750 |  |
|  |  | ICA_WM_15 |  | 1.353 |  | 2.340 |  | 0.073 |  | 0.578 |  | 0.566 |  |
|  |  | ICA_WM_16 |  | 2.897 |  | 2.084 |  | 0.182 |  | 1.390 |  | 0.172 |  |
|  |  | ICA_WM_18 |  | -1.286 |  | 2.386 |  | -0.073 |  | -0.539 |  | 0.593 |  |
|  |  | ICA_WM_19 |  | 2.095 |  | 2.144 |  | 0.125 |  | 0.977 |  | 0.334 |  |
|  |  | ICA_WM_21 |  | -1.444 |  | 2.297 |  | -0.082 |  | -0.629 |  | 0.533 |  |
|  |  | ICA_WM_24 |  | -2.192 |  | 2.134 |  | -0.129 |  | -1.027 |  | 0.310 |  |
|  |  | ICA_WM_25 |  | 6.238 |  | 2.311 |  | 0.341 |  | 2.699 |  | 0.010 |  |
|  |  | ICA_WM_26 |  | -0.716 |  | 2.917 |  | -0.043 |  | -0.245 |  | 0.807 |  |
|  |  | ICA_WM_27 |  | 1.377 |  | 2.327 |  | 0.076 |  | 0.592 |  | 0.557 |  |
|  |  | ICA_WM_28 |  | -0.613 |  | 2.304 |  | -0.034 |  | -0.266 |  | 0.791 |  |
| 10 |  | (Intercept) |  | 2.960 |  | 7.685 |  |  |  | 0.385 |  | 0.702 |  |
|  |  | ICA_WM_1 |  | -2.293 |  | 7.338 |  | -0.066 |  | -0.313 |  | 0.756 |  |
|  |  | ICA_WM_2 |  | -2.121 |  | 2.096 |  | -0.135 |  | -1.012 |  | 0.317 |  |
|  |  | ICA_WM_4 |  | -0.633 |  | 1.656 |  | -0.055 |  | -0.382 |  | 0.704 |  |
|  |  | ICA_WM_6 |  | 2.470 |  | 1.976 |  | 0.176 |  | 1.250 |  | 0.218 |  |
|  |  | ICA_WM_7 |  | -2.785 |  | 2.278 |  | -0.165 |  | -1.222 |  | 0.228 |  |
|  |  | ICA_WM_8 |  | 2.185 |  | 2.363 |  | 0.121 |  | 0.925 |  | 0.360 |  |
|  |  | ICA_WM_9 |  | -3.544 |  | 2.959 |  | -0.218 |  | -1.198 |  | 0.237 |  |
|  |  | ICA_WM_10 |  | 2.951 |  | 2.133 |  | 0.178 |  | 1.384 |  | 0.173 |  |
|  |  | ICA_WM_11 |  | 1.802 |  | 2.147 |  | 0.107 |  | 0.839 |  | 0.406 |  |
|  |  | ICA_WM_12 |  | -0.594 |  | 2.146 |  | -0.035 |  | -0.277 |  | 0.783 |  |
|  |  | ICA_WM_15 |  | 1.408 |  | 2.304 |  | 0.076 |  | 0.611 |  | 0.544 |  |
|  |  | ICA_WM_16 |  | 2.935 |  | 2.055 |  | 0.185 |  | 1.428 |  | 0.160 |  |
|  |  | ICA_WM_18 |  | -1.138 |  | 2.284 |  | -0.065 |  | -0.498 |  | 0.621 |  |
|  |  | ICA_WM_19 |  | 2.098 |  | 2.121 |  | 0.125 |  | 0.989 |  | 0.328 |  |
|  |  | ICA_WM_21 |  | -1.378 |  | 2.256 |  | -0.079 |  | -0.611 |  | 0.545 |  |
|  |  | ICA_WM_24 |  | -2.213 |  | 2.110 |  | -0.131 |  | -1.049 |  | 0.300 |  |
|  |  | ICA_WM_25 |  | 6.255 |  | 2.286 |  | 0.342 |  | 2.737 |  | 0.009 |  |
|  |  | ICA_WM_27 |  | 1.393 |  | 2.301 |  | 0.077 |  | 0.605 |  | 0.548 |  |
|  |  | ICA_WM_28 |  | -0.545 |  | 2.262 |  | -0.031 |  | -0.241 |  | 0.811 |  |
| 11 |  | (Intercept) |  | 2.731 |  | 7.546 |  |  |  | 0.362 |  | 0.719 |  |
|  |  | ICA_WM_1 |  | -2.102 |  | 7.218 |  | -0.061 |  | -0.291 |  | 0.772 |  |
|  |  | ICA_WM_2 |  | -2.139 |  | 2.073 |  | -0.136 |  | -1.032 |  | 0.308 |  |
|  |  | ICA_WM_4 |  | -0.600 |  | 1.633 |  | -0.052 |  | -0.367 |  | 0.715 |  |
|  |  | ICA_WM_6 |  | 2.416 |  | 1.943 |  | 0.172 |  | 1.244 |  | 0.220 |  |
|  |  | ICA_WM_7 |  | -2.750 |  | 2.250 |  | -0.163 |  | -1.223 |  | 0.228 |  |
|  |  | ICA_WM_8 |  | 2.185 |  | 2.338 |  | 0.121 |  | 0.935 |  | 0.355 |  |
|  |  | ICA_WM_9 |  | -3.489 |  | 2.919 |  | -0.214 |  | -1.195 |  | 0.238 |  |
|  |  | ICA_WM_10 |  | 3.037 |  | 2.080 |  | 0.183 |  | 1.460 |  | 0.151 |  |
|  |  | ICA_WM_11 |  | 1.783 |  | 2.123 |  | 0.106 |  | 0.840 |  | 0.405 |  |
|  |  | ICA_WM_12 |  | -0.606 |  | 2.123 |  | -0.036 |  | -0.285 |  | 0.777 |  |
|  |  | ICA_WM_15 |  | 1.428 |  | 2.279 |  | 0.077 |  | 0.627 |  | 0.534 |  |
|  |  | ICA_WM_16 |  | 2.933 |  | 2.034 |  | 0.184 |  | 1.442 |  | 0.156 |  |
|  |  | ICA_WM_18 |  | -1.099 |  | 2.254 |  | -0.063 |  | -0.487 |  | 0.628 |  |
|  |  | ICA_WM_19 |  | 2.118 |  | 2.097 |  | 0.126 |  | 1.010 |  | 0.318 |  |
|  |  | ICA_WM_21 |  | -1.350 |  | 2.230 |  | -0.077 |  | -0.605 |  | 0.548 |  |
|  |  | ICA_WM_24 |  | -2.195 |  | 2.086 |  | -0.130 |  | -1.052 |  | 0.298 |  |
|  |  | ICA_WM_25 |  | 6.254 |  | 2.262 |  | 0.342 |  | 2.765 |  | 0.008 |  |
|  |  | ICA_WM_27 |  | 1.370 |  | 2.275 |  | 0.075 |  | 0.602 |  | 0.550 |  |
| 12 |  | (Intercept) |  | 2.219 |  | 7.256 |  |  |  | 0.306 |  | 0.761 |  |
|  |  | ICA_WM_1 |  | -1.649 |  | 6.970 |  | -0.048 |  | -0.237 |  | 0.814 |  |
|  |  | ICA_WM_2 |  | -2.175 |  | 2.048 |  | -0.138 |  | -1.062 |  | 0.294 |  |
|  |  | ICA_WM_4 |  | -0.571 |  | 1.614 |  | -0.050 |  | -0.354 |  | 0.725 |  |
|  |  | ICA_WM_6 |  | 2.441 |  | 1.921 |  | 0.173 |  | 1.270 |  | 0.210 |  |
|  |  | ICA_WM_7 |  | -2.716 |  | 2.224 |  | -0.161 |  | -1.221 |  | 0.228 |  |
|  |  | ICA_WM_8 |  | 2.246 |  | 2.305 |  | 0.125 |  | 0.974 |  | 0.335 |  |
|  |  | ICA_WM_9 |  | -3.381 |  | 2.865 |  | -0.208 |  | -1.180 |  | 0.244 |  |
|  |  | ICA_WM_10 |  | 3.064 |  | 2.057 |  | 0.184 |  | 1.489 |  | 0.143 |  |
|  |  | ICA_WM_11 |  | 1.833 |  | 2.095 |  | 0.109 |  | 0.875 |  | 0.386 |  |
|  |  | ICA_WM_15 |  | 1.408 |  | 2.255 |  | 0.076 |  | 0.624 |  | 0.536 |  |
|  |  | ICA_WM_16 |  | 2.961 |  | 2.011 |  | 0.186 |  | 1.473 |  | 0.148 |  |
|  |  | ICA_WM_18 |  | -1.076 |  | 2.230 |  | -0.061 |  | -0.483 |  | 0.632 |  |
|  |  | ICA_WM_19 |  | 2.126 |  | 2.076 |  | 0.127 |  | 1.024 |  | 0.311 |  |
|  |  | ICA_WM_21 |  | -1.306 |  | 2.202 |  | -0.075 |  | -0.593 |  | 0.556 |  |
|  |  | ICA_WM_24 |  | -2.204 |  | 2.065 |  | -0.130 |  | -1.068 |  | 0.291 |  |
|  |  | ICA_WM_25 |  | 6.236 |  | 2.238 |  | 0.341 |  | 2.786 |  | 0.008 |  |
|  |  | ICA_WM_27 |  | 1.386 |  | 2.252 |  | 0.076 |  | 0.615 |  | 0.541 |  |
| 13 |  | (Intercept) |  | 0.513 |  | 0.775 |  |  |  | 0.662 |  | 0.511 |  |
|  |  | ICA_WM_2 |  | -2.313 |  | 1.944 |  | -0.147 |  | -1.189 |  | 0.240 |  |
|  |  | ICA_WM_4 |  | -0.403 |  | 1.433 |  | -0.035 |  | -0.281 |  | 0.780 |  |
|  |  | ICA_WM_6 |  | 2.510 |  | 1.879 |  | 0.178 |  | 1.336 |  | 0.188 |  |
|  |  | ICA_WM_7 |  | -2.522 |  | 2.047 |  | -0.149 |  | -1.232 |  | 0.224 |  |
|  |  | ICA_WM_8 |  | 2.380 |  | 2.211 |  | 0.132 |  | 1.076 |  | 0.287 |  |
|  |  | ICA_WM_9 |  | -2.903 |  | 2.013 |  | -0.178 |  | -1.442 |  | 0.156 |  |
|  |  | ICA_WM_10 |  | 3.167 |  | 1.990 |  | 0.191 |  | 1.591 |  | 0.118 |  |
|  |  | ICA_WM_11 |  | 1.941 |  | 2.023 |  | 0.115 |  | 0.960 |  | 0.342 |  |
|  |  | ICA_WM_15 |  | 1.486 |  | 2.208 |  | 0.081 |  | 0.673 |  | 0.504 |  |
|  |  | ICA_WM_16 |  | 3.063 |  | 1.945 |  | 0.193 |  | 1.575 |  | 0.122 |  |
|  |  | ICA_WM_18 |  | -0.921 |  | 2.109 |  | -0.053 |  | -0.437 |  | 0.664 |  |
|  |  | ICA_WM_19 |  | 2.161 |  | 2.050 |  | 0.129 |  | 1.055 |  | 0.297 |  |
|  |  | ICA_WM_21 |  | -1.201 |  | 2.135 |  | -0.069 |  | -0.563 |  | 0.576 |  |
|  |  | ICA_WM_24 |  | -2.226 |  | 2.042 |  | -0.131 |  | -1.090 |  | 0.281 |  |
|  |  | ICA_WM_25 |  | 6.272 |  | 2.210 |  | 0.343 |  | 2.838 |  | 0.007 |  |
|  |  | ICA_WM_27 |  | 1.436 |  | 2.219 |  | 0.079 |  | 0.647 |  | 0.521 |  |
| 14 |  | (Intercept) |  | 0.415 |  | 0.685 |  |  |  | 0.605 |  | 0.548 |  |
|  |  | ICA_WM_2 |  | -2.326 |  | 1.925 |  | -0.148 |  | -1.208 |  | 0.233 |  |
|  |  | ICA_WM_6 |  | 2.585 |  | 1.843 |  | 0.184 |  | 1.403 |  | 0.167 |  |
|  |  | ICA_WM_7 |  | -2.487 |  | 2.024 |  | -0.147 |  | -1.229 |  | 0.225 |  |
|  |  | ICA_WM_8 |  | 2.421 |  | 2.185 |  | 0.134 |  | 1.108 |  | 0.273 |  |
|  |  | ICA_WM_9 |  | -2.798 |  | 1.959 |  | -0.172 |  | -1.428 |  | 0.160 |  |
|  |  | ICA_WM_10 |  | 3.179 |  | 1.971 |  | 0.191 |  | 1.613 |  | 0.113 |  |
|  |  | ICA_WM_11 |  | 1.936 |  | 2.004 |  | 0.115 |  | 0.966 |  | 0.339 |  |
|  |  | ICA_WM_15 |  | 1.437 |  | 2.180 |  | 0.078 |  | 0.659 |  | 0.513 |  |
|  |  | ICA_WM_16 |  | 3.085 |  | 1.924 |  | 0.194 |  | 1.603 |  | 0.116 |  |
|  |  | ICA_WM_18 |  | -0.938 |  | 2.087 |  | -0.054 |  | -0.449 |  | 0.655 |  |
|  |  | ICA_WM_19 |  | 2.167 |  | 2.030 |  | 0.129 |  | 1.068 |  | 0.291 |  |
|  |  | ICA_WM_21 |  | -1.131 |  | 2.100 |  | -0.065 |  | -0.538 |  | 0.593 |  |
|  |  | ICA_WM_24 |  | -2.236 |  | 2.022 |  | -0.132 |  | -1.106 |  | 0.274 |  |
|  |  | ICA_WM_25 |  | 6.215 |  | 2.180 |  | 0.340 |  | 2.851 |  | 0.006 |  |
|  |  | ICA_WM_27 |  | 1.495 |  | 2.187 |  | 0.082 |  | 0.684 |  | 0.498 |  |
| 15 |  | (Intercept) |  | 0.343 |  | 0.661 |  |  |  | 0.519 |  | 0.606 |  |
|  |  | ICA_WM_2 |  | -2.289 |  | 1.907 |  | -0.145 |  | -1.200 |  | 0.236 |  |
|  |  | ICA_WM_6 |  | 2.548 |  | 1.826 |  | 0.181 |  | 1.396 |  | 0.169 |  |
|  |  | ICA_WM_7 |  | -2.446 |  | 2.005 |  | -0.145 |  | -1.220 |  | 0.228 |  |
|  |  | ICA_WM_8 |  | 2.341 |  | 2.160 |  | 0.130 |  | 1.084 |  | 0.284 |  |
|  |  | ICA_WM_9 |  | -2.770 |  | 1.942 |  | -0.170 |  | -1.426 |  | 0.160 |  |
|  |  | ICA_WM_10 |  | 3.170 |  | 1.954 |  | 0.191 |  | 1.622 |  | 0.111 |  |
|  |  | ICA_WM_11 |  | 1.860 |  | 1.980 |  | 0.110 |  | 0.939 |  | 0.352 |  |
|  |  | ICA_WM_15 |  | 1.449 |  | 2.162 |  | 0.079 |  | 0.670 |  | 0.506 |  |
|  |  | ICA_WM_16 |  | 3.065 |  | 1.908 |  | 0.193 |  | 1.606 |  | 0.115 |  |
|  |  | ICA_WM_19 |  | 2.142 |  | 2.013 |  | 0.128 |  | 1.064 |  | 0.292 |  |
|  |  | ICA_WM_21 |  | -1.093 |  | 2.081 |  | -0.062 |  | -0.525 |  | 0.602 |  |
|  |  | ICA_WM_24 |  | -2.224 |  | 2.005 |  | -0.131 |  | -1.109 |  | 0.273 |  |
|  |  | ICA_WM_25 |  | 6.182 |  | 2.161 |  | 0.338 |  | 2.861 |  | 0.006 |  |
|  |  | ICA_WM_27 |  | 1.452 |  | 2.168 |  | 0.080 |  | 0.670 |  | 0.506 |  |
| 16 |  | (Intercept) |  | 0.288 |  | 0.648 |  |  |  | 0.445 |  | 0.658 |  |
|  |  | ICA_WM_2 |  | -2.310 |  | 1.893 |  | -0.147 |  | -1.220 |  | 0.228 |  |
|  |  | ICA_WM_6 |  | 2.663 |  | 1.799 |  | 0.189 |  | 1.480 |  | 0.145 |  |
|  |  | ICA_WM_7 |  | -2.517 |  | 1.986 |  | -0.149 |  | -1.267 |  | 0.211 |  |
|  |  | ICA_WM_8 |  | 2.283 |  | 2.142 |  | 0.127 |  | 1.066 |  | 0.291 |  |
|  |  | ICA_WM_9 |  | -2.740 |  | 1.927 |  | -0.168 |  | -1.422 |  | 0.161 |  |
|  |  | ICA_WM_10 |  | 3.146 |  | 1.940 |  | 0.189 |  | 1.622 |  | 0.111 |  |
|  |  | ICA_WM_11 |  | 1.852 |  | 1.966 |  | 0.110 |  | 0.942 |  | 0.351 |  |
|  |  | ICA_WM_15 |  | 1.381 |  | 2.142 |  | 0.075 |  | 0.645 |  | 0.522 |  |
|  |  | ICA_WM_16 |  | 3.099 |  | 1.893 |  | 0.195 |  | 1.637 |  | 0.108 |  |
|  |  | ICA_WM_19 |  | 2.152 |  | 1.998 |  | 0.128 |  | 1.077 |  | 0.287 |  |
|  |  | ICA_WM_24 |  | -2.293 |  | 1.987 |  | -0.135 |  | -1.154 |  | 0.254 |  |
|  |  | ICA_WM_25 |  | 6.177 |  | 2.145 |  | 0.338 |  | 2.880 |  | 0.006 |  |
|  |  | ICA_WM_27 |  | 1.468 |  | 2.152 |  | 0.081 |  | 0.682 |  | 0.498 |  |
| 17 |  | (Intercept) |  | 0.408 |  | 0.617 |  |  |  | 0.662 |  | 0.511 |  |
|  |  | ICA_WM_2 |  | -2.284 |  | 1.882 |  | -0.145 |  | -1.214 |  | 0.230 |  |
|  |  | ICA_WM_6 |  | 2.705 |  | 1.788 |  | 0.192 |  | 1.513 |  | 0.136 |  |
|  |  | ICA_WM_7 |  | -2.480 |  | 1.974 |  | -0.147 |  | -1.257 |  | 0.215 |  |
|  |  | ICA_WM_8 |  | 2.295 |  | 2.129 |  | 0.127 |  | 1.078 |  | 0.286 |  |
|  |  | ICA_WM_9 |  | -2.804 |  | 1.913 |  | -0.172 |  | -1.465 |  | 0.149 |  |
|  |  | ICA_WM_10 |  | 3.160 |  | 1.928 |  | 0.190 |  | 1.639 |  | 0.107 |  |
|  |  | ICA_WM_11 |  | 1.845 |  | 1.955 |  | 0.109 |  | 0.944 |  | 0.350 |  |
|  |  | ICA_WM_16 |  | 3.098 |  | 1.882 |  | 0.195 |  | 1.646 |  | 0.106 |  |
|  |  | ICA_WM_19 |  | 2.097 |  | 1.985 |  | 0.125 |  | 1.057 |  | 0.296 |  |
|  |  | ICA_WM_24 |  | -2.314 |  | 1.975 |  | -0.137 |  | -1.172 |  | 0.247 |  |
|  |  | ICA_WM_25 |  | 6.190 |  | 2.133 |  | 0.338 |  | 2.903 |  | 0.005 |  |
|  |  | ICA_WM_27 |  | 1.478 |  | 2.139 |  | 0.081 |  | 0.691 |  | 0.493 |  |
| 18 |  | (Intercept) |  | 0.442 |  | 0.612 |  |  |  | 0.722 |  | 0.474 |  |
|  |  | ICA_WM_2 |  | -2.230 |  | 1.871 |  | -0.142 |  | -1.192 |  | 0.239 |  |
|  |  | ICA_WM_6 |  | 2.498 |  | 1.754 |  | 0.177 |  | 1.425 |  | 0.160 |  |
|  |  | ICA_WM_7 |  | -2.413 |  | 1.961 |  | -0.143 |  | -1.230 |  | 0.224 |  |
|  |  | ICA_WM_8 |  | 2.327 |  | 2.118 |  | 0.129 |  | 1.099 |  | 0.277 |  |
|  |  | ICA_WM_9 |  | -2.765 |  | 1.903 |  | -0.170 |  | -1.453 |  | 0.152 |  |
|  |  | ICA_WM_10 |  | 3.218 |  | 1.917 |  | 0.194 |  | 1.679 |  | 0.099 |  |
|  |  | ICA_WM_11 |  | 1.892 |  | 1.943 |  | 0.112 |  | 0.974 |  | 0.335 |  |
|  |  | ICA_WM_16 |  | 3.140 |  | 1.872 |  | 0.197 |  | 1.677 |  | 0.099 |  |
|  |  | ICA_WM_19 |  | 2.081 |  | 1.974 |  | 0.124 |  | 1.054 |  | 0.297 |  |
|  |  | ICA_WM_24 |  | -2.357 |  | 1.964 |  | -0.139 |  | -1.200 |  | 0.235 |  |
|  |  | ICA_WM_25 |  | 6.218 |  | 2.121 |  | 0.340 |  | 2.931 |  | 0.005 |  |
| 19 |  | (Intercept) |  | 0.543 |  | 0.603 |  |  |  | 0.901 |  | 0.371 |  |
|  |  | ICA_WM_2 |  | -2.256 |  | 1.870 |  | -0.143 |  | -1.207 |  | 0.233 |  |
|  |  | ICA_WM_6 |  | 2.649 |  | 1.746 |  | 0.188 |  | 1.517 |  | 0.135 |  |
|  |  | ICA_WM_7 |  | -2.446 |  | 1.960 |  | -0.145 |  | -1.248 |  | 0.218 |  |
|  |  | ICA_WM_8 |  | 2.384 |  | 2.116 |  | 0.132 |  | 1.127 |  | 0.265 |  |
|  |  | ICA_WM_9 |  | -2.796 |  | 1.902 |  | -0.172 |  | -1.470 |  | 0.147 |  |
|  |  | ICA_WM_10 |  | 3.271 |  | 1.915 |  | 0.197 |  | 1.708 |  | 0.093 |  |
|  |  | ICA_WM_16 |  | 3.076 |  | 1.870 |  | 0.193 |  | 1.645 |  | 0.106 |  |
|  |  | ICA_WM_19 |  | 1.975 |  | 1.970 |  | 0.118 |  | 1.002 |  | 0.321 |  |
|  |  | ICA_WM_24 |  | -2.356 |  | 1.963 |  | -0.139 |  | -1.200 |  | 0.235 |  |
|  |  | ICA_WM_25 |  | 6.254 |  | 2.120 |  | 0.342 |  | 2.950 |  | 0.005 |  |
| 20 |  | (Intercept) |  | 0.544 |  | 0.603 |  |  |  | 0.903 |  | 0.371 |  |
|  |  | ICA_WM_2 |  | -2.090 |  | 1.862 |  | -0.133 |  | -1.122 |  | 0.267 |  |
|  |  | ICA_WM_6 |  | 2.899 |  | 1.728 |  | 0.206 |  | 1.677 |  | 0.099 |  |
|  |  | ICA_WM_7 |  | -2.426 |  | 1.960 |  | -0.144 |  | -1.238 |  | 0.221 |  |
|  |  | ICA_WM_8 |  | 2.450 |  | 2.115 |  | 0.136 |  | 1.158 |  | 0.252 |  |
|  |  | ICA_WM_9 |  | -2.716 |  | 1.900 |  | -0.167 |  | -1.429 |  | 0.159 |  |
|  |  | ICA_WM_10 |  | 3.392 |  | 1.911 |  | 0.204 |  | 1.775 |  | 0.082 |  |
|  |  | ICA_WM_16 |  | 3.154 |  | 1.868 |  | 0.198 |  | 1.688 |  | 0.097 |  |
|  |  | ICA_WM_24 |  | -2.257 |  | 1.961 |  | -0.133 |  | -1.151 |  | 0.255 |  |
|  |  | ICA_WM_25 |  | 6.325 |  | 2.119 |  | 0.346 |  | 2.985 |  | 0.004 |  |
| 21 |  | (Intercept) |  | 0.738 |  | 0.579 |  |  |  | 1.275 |  | 0.208 |  |
|  |  | ICA_WM_6 |  | 2.494 |  | 1.694 |  | 0.177 |  | 1.472 |  | 0.147 |  |
|  |  | ICA_WM_7 |  | -2.365 |  | 1.964 |  | -0.140 |  | -1.204 |  | 0.234 |  |
|  |  | ICA_WM_8 |  | 2.177 |  | 2.106 |  | 0.121 |  | 1.034 |  | 0.306 |  |
|  |  | ICA_WM_9 |  | -2.792 |  | 1.904 |  | -0.171 |  | -1.467 |  | 0.148 |  |
|  |  | ICA_WM_10 |  | 3.290 |  | 1.914 |  | 0.198 |  | 1.719 |  | 0.091 |  |
|  |  | ICA_WM_16 |  | 3.019 |  | 1.869 |  | 0.190 |  | 1.616 |  | 0.112 |  |
|  |  | ICA_WM_24 |  | -2.327 |  | 1.964 |  | -0.137 |  | -1.185 |  | 0.241 |  |
|  |  | ICA_WM_25 |  | 6.154 |  | 2.118 |  | 0.336 |  | 2.905 |  | 0.005 |  |
| 22 |  | (Intercept) |  | 0.857 |  | 0.567 |  |  |  | 1.510 |  | 0.137 |  |
|  |  | ICA_WM_6 |  | 2.238 |  | 1.677 |  | 0.159 |  | 1.334 |  | 0.187 |  |
|  |  | ICA_WM_7 |  | -2.261 |  | 1.963 |  | -0.134 |  | -1.152 |  | 0.254 |  |
|  |  | ICA_WM_9 |  | -2.699 |  | 1.903 |  | -0.166 |  | -1.419 |  | 0.162 |  |
|  |  | ICA_WM_10 |  | 3.365 |  | 1.913 |  | 0.203 |  | 1.759 |  | 0.084 |  |
|  |  | ICA_WM_16 |  | 3.110 |  | 1.868 |  | 0.195 |  | 1.665 |  | 0.102 |  |
|  |  | ICA_WM_24 |  | -2.275 |  | 1.965 |  | -0.134 |  | -1.158 |  | 0.252 |  |
|  |  | ICA_WM_25 |  | 6.258 |  | 2.117 |  | 0.342 |  | 2.956 |  | 0.005 |  |
| 23 |  | (Intercept) |  | 0.649 |  | 0.539 |  |  |  | 1.203 |  | 0.234 |  |
|  |  | ICA_WM_6 |  | 2.348 |  | 1.679 |  | 0.167 |  | 1.398 |  | 0.167 |  |
|  |  | ICA_WM_9 |  | -2.976 |  | 1.893 |  | -0.183 |  | -1.572 |  | 0.121 |  |
|  |  | ICA_WM_10 |  | 3.376 |  | 1.919 |  | 0.203 |  | 1.759 |  | 0.084 |  |
|  |  | ICA_WM_16 |  | 3.114 |  | 1.873 |  | 0.196 |  | 1.662 |  | 0.102 |  |
|  |  | ICA_WM_24 |  | -2.435 |  | 1.965 |  | -0.144 |  | -1.239 |  | 0.220 |  |
|  |  | ICA_WM_25 |  | 6.145 |  | 2.121 |  | 0.336 |  | 2.897 |  | 0.005 |  |
| 24 |  | (Intercept) |  | 0.723 |  | 0.539 |  |  |  | 1.342 |  | 0.185 |  |
|  |  | ICA_WM_6 |  | 2.163 |  | 1.680 |  | 0.154 |  | 1.287 |  | 0.203 |  |
|  |  | ICA_WM_9 |  | -2.961 |  | 1.901 |  | -0.182 |  | -1.557 |  | 0.125 |  |
|  |  | ICA_WM_10 |  | 3.223 |  | 1.924 |  | 0.194 |  | 1.675 |  | 0.099 |  |
|  |  | ICA_WM_16 |  | 3.192 |  | 1.881 |  | 0.201 |  | 1.697 |  | 0.095 |  |
|  |  | ICA_WM_25 |  | 6.077 |  | 2.130 |  | 0.332 |  | 2.853 |  | 0.006 |  |
| 25 |  | (Intercept) |  | 0.763 |  | 0.541 |  |  |  | 1.411 |  | 0.163 |  |
|  |  | ICA_WM_9 |  | -2.670 |  | 1.898 |  | -0.164 |  | -1.406 |  | 0.165 |  |
|  |  | ICA_WM_10 |  | 3.151 |  | 1.934 |  | 0.190 |  | 1.630 |  | 0.108 |  |
|  |  | ICA_WM_16 |  | 2.721 |  | 1.855 |  | 0.171 |  | 1.467 |  | 0.148 |  |
|  |  | ICA_WM_25 |  | 5.812 |  | 2.132 |  | 0.318 |  | 2.726 |  | 0.008 |  |
| 26 |  | (Intercept) |  | 0.053 |  | 0.195 |  |  |  | 0.272 |  | 0.787 |  |
|  |  | ICA_WM_10 |  | 3.178 |  | 1.949 |  | 0.191 |  | 1.630 |  | 0.108 |  |
|  |  | ICA_WM_16 |  | 2.600 |  | 1.868 |  | 0.163 |  | 1.392 |  | 0.169 |  |
|  |  | ICA_WM_25 |  | 5.954 |  | 2.147 |  | 0.325 |  | 2.774 |  | 0.007 |  |
| 27 |  | (Intercept) |  | 0.244 |  | 0.139 |  |  |  | 1.758 |  | 0.084 |  |
|  |  | ICA_WM_10 |  | 3.284 |  | 1.963 |  | 0.198 |  | 1.673 |  | 0.099 |  |
|  |  | ICA_WM_25 |  | 6.086 |  | 2.161 |  | 0.333 |  | 2.816 |  | 0.007 |  |
|  | | | | | | | | | | | | | |

**Talairach tables**

| **Talairach tables of the components significantly correlated with OCPD** | | | | | | |
| --- | --- | --- | --- | --- | --- | --- |
| **Area** | | **Broadmann**  **area** | | **Left/right**  **Volume (cc)** | | **Random effects**  **max value (x, y, z)** |
| **GM-05** | | | | | | |
| Medial Frontal Gyrus | 6, 8, 9, 10 | | 1.5/1.8 | | 6.4 (-3, 62, 16)/6.1 (3, 57, 5) | |
| Sub-Gyral | 6 | | 0.8/1.2 | | 4.1 (-30, -35, 38)/6.3 (37, 20, 20) | |
| Superior Frontal Gyrus | 8, 9, 10 | | 0.9/1.0 | | 4.9 (-3, 54, 25)/5.3 (3, 48, 31) | |
| Superior Temporal Gyrus | 22, 38, 42 | | 0.9/0.6 | | 4.6 (-67, -40, 16)/5.3 (49, 17, -7) | |
| Middle Frontal Gyrus | 8, 9, 10 | | 0.5/0.6 | | 4.3 (-21, 34, 38)/5.0 (36, 53, 21) | |
| Anterior Cingulate | 10, 32 | | 0.4/0.4 | | 4.6 (-3, 47, 3)/4.5 (3, 47, 6) | |
| Inferior Frontal Gyrus | 45, 47 | | 0.2/0.9 | | 3.4 (-42, 15, -11)/4.5 (43, 15, -13) | |
| Postcentral Gyrus | 40 | | 0.1/0.0 | | 3.2 (-65, -22, 18)/-999.0 (0, 0, 0) | |
| Middle Temporal Gyrus | 21, 39 | | 0.1/0.1 | | 3.1 (-65, -31, -4)/3.1 (36, -61, 27) | |
| Supramarginal Gyrus | * | | 0.0/0.1 | | -999.0 (0, 0, 0)/3.0 (50, -36, 34) | |
| **GM-23** | | | | | | |
| Culmen | * | | 4.5/6.7 | | 5.6 (-1, -45, -4)/8.3 (10, -36, -15) | |
| Fourth Ventricle | * | | 0.2/0.2 | | 4.0 (-1, -40, -22)/6.4 (1, -40, -19) | |
| Cerebellar Lingual | * | | 0.3/0.6 | | 5.2 (0, -44, -8)/6.1 (6, -44, -8) | |
| Sub-Gyral | * | | 1.0/1.9 | | 4.7 (-27, -46, 33)/5.7 (31, -36, 35) | |
| Declive | * | | 0.3/2.0 | | 3.5 (-9, -57, -11)/4.9 (9, -56, -11) | |
| Culmen of Vermis | * | | 0.2/0.0 | | 4.6 (0, -64, -7)/-999.0 (0, 0, 0) | |
| Precentral Gyrus | * | | 0.1/0.1 | | 4.1 (-34, 16, 35)/3.7 (37, 1, 28) | |
| Fastigium | * | | 0.0/0.2 | | -999.0 (0, 0, 0)/4.0 (10, -49, -19) | |
| Precuneus | 7 | | 0.3/0.0 | | 4.0 (-33, -64, 36)/-999.0 (0, 0, 0) | |
| Inferior Occipital Gyrus | * | | 0.1/0.2 | | 3.3 (-30, -82, -5)/4.0 (36, -76, -5) | |
| Supramarginal Gyrus | * | | 0.0/0.1 | | -999.0 (0, 0, 0)/3.8 (50, -43, 34) | |
| Medial Frontal Gyrus | * | | 0.1/0.0 | | 3.8 (-19, 39, 19)/-999.0 (0, 0, 0) | |
| Fusiform Gyrus | 37 | | 0.1/0.3 | | 3.7 (-36, -52, -11)/3.5 (40, -43, -13) | |
| Cerebellar Tonsil | * | | 0.1/0.1 | | 3.7 (0, -51, -35)/3.4 (6, -51, -37) | |
| Nodule | * | | 0.0/0.1 | | -999.0 (0, 0, 0)/3.5 (3, -48, -29) | |
| Superior Frontal Gyrus | 8 | | 0.2/0.1 | | 3.4 (-22, 42, 19)/3.4 (30, 30, 51) | |
| Inferior Parietal Lobule | * | | 0.1/0.1 | | 3.3 (-34, -52, 39)/3.4 (49, -29, 22) | |
| Middle Occipital Gyrus | * | | 0.1/0.0 | | 3.4 (-37, -77, 9)/-999.0 (0, 0, 0) | |
| Cingulate Gyrus | * | | 0.1/0.1 | | 3.0 (-12, -44, 28)/3.3 (15, -39, 31) | |
| Lingual Gyrus | * | | 0.0/0.1 | | -999.0 (0, 0, 0)/3.3 (10, -85, -2) | |
| Middle Frontal Gyrus | 6 | | 0.4/0.0 | | 3.3 (-31, 48, 2)/-999.0 (0, 0, 0) | |
| Uvula | * | | 0.0/0.1 | | -999.0 (0, 0, 0)/3.1 (25, -80, -23) | |
| Insula | * | | 0.0/0.1 | | -999.0 (0, 0, 0)/3.1 (46, -31, 20) | |
| Inferior Frontal Gyrus | * | | 0.0/0.1 | | -999.0 (0, 0, 0)/3.1 (37, 3, 32) | |
| Inferior Parietal Lobule | 40 | | 0.2/0.0 | | 4.4 (-45, -39, 39)/-999.0 (0, 0, 0) | |
| Angular Gyrus | * | | 0.1/0.0 | | 4.3 (-40, -66, 30)/-999.0 (0, 0, 0) | |
| Precentral Gyrus | * | | 0.0/0.1 | | -999.0 (0, 0, 0)/4.2 (40, 19, 35) | |
| Postcentral Gyrus | 5 | | 0.0/0.2 | | -999.0 (0, 0, 0)/4.0 (39, -42, 60) | |
| Inferior Frontal Gyrus | * | | 0.0/0.1 | | -999.0 (0, 0, 0)/4.0 (43, 37, 4) | |
| Declive | * | | 0.2/0.1 | | 3.7 (-45, -68, -21)/3.9 (45, -67, -22) | |
| Pyramis | * | | 0.1/0.0 | | 3.7 (-40, -66, -33)/-999.0 (0, 0, 0) | |
| Inferior Temporal Gyrus | * | | 0.1/0.0 | | 3.6 (-59, -12, -16)/-999.0 (0, 0, 0) | |
| Anterior Cingulate | 32 | | 0.1/0.0 | | 3.5 (-9, 32, -7)/-999.0 (0, 0, 0) | |
| Superior Parietal Lobule | 7 | | 0.0/0.1 | | -999.0 (0, 0, 0)/3.5 (30, -64, 54) | |
| **WM-25** | | | | | | |
| Middle Temporal Gyrus | 19, 20, 21, 37, 39 | | 1.7/3.3 | | 8.2 (-58, -34, -12)/10.4 (56, -33, -14) | |
| Inferior Temporal Gyrus | 20, 21 | | 0.9/1.2 | | 6.7 (-58, -31, -15)/7.4 (56, -30, -16) | |
| Precuneus | 7 | | 0.2/1.0 | | 4.9 (-22, -57, 50)/6.2 (25, -54, 52) | |
| Sub-Gyral | * | | 0.2/1.2 | | 3.8 (-21, -57, 54)/6.1 (52, -33, -10) | |
| Superior Parietal Lobule | 7 | | 0.4/1.0 | | 4.2 (-24, -60, 53)/5.9 (28, -55, 44) | |
| Middle Frontal Gyrus | 8, 9, 10 | | 0.7/1.1 | | 5.6 (-42, 50, 3)/5.5 (25, 41, 37) | |
| Medial Frontal Gyrus | 6, 10 | | 0.3/0.6 | | 4.9 (-7, -12, 60)/4.7 (13, 57, -5) | |
| Inferior Parietal Lobule | 40 | | 0.3/0.4 | | 3.7 (-58, -38, 36)/4.9 (39, -48, 52) | |
| Superior Frontal Gyrus | 6, 8, 9 | | 0.3/1.3 | | 3.7 (-10, 54, 26)/4.8 (10, 24, 51) | |
| Superior Temporal Gyrus | 39 | | 0.5/0.6 | | 4.2 (-50, -32, 8)/4.8 (46, -57, 29) | |
| Inferior Frontal Gyrus | 9, 46 | | 0.3/0.2 | | 4.2 (-45, 50, 0)/3.5 (52, 12, 30) | |
| Fusiform Gyrus | * | | 0.0/0.1 | | -999.0 (0, 0, 0)/4.2 (56, -33, -19) | |
| Middle Occipital Gyrus | 18 | | 0.2/0.0 | | 4.1 (-13, -88, 15)/-999.0 (0, 0, 0) | |
| Postcentral Gyrus | 3 | | 0.1/0.1 | | 3.9 (-59, -20, 37)/3.3 (52, -29, 40) | |
| Parahippocampal Gyrus | * | | 0.0/0.1 | | -999.0 (0, 0, 0)/3.9 (36, -16, -23) | |
| Precentral Gyrus | * | | 0.0/0.3 | | -999.0 (0, 0, 0)/3.7 (53, -11, 36) | |
| Cuneus | 18, 19 | | 0.3/0.1 | | 3.7 (-10, -88, 18)/3.4 (22, -81, 33) | |
| Supramarginal Gyrus | 40 | | 0.3/0.0 | | 3.5 (-56, -42, 30)/-999.0 (0, 0, 0) | |
| Paracentral Lobule | 5 | | 0.1/0.0 | | 3.4 (-9, -35, 54)/-999.0 (0, 0, 0) | |
| Cingulate Gyrus | 32 | | 0.1/0.0 | | 3.3 (-12, 25, 32)/-999.0 (0, 0, 0) | |
| Angular Gyrus | * | | 0.0/0.1 | | -999.0 (0, 0, 0)/3.2 (46, -60, 32) | |
| Inferior Semi-Lunar Lobule | * | | 0.1/0.0 | | 3.1 (-15, -69, -37)/-999.0 (0, 0, 0) | |
| Please note that WM nomenclature is identified in terms of adjacency to GM regions | | | | | | |
